# 15 Years of Progress on Transition Metal-Based Electrocatalysts for Microbial Electrochemical Hydrogen Production: From Nanoscale Design to Macroscale Application

**DOI:** 10.1007/s40820-025-01781-6

**Published:** 2025-06-18

**Authors:** Seyed Masoud Parsa, Zhijie Chen, Huu Hao Ngo, Wei Wei, Xinbo Zhang, Ying Liu, Bing-Jie Ni, Wenshan Guo

**Affiliations:** 1 Centre for Technology in Water and Wastewater, School of Civil and Environmental Engineering, University of Technology Sydney, Ultimo, NSW 2007 Australia; 2 UNSW Water Research Centre, School of Civil and Environmental Engineering, The University New South Wales, Sydney, NSW 2052 Australia; 3 Tianjin Key Laboratory of Aquatic Science and Technology, Tianjin Chengjian University, Jinjing Road 26, Tianjin, 300384 People’s Republic of China

**Keywords:** Bioelectrochemical systems, Hydrogen evolution reaction, Transition metal catalysts, Cost analysis, Life cycle assessment, Artificial intelligence design

## Abstract

Designing high-performance electrocatalysts is one of the key challenges in the development of microbial electrochemical hydrogen production. Transition metal-based (TM-based) electrocatalysts are introduced as an astonishing alternative for future catalysts by addressing several disadvantages, like the high cost and low performance of noble metal and metal-free electrocatalysts, respectively. In this critical review, a comprehensive analysis of the major development of all families of TM-based catalysts from the beginning development of microbial electrolysis cells in the last 15 years is presented. Importantly, pivotal design parameters such as selecting efficient synthesis methods based on the type of material, main criteria during each synthesizing method, and the pros and cons of various procedures are highlighted and compared. Moreover, procedures for tuning and tailoring the structures, advanced strategies to promote active sites, and the potential for implementing novel unexplored TM-based hybrid structures suggested. Furthermore, consideration for large-scale application of TM-based catalysts for future mass production, including life cycle assessment, cost assessment, economic analysis, and recently pilot-scale studies were highlighted. Of great importance, the potential of utilizing artificial intelligence and advanced computational methods such as active learning, microkinetic modeling, and physics-informed machine learning in designing high-performance electrodes in successful practices was elucidated. Finally, a conceptual framework for future studies and remaining challenges on different aspects of TM-based electrocatalysts in microbial electrolysis cells is proposed.

**Graphical Abstract:**

## Highlights

- Comprehensive overview of the evolution of transition metal-based catalysts in microbial electrolysis cells from their inception to the present.
- Critical design parameters of catalysts evaluated from technical, economic, and sustainability perspectives.
- A conceptual framework is proposed to address current challenges and guide future research based on literature best practices.

## Graphical abstract

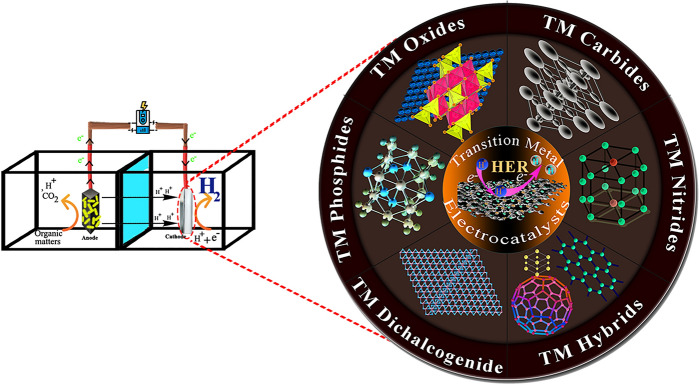

## Introduction

Innumerable action plans from the beginning of the new century were proposed by the United Nations (UN) on significant issues (like the lack of safe drinking water) [1] to address sustainable solutions for human being. At the forefront of these action plans are the Sustainable Development Goals (SDGs) of the UN Agenda 2030, which are outlined in 17 important Goals for addressing issues in front of human beings and the precious blue planet [2]. In this regard, the scientific communities across different disciplines, from engineering to social science, and even lawmakers have tried to take steps toward these 17 Goals. Of particular interest are Goal 6 and Goal 7, which focus on “Clean Water and Sanitation” and “Clean and Affordable Energy for All” and are considered as two of the most important goals that have multifaceted effects on human beings as well as the environment and a significant impact on realizing other SDGs [3, 4]. Interestingly, bioelectrochemical systems (BESs) such as microbial fuel cells (MFCs), microbial electrolysis cells (MECs), and microbial electrosynthesis (MESs) in some ways can satisfy both of these goals due to their mechanism which employs wastewater treatment (or CO_2_) to produce electricity, biohydrogen, and valuable chemicals correspondingly. Interestingly, Sayed et al. [5] studied the interlinkage between SDGs and plant-based BESs and reported that all 17 goals are directly or indirectly realized pone or more targets of each SDGs. However, among all BESs they only focused on the MFCs and their interconnection with other SDGs. Moreover, Kathori et al. [6] explicitly highlighted the role of MFC for wastewater treatment and electricity production and its direct relation on realizing of SDG7 and SDG13. Although the link between SDGs and MEC for hydrogen production was not explicitly examined in the literature, a number of researchers showed the substantial role of hydrogen in achieving SDGs [7–9]. For instance, El-Maroufi et al. [10] showed how the production of green hydrogen through integration of three hybrid renewable energy sources of photovoltaic panels, wind turbines, and biomass generators is considerable step on the realization of the SDG 13. It is important to point out that while wastewater is responsible for heavy pollution of transboundary river [11, 12] throughout the world, it also contains chemical energy in the form of organic matter which can be extracted via BESs. However, for BES long is the way and hard the journey that leads from a laboratory-scale idea to a real-life application. BESs schemes have come a long way from the beginning of the twentieth century when M.C. Potter in his laboratory for the first time realized that microorganisms transfer electrons when subjected to the decomposition of organic matter [13]. That was the first step in the development of BESs, and it was followed by the first prototype of microbial fuel cells (MFCs) by Barnett Cohen at Yale [14] in the early 30'. Time passed and the BESs entered a new realm in 2005 after the proposal of the first prototype of MECs for biohydrogen production by two research groups at Penn State and Wageningen University simultaneously [15, 16]. By presenting the concept of MEC, numerous advances have been made in recent years by introducing various MEC configurations and extending to other applications such as desalination, producing valuable chemicals, resource recovery, and integration with previous biological wastewater treatment schemes [17–22]. Intrinsically, the MEC is highly multidisciplinary in its nature; hence, different disciplines from biology to chemistry alongside with applied engineering should concurrently work to translate a lab prototype into real-world application. In this view, material science is one of the most important players in the development of MECs since it could act as a bridge between fundamental science and engineering. Perhaps this is one of the main reasons why a huge body of reviews with an explicit focus on applied materials in MECs have been written in recent years.

MECs consist of four main parts (the principle of MEC is concisely discussed in the next section), which are a power supplier, anode electrode, cathode electrode, and membrane (for double-chamber configuration), where the cathode is the site of producing hydrogen. In the early stages of MECs development, the most commonly used materials in many experiments for the cathode electrode were made of carbon-based materials coated with platinum (Pt) due to the unique characteristics of carbon and the high catalytic activity of Pt and other noble metals [23, 24]. This is quite reasonable because for preliminary experiments of a technology, materials with the highest performance from a purely technical viewpoint are selected to understand the mechanisms and the behavior of a system which means the cost is not a main objective in the early stages. However, precious metals are not a suitable candidate for real-world applications; thus, metal-free electrocatalysts such as fully carbon-based cathodes [25] were proposed as another strategy to address the high cost of noble metal catalysts such as Pt, Pd, Ru, among others. However, the lower catalytic activity was the main drawback of these types of catalysts. By the rise of transition metal (TM) catalysts such as nickel almost at the end of 2008, a huge body of research focused on this family of materials as a promising candidate because it meets both features of the noble metal and metal-free catalysts such as acceptable catalytic activity and low cost as well as being abundantly available, respectively. It is important to note that catalysts are cornerstones in crucial electrochemical reactions, from oxygen reduction reaction (ORR), oxygen evolution reaction (OER), and hydrogen evolution reaction (HER) to energy storage and environmental applications, just to name a few [26–31].

Currently, reviews on the catalysts of MECs have mainly cross on two avenues. The first avenue presents an overview of various types of applied materials (for both anode and cathode), such as nanomaterials and noble metals [23, 32–37], while some reviews have discussed specific type of cathode materials such as nickel [38]- and graphene-based catalysts [39]. The second approach is focused only on the biocatalysts and biocathodes [40–43]. Xu et al. [25] discussed several types of transition metal catalysts applied in MEC and presented an overview of each category; however, the study only covered half of the applied TM-based catalysts in MEC. Moreover, the study was limited to an overview of strategies for reducing free Gibbs energy (ΔG*), electrical conductivity, and the impact of biofilm formation on the catalyst. Yu's group [44] developed a framework on state-of-the-art mechanisms of microbial electrosynthesis cells for energy and valuable chemical production and concisely discussed the mechanism of electron transfer in microbial cathodes. Zhen et al. [45] in a critical review discussed various configurations of MEC integration designs and focused on the mechanism of extracellular electron transfer in electrodes. The above reviews comprise all the published articles on the topic of cathode and catalyst materials of MEC with a general approach, as discussed. However, there is still no critical review article that explicitly focuses on the most important type of electrocatalysts of MEC—transition metal electrocatalysts—and covers all aspects of this catalyst.

**Fig. 1:**
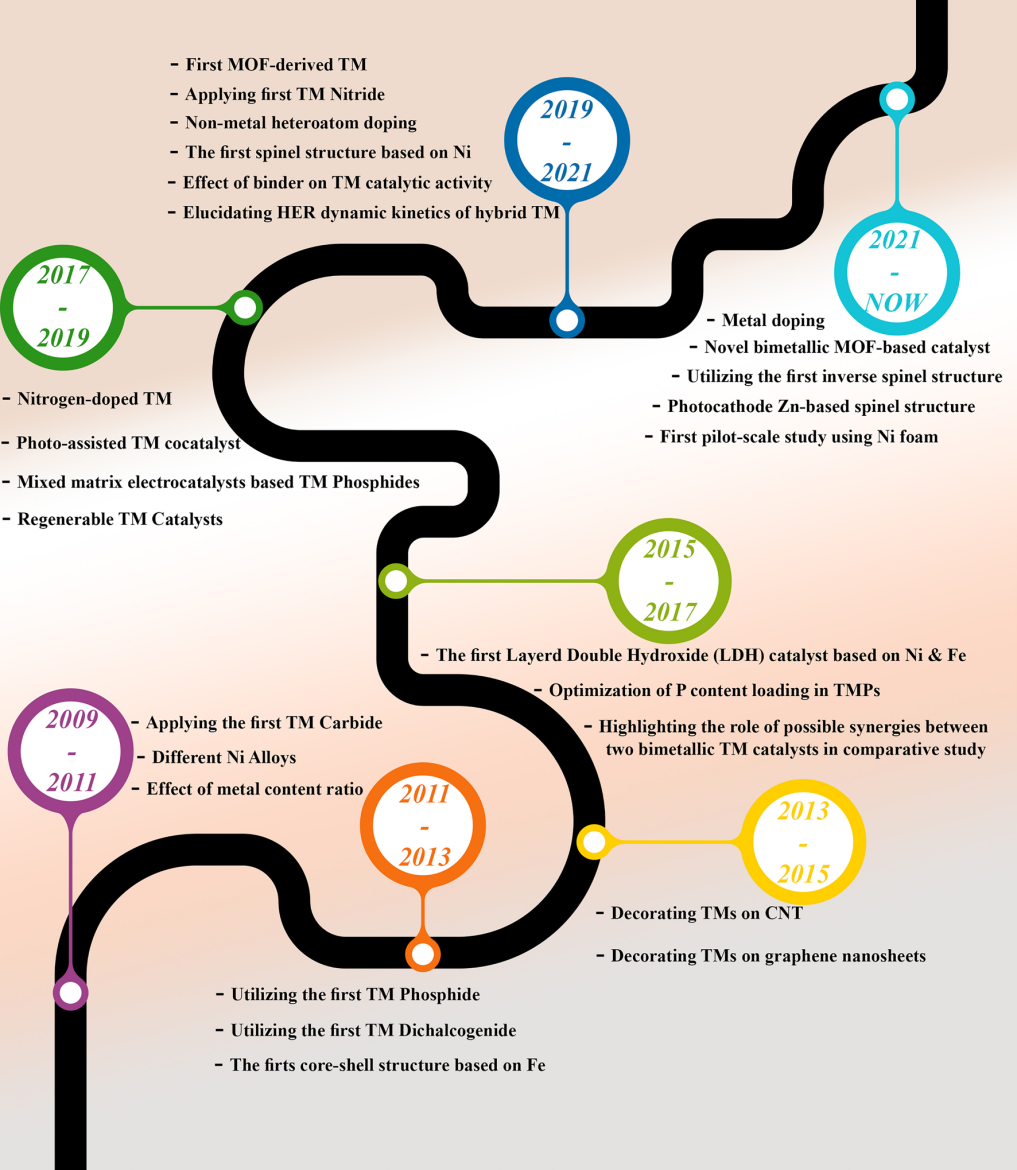
Historical roadmap on the developments and milestones of utilizing different transition metal-based electrocatalysts in MECs from 2009 to 2024

In this review, we critically discuss on 15 years of progress and development (Fig. 1) of all types of TM-based electrocatalysts from different categories, including transition metal oxides (TMO), transition metal dichalcogenides (TMD), transition metal phosphides (TMP), transition metal carbides (TMC), transition metal nitrides (TMN), hybrid transition metal structures, and cover all aspects from nanoscale design to macroscale application from the starting point of their development.

Importantly, we delivered an in-depth analysis of various types of synthesis methods and their conditions to highlight the pros and cons of each preparation while elucidating crucial and influential parameters of methods (positive or negative) that lead to enhance the performance of TM-based catalysts. Moreover, the strategies for improving the electrocatalytic characteristics of TM catalysts such as bandgap engineering, lowering internal/external resistance, metal/non-metal heteroatom (i.e., nitrogen, sulfur, phosphorus, copper, etc.) doping, physical/chemical activation and surface engineering, the significance of reducing ΔG*, optimizing metal contents, and low-cost approaches toward promoting active sites have been discussed. Furthermore, we thoroughly examined the large-scale applications of electrodes by focusing on the cost, economic factors, and life cycle assessment (LCA) (from cradle to gate) of TM-based electrocatalysts based on applied materials in order to examine the obstacles for translating catalysts in the future into real-world application. The pilot-scale studies and scaling-up approaches of TM electrodes were also highlighted, and possible forward strategies for large-scale implementation were determined.

Eventually, we scrutinized the current state-of-the-art applications of data-driven methods in MECs and illustrated the future role of artificial intelligence (AI) methods in the fabrication of high-performance TM catalysts. Explicitly, the most promising AI approaches for designing TM-based catalysts for MEC by considering the operating conditions of the system, the limited number of available data, and the practicality of exceptional AI methods such as active learning and physics-based machine learning for future catalyst fabrication based on successful practices in similar electrochemical energy fields were thoroughly discussed.

**Fig. 2:**
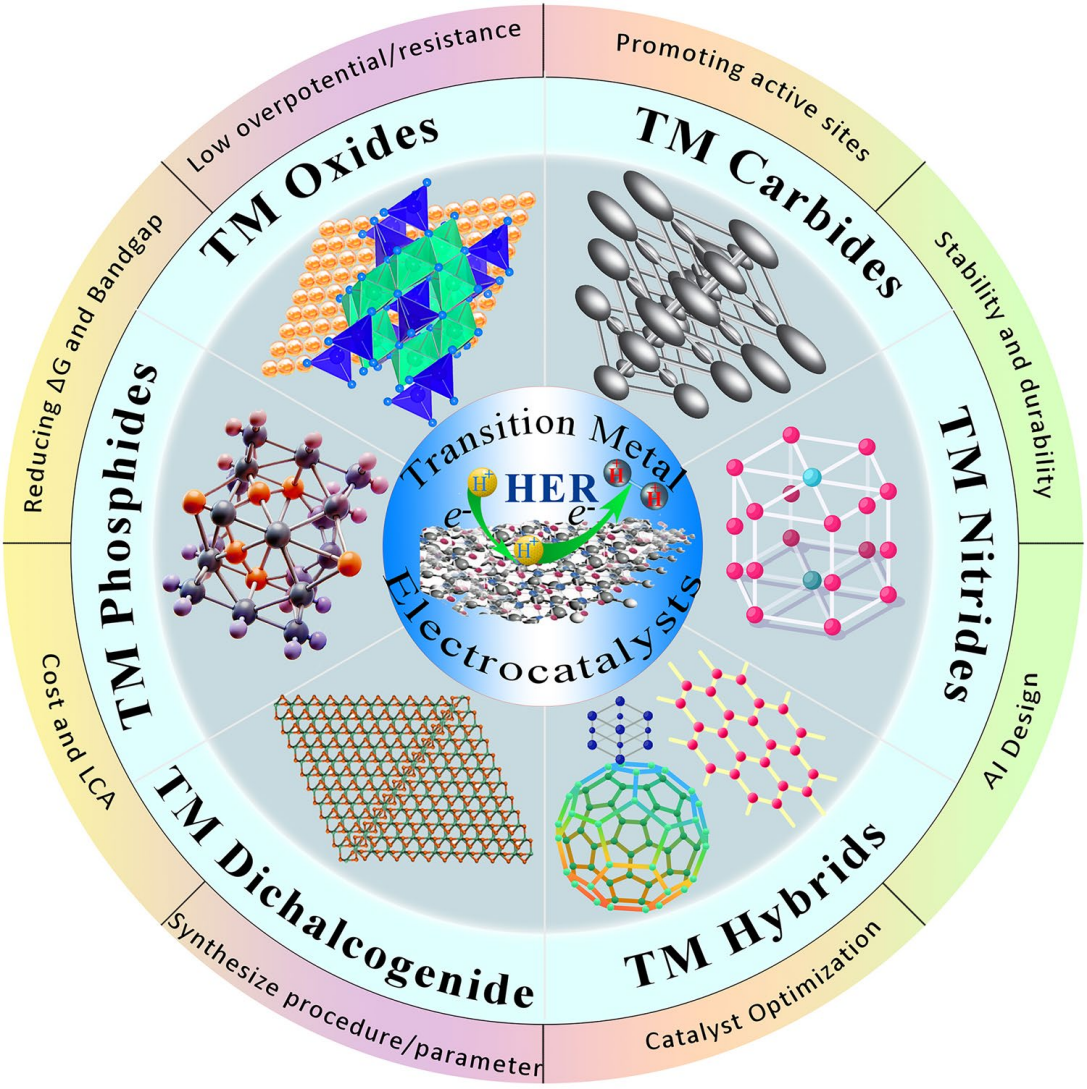
General concept of various families of transition metal-based electrocatalysts employed in MECs and important parameters associated in the context of present review

At the end of this review, a roadmap is proposed based on the key challenges facing TM-based electrocatalysts, outlining future directions and opening new avenues for research: (i) synthesizing methods and procedures; (ii) applying novel TM-based structures and design parameters toward further synergies; (iii) strategies for large-scale utilization of TM-based electrodes; (iv) facile, accessible, and low-cost strategies to enhance active sites and electrochemical properties of TM catalysts; (v) the significance of employing advanced theoretical approaches in combination with experiments alongside the importance of structural characterization catalysts; and (vi) implementing novel strategies based on successful practices of AI models for designing high-performance TM-based electrodes. Figure 2 illustrates a general overview of the main aspects of this review.

## Microbial Electrolysis Cells

**Fig. 3:**
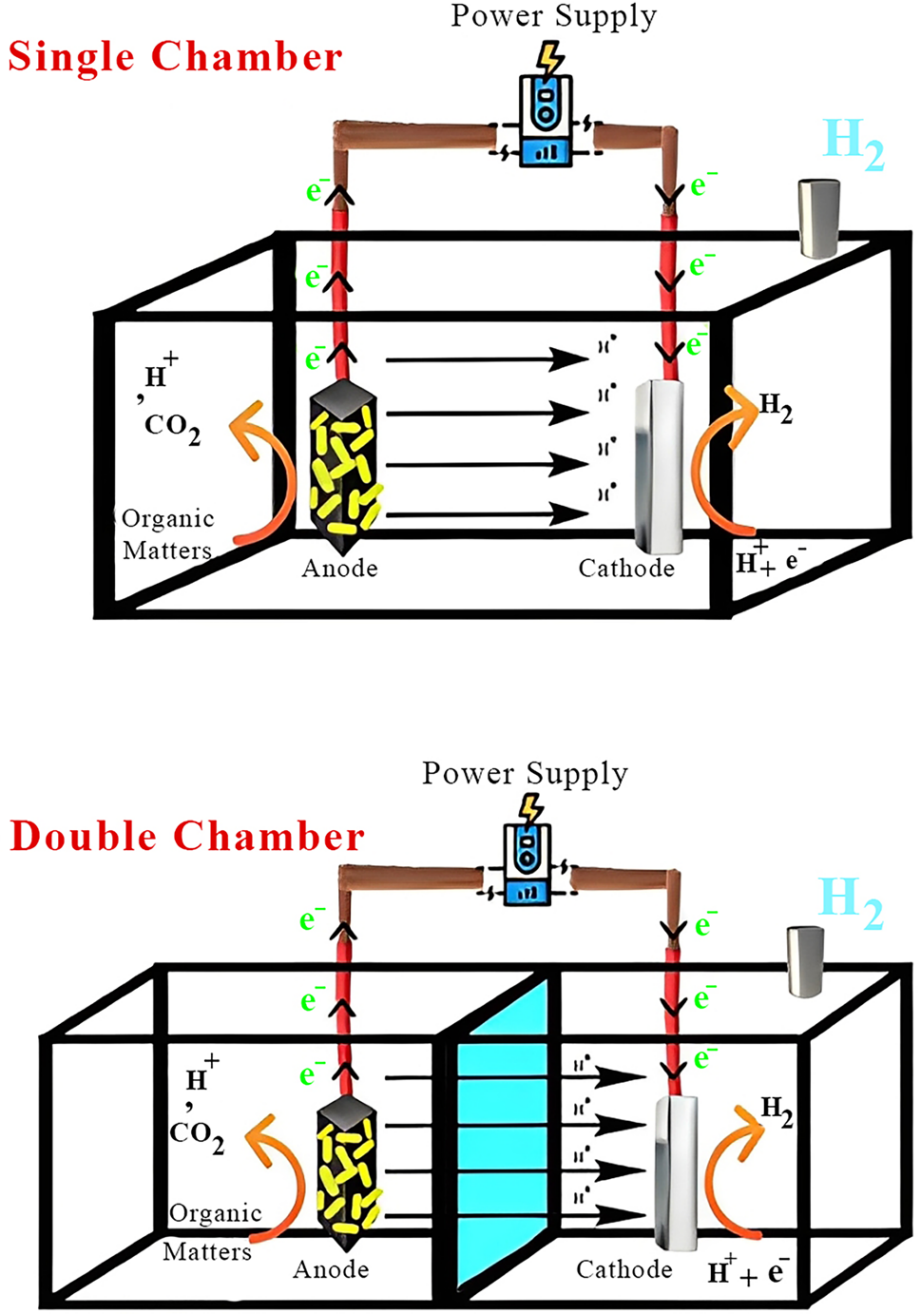
Schematic diagram of single- and double-chamber MECs

MECs are the latest promising technology proposed for wastewater treatment, biohydrogen production, and in some cases, value-added chemicals (Fig. 3). They consist of an anode, cathode, membrane, and a power supply. The general principle of MECs is as follows: Electro-active microorganisms accumulate on the surface of the anode and break down wastes or organic materials into electrons, protons, and carbon dioxide. Afterward, microorganisms transfer electrons and protons to the surface of the anode and MEC’s solution, respectively. Considering sodium acetate as the medium, the reaction on the anode can be described as:

$${\text{CH}}_{3} {\text{COO}}^{ - }  + 4{\text{H}}_{2} {\text{O}} \to 2{\text{HCO}}_{3} ^{ - }  + 9{\text{H}}^{ + }  + 8{\text{e}}^{ - }$$

In the meantime, electrons are assisted by the power supply and transferred via a wire to the cathode where they merge with protons in the solution to produce hydrogen, following the cathode reaction below [46]:

$$ {\text{8H}}^{ + } + {\text{8e}}^{ - } \to {\text{4H}}_{{2}} \quad \quad ({\text{E}}_{{{\text{C}}.{\text{P}}}} = - 0.{\text{414 V}}) $$

Importantly, the above reaction is not spontaneous and a potential >–0.414 is needed to combine electrons and protons under standard conditions ($\text{pH}=7;\text{Temperature}=25^\circ C; {P}_{\text{Hydrogen}}=1 \text{atm}$) [16, 47].

Regarding the Nernst equation, potential reduction during reaction at each half cell of cathode at standard conditions can be presented as [46]:

$${E}_{\text{Cathode}}={E}_{\text{cathode}}^{0}-\frac{RT}{2F}ln\frac{{P}_{\text{hydrgen}}}{{\left[{H}^{+}\right]}^{8}}=0- \frac{8.314\times 298.15}{2\times 96485}\text{ln}\frac{1}{{\left[{10}^{-7}\right]}^{8}}=-0.414 V$$

In the above equation ${E}_{cathode}^{0}$, R, T, and F represent hydrogen’s electrode potential, universal gas constant, temperature, and Faraday’s constant, respectively. Furthermore, for theoretical reduction potential reaction at the anode, the following can be presented:

$${E}_{\text{Anode}}={E}_{\text{Anode}}^{0}-\frac{RT}{8F}\text{ln}\frac{\left[{{CH}_{3}COO}^{-}\right]}{{\left[{HC{O}_{3}}^{-}\right]}^{2}{\times \left[{H}^{+}\right]}^{9}}$$

$$=0.187-\frac{8.314\times 298.15}{2\times 96485}\text{ln}\frac{0.0169}{{\left[0.005\right]}^{2}{\left[{10}^{-7}\right]}^{9}}=-0.3000 V$$

In the above equation, ${E}_{\text{Anode}}^{0}$ taken as 0.187 V for oxidizing acetate with the solution of ${{\text{HCO}}_{3}}^{-}=0.005 M$ and ${{\text{CH}}_{3}\text{COO}}^{-}=0.0169\text{M}$ at $\text{pH}=7$. Collectively, the minimum cell voltage in MEC at cathode for producing hydrogen can be presented as follows:

$${E}_{\text{Cell}}={E}_{\text{Cathode}}- {E}_{\text{Anode}}=\left(-0.414V\right)-\left(-0.300V\right)=-0.114 V$$

The negative value of the cell's voltage indicates that the process of hydrogen production by acetate is not spontaneous and an initial external voltage greater than 0.114 V is required. However, it should be noted that this is a theoretical value, whereas in practical applications, because of several losses during the process, such as mass transport loss, ohmic loss, activation loss, and microorganisms' metabolic losses, the applied voltage should be higher than 0.114 V. Previous studies have shown that an applied voltage of around ≥ 0.2 V is required [48]. Nonetheless, this is 6–10 times lower compared to the typical methods of water electrolysis, which require voltages in the range of 1.2–2 V [49].

## Principle of Hydrogen Evolution Reaction

**Fig. 4:**
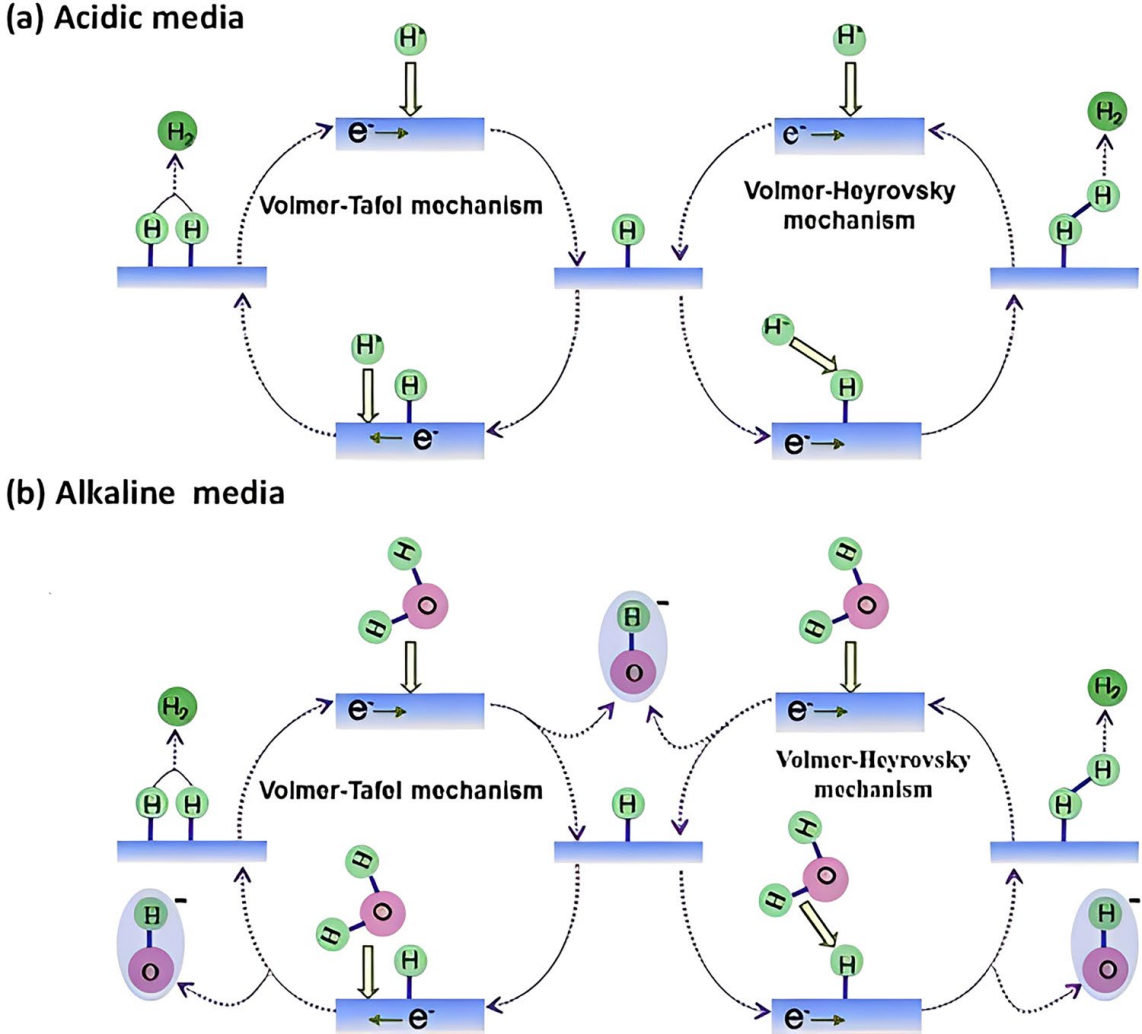
Possible mechanisms of hydrogen evolution reaction in different media. **a** Acidic environment and **b** alkaline environment. Reprinted from [281] with permission from Elsevier

The hydrogen evolution reaction is a critical electrochemical process in hydrogen production technologies including electrolyzers, photoelectrocatalytic cells, and MECs. Understanding the mechanisms that govern, the HER is fundamental for developing efficient catalysts that can operate under various conditions. The HER can proceed via two primary reaction mechanisms: (i) the Volmer–Heyrovsky mechanism and (ii) the Volmer–Tafel mechanism. Each of these mechanisms is governed by specific adsorption and desorption steps under different conditions (as shown in Fig. 4) and is influenced by different parameters such as the catalyst's surface properties, hydrogen binding energies, and reaction environments.

Thus, the HER mechanism typically follows either the Volmer–Heyrovsky or Volmer–Tafel pathways which are thoroughly presented in the literature [50–53]:

Volmer reaction (proton adsorption):

In acidic medium:

$$ {\text{H}}_{{3}} {\text{O}}^{ + }_{{({\text{aq}})}} + {\text{ e}}^{ - } + * \rightleftharpoons {\text{H}}^{ * } + {\text{H}}_{{2}} {\text{O}}_{{({\text{l}})}} $$

In alkaline medium:

$$ {\text{H}}_{{2}} {\text{O}}_{{({\text{l}})}} + {\text{e}}^{{ - }{}} +^{ * } \rightleftharpoons {\text{H}}^{ * } + {\text{OH}}^{ - }_{{({\text{aq}})}} $$

Heyrovsky reaction (electrochemical desorption):

In acidic medium:

$$ {\text{H}}^{ * } + {\text{H}}_{{3}} {\text{O}}^{ + }_{{({\text{aq}})}} + {\text{ e}}^{ - } \to {\text{H}}_{{{2}({\text{g}})}} + {\text{H}}_{{2}} {\text{O}}_{{({\text{l}})}} +^{ * } $$

In alkaline medium:

$$ {\text{H}}^{ * } + {\text{H}}_{{2}} {\text{O}}_{{({\text{l}})}} + {\text{e}}^{ - } \to {\text{H}}_{{{2}({\text{g}})}} + {\text{OH}}^{ - }_{{({\text{aq}})}} +^{ * } $$

Tafel reaction (chemical desorption):

In acidic or alkaline medium:

$$ {\text{H}}^{ * } + {\text{H}}^{ * } \to {\text{H}}_{{\text{2(g)}}} + {2}^{ * } $$

The rate of HER is determined by the slowest step in the reaction mechanism, which varies based on the electrode material and solution pH. The reaction-determining step (RDS) is often the Volmer step on catalysts with low hydrogen adsorption energy and the Heyrovsky or Tafel step on catalysts with high hydrogen adsorption energy.

## Materials Selection Criteria in MECs

**Fig. 5:**
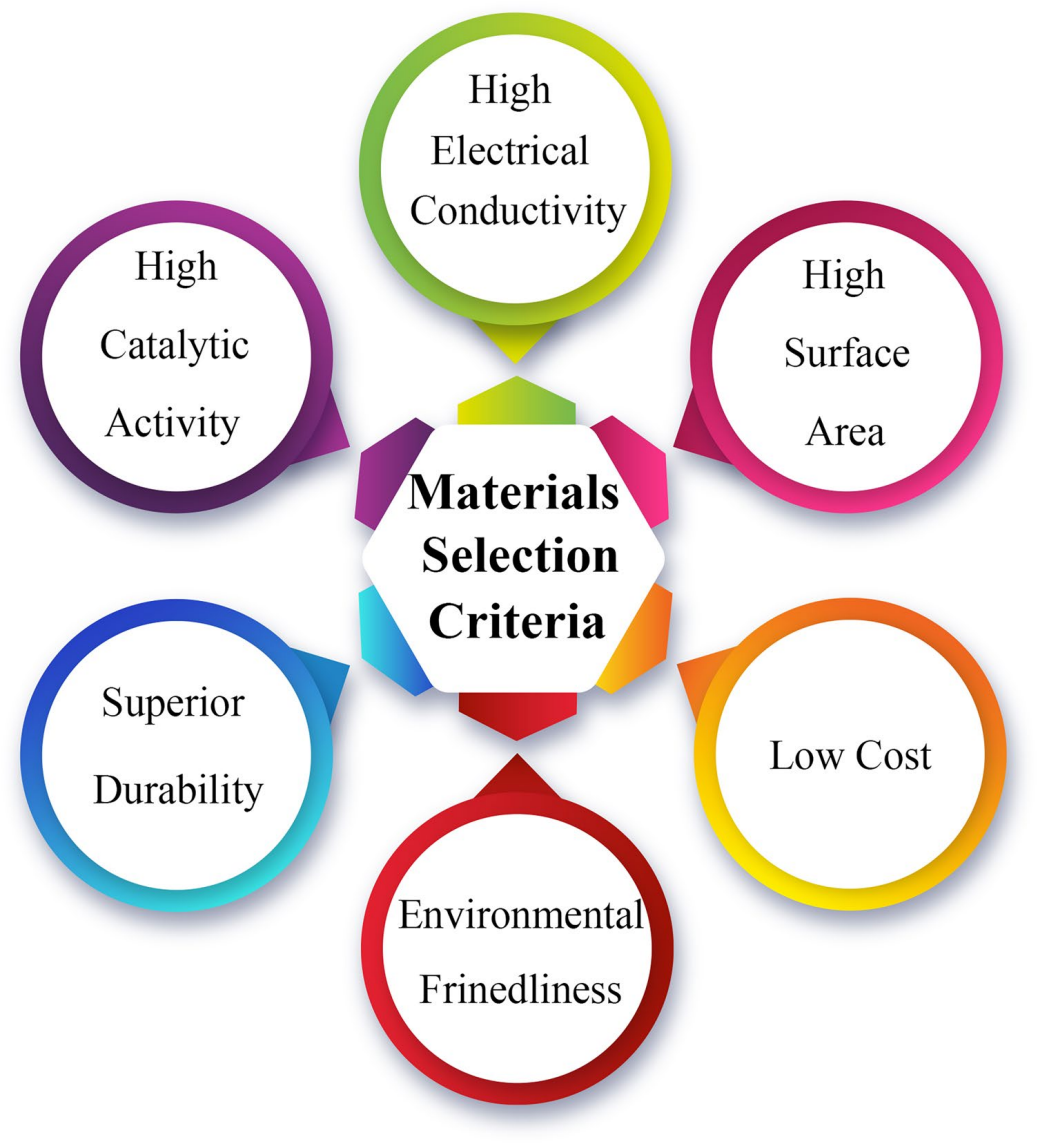
Electrode selection criteria

Generally, materials for cathode electrodes in MECs should satisfy several criteria, including superior catalytic activity, high surface area, high electrical conductivity, low cost, environmental friendliness, durability, antifouling, and biocompatibility (Fig. 5). Notably, durability and anti-biofouling are of the greatest importance and are well known as two of the most important obstacles to high-efficiency electrodes. Most of the previously utilized materials exhibit several pros related to the aforementioned criteria but also have several disadvantages attributed to them. However, it should be mentioned that the weighting of these criteria is variable and could be defined based on the context and applications of the MEC. For instance, assuming an MEC aims to produce energy and treat the wastewater in a space station; in such scenario, the most important parameter is durability, not the cost. However, when it aims to operate at a large scales for wastewater treatment and bioenergy production, the cost and economic viability takes priority.

It is important to note that some of these criteria have overlap with each other and promoting one of them has synergistic effect on the others.

### Boosting Catalytic Activity

**Fig. 6:**
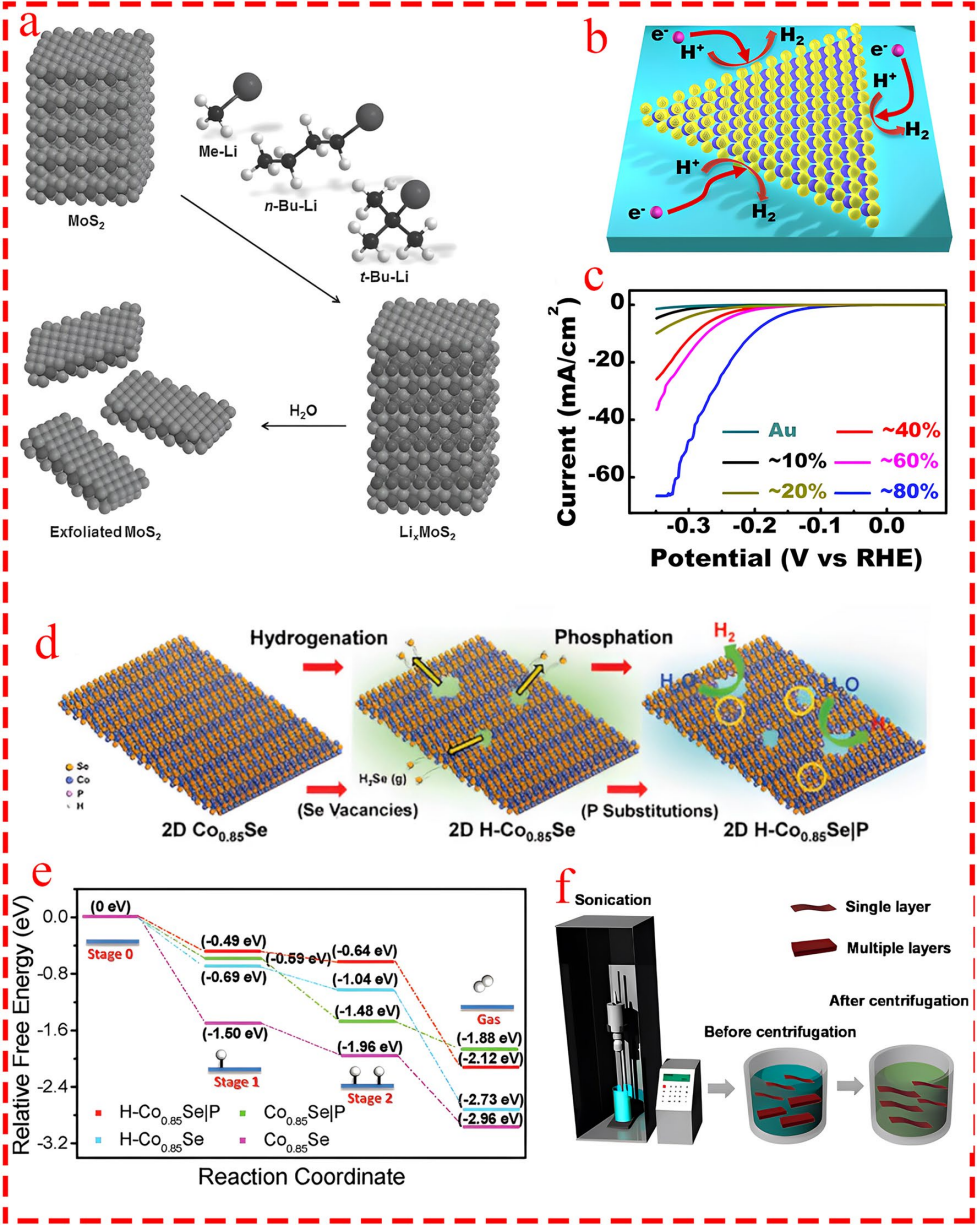
**a** Exfoliating process of bulk MoS_2_ with various types of organolithium compositions. Reprinted from [57] with permission from John Wiley and Sons. **b** Schematic of MoS_2_ grown on Au substrate [58]. **c** Coverage-dependent polarization curves. Reprinted from [58] with permission from American Chemical Society. **d** Schematic illustration for the synthesis process of H–Co_0.85_Se|P. Reprinted from [59] with permission from John Wiley and Sons. **e** Free energy pathways of the HER of Co_0.85_Se, H–Co_0.85_Se, Co_0.85_Se|P, and H–Co_0.85_Se|P. Reprinted from [59] with permission from John Wiley and Sons. **f** Schematic of synthesizing TMD nanosheets through liquid exfoliation procedure. Reprinted from [56] with permission from American Chemical Society

A wide range of strategies have been applied to increase catalytic activity. One of the most well-established strategies in this context is promoting active sites through various approaches. One direct approach to expose the embedded active sites is to thin the layer by well-established methods such as exfoliation [54, 55]. Nguyen et al. [56] elucidated that by liquid exfoliation through facile ultrasonication procedure of MoS_2_ sheets (Fig. 6f), the structure achieved a much larger specific area, exposing more active sites. Pumera group [57] employed various organolithium compounds to exfoliate bulk MoS_2_ (Fig. 6a) and discovered that larger organic compounds, such as t-BuLi and n-BuLi, produced larger anions, facilitating the intercalation of Li⁺ and effectively reducing the number of MoS_2_ layers. This process exposed more active sites during intercalation and resulted in substantial improvement in HER performance. Apart from exfoliation, Shi et al. [58] grew monolayer MoS_2_ on Au using the chemical vapor deposition (CVD) method, which provided greater control on the synthesis procedure (Fig. 6b), and reported nearly 80% area coverage of the decorated layer on Au. This demonstrated the best catalytic activity with an η of nearly ~ 25 times greater than the bulk scenario (Fig. 6c). Defect engineering, such as promoting anion vacancies to introduce defect sites in chalcogenides/oxides, is another frequently employed approach to enhance the catalytic activity of TM composites for HER. For instance, Hou et al. [59] presented an innovative ternary electrocatalyst of porous cobalt phosphoselenide nanosheets for efficient water splitting. The two-step synthesis process consisted of the hydrogenation of Co_0.85_Se nanosheets to create selenide-deficient H–Co_0.85_Se, followed by phosphorization to replace some selenium atoms with phosphorus (Fig. 6d). The Se-deficient, P-doped Co_0.85_Se structure exhibited highly efficient catalytic activity for HER, with the fastest kinetic activity in both steps among all of the simulated structures (Fig. 6e).

### Superior Electrical Conductivity

**Fig. 7:**
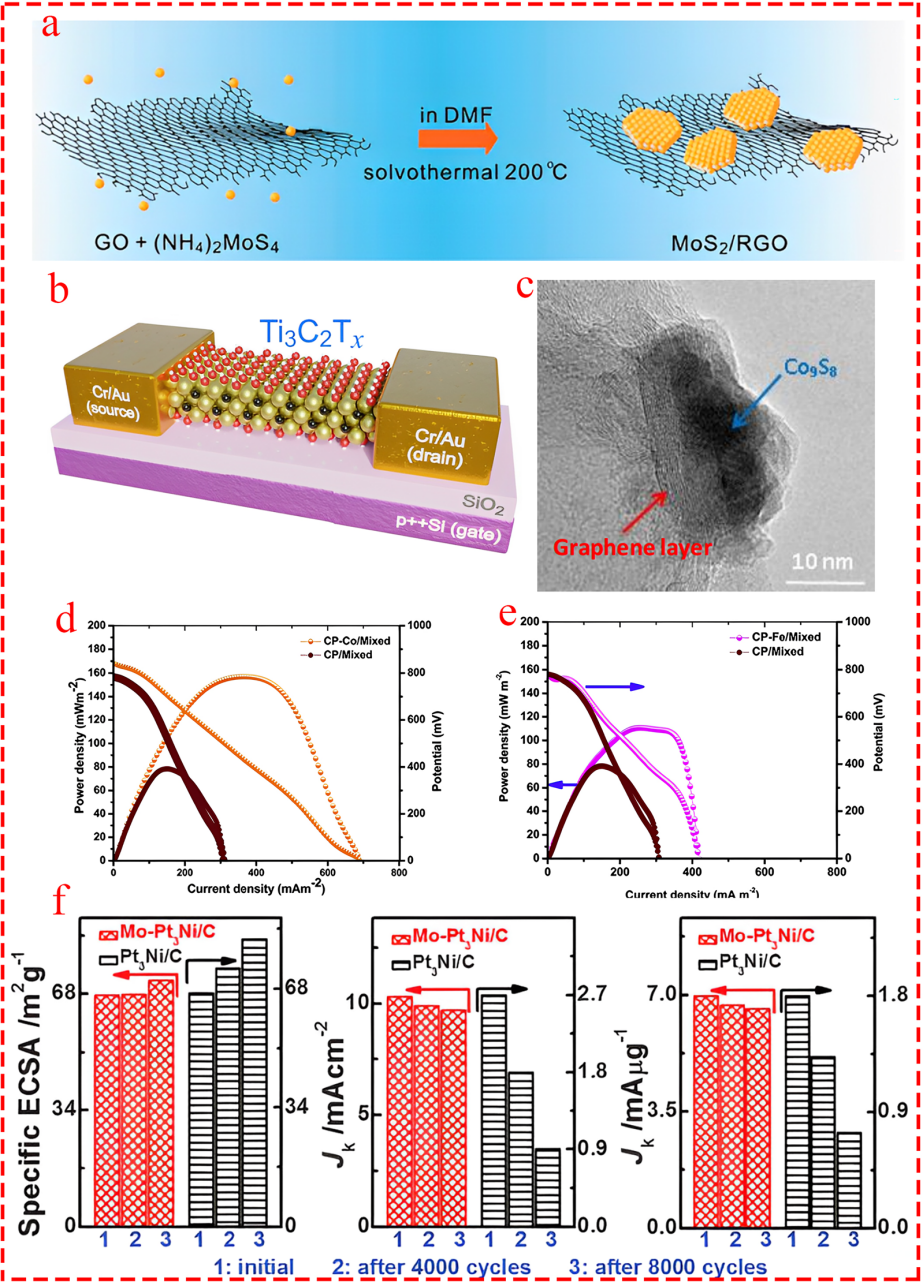
**a** Synthesizing procedure of the MoS_2_/rGO composite electrocatalyst. Reprinted from [70] with permission from American Chemical Society. **b** Scheme of a two-terminal device with a Ti_3_C_2_T_x_ channel. Reprinted from [69] with permission from Elsevier. **c** High-resolution TEM image of a Co_9_S_8_ particle after acid washing and the second heat treatment where graphene-like layers covering the particle are observed. Reprinted from [76] with permission from American Chemical Society. **d** and **e** Influence of the modified carbon electrode anode with cobalt and iron on power generation of MFC. Reprinted from [81] with permission from Elsevier. **f** The variation in electrochemically active surface areas (ECSAs) (left), specific activities (middle), and mass activities (right) of the octahedral Mo‐Pt_3_Ni/C catalyst and octahedral Pt_3_Ni/C catalyst before, after 4000, and after 8000 potential cycles. The durability tests were carried out at room temperature in O_2_‐saturated 0.1 M HClO_4_ at a scan rate of 50 mV s.^−1^. Reprinted from [77] with permission from American Association Advancement in Science (AAAS)

Combining TM-based electrocatalysts with conductive species such as carbon families including graphene [60–62], carbon paper [63, 64], and metallic substrate [65–67] is a widely adopted method to increase electronic conductivity. Indeed, coupling TM electrocatalysts with these species provides a conductive network with colossal channels for internal electron transport, leading to enhanced electrochemical active surface area for HER. For example, Sinitski's research group demonstrated that by employing a novel approach of excessing aluminum during the synthesis of Ti_3_AlC_2_ [68]. The electrical conductivity of monolayer flake Ti_3_C_2_T_x_ MXene was substantially improved. The monolayer flake Ti_3_C_2_T_x_ was placed between two terminals of Cr/Au to measure its electronic properties (Fig. 7b). The findings indicated that the average resistivity for the nine measured monolayer MXene devices was 1.14 ± 0.21 µΩ m^−1^, with the lowest value around 0.9 µΩ m^−1^. These values correspond to an average conductivity of 9050 ± 1620 S cm^−1^ and a maximum conductivity of 11,000 S cm^−1^, respectively [69]. However, it is important to note that one of the main drawbacks of MXenes is the decline of electrical conductivity due to oxidation. Importantly, Dai's group demonstrated that incorporating MoS_2_ nanoparticles with graphene (Fig. 7a) toward highly efficient HER resulted in exceptional improvement in electronic properties, in which electrical coupling to the underlying 2D substrate in an interconnected conducting framework provided fast electron transport from the less-conducting MoS_2_ nanoparticles to the electrodes [70].

### Improving Durability and Stability

Durability and stability are cornerstones for practical application of electrocatalysts in different aspects of electrochemical energy conversion and storage [71–74]. For instance, one of the obstacles facing metal–nitrogen–carbon electrocatalysts in fuel cell applications is their low durability. While strategies such as minimizing the rate of H_2_O_2_ intermediate production have been suggested, this approach has mainly remained at the lab scale [75]. Employing heteroatom doping (metal and non-metal) has been suggested as one of the highly promising methods to enhance the durability of electrocatalysts. Zhang et al. [76] showed synergies in nitrogen-doped cobalt carbon electrocatalysts for HER in universal pH conditions, with minimal performance loss after 1000 cycles. They showed that encapsulation of Co_9_S_8_ nanoparticles by a graphene-like layer (Fig. 7c) protected active sites, while Co–N bonds and graphitic N ensured stable catalytic performance. The catalyst exhibited similar performance in alkaline media, with only a 35 mV increase in overpotential. It is important to note that one well-established and prevalent method for improving surface area is utilizing heteroatom doping (with nitrogen at the forefront [31]). This approach is also effective for boosting active sites. In this regard, nitrogen can be considered a silver bullet with multi-purpose applications in the design of high-performance catalysts. In an important study, Huang et al. [77] doped Pt_3_Ni octahedra with various transition metals (vanadium, chromium, manganese, iron, cobalt, molybdenum, tungsten) to enhance the catalytic activity and durability of Pt-Ni catalysts, in which Mo outperformed than others. Moreover, the exceptional performance of Mo-doped Pt_3_Ni/C (Mo-Pt_3_Ni/C) compared to undoped Pt_3_Ni/C not only compared from catalytic activity but specifically examined from durability prospect. The findings revealed that after 8,000 potential cycles, Mo-Pt_3_Ni/C maintained 94.5% of its specific activity and 94.7% of its mass activity, while the undoped catalyst showed drastic declines to 33% and 41%, respectively (Fig. 7f). The enhanced durability is attributed to Mo's stabilizing role which was prevented the Ni leaching and maintaining structural integrity.

### Biocompatibity and Environmental Friendliness

Non-toxicity and biocompatibility are other criteria for TM-based electrocatalysts. Several MXene compositions have been shown to be biocompatible and non-toxic [78]. As a well-established fact, carbon and nitride are fundamental elements in the structure of biological organisms [79]. Moreover, several transition metals, like iron and zinc, are biocompatible with microorganisms because they are essential micronutrients involved in vital biological processes. Iron is crucial for electron transport, while zinc is necessary for enzyme function and gene regulation [80]. Mohammed et al. doped various transition metals (iron and cobalt) into the anode (carbon paper) of microbial fuel cells to improve the extracellular electron transfer mechanism. Their results showed that current density increased by about 210% and 140% (Fig. 7d, e), respectively, while COD removal and coulombic efficiency also improved significantly [81].

### Cost of Catalysts

For the matter of material costs, a specific discussion in a separate section is presented; however, a concise explanation here is necessary. Esposito and co-workers [82] showed that instead of using pristine noble metal catalysts for HER like Pt, the fabrication of a core–shell nanostructure where transition metal carbide (W_2_C) acts as the core and noble metal (Pt) as the shell significantly reduces the cost of the catalyst while the catalyst's performance remains acceptable and competitive with its counterparts. Since non-noble heteroatom doping plays an important role in catalysts, the cost associated with them can vary significantly case by case. For instance, Zhang et al. [83] doped nitrogen in activated carbon using cyanamide as the nitrogen source and reported the cost of the catalyst to be around 6.4 $ g^−1^, which was nearly 10% of the cost of normal Pt/C catalysts. Interestingly, they identified that the nitrogen source contributed to more than 91% of the total catalyst's cost; hence, using low-cost sources like melamine and urea would significantly decrease the cost of catalysts. Importantly, Farina and co-workers [84], declared that the current strategies in the design of catalysts could not translate to real-world applications and will not be used in industry, since all the adopted approaches for improving catalyst performance increase the cost of the final product. Indeed, they argued that the matter of cost in catalyst design is overlooked.

## Engineered Transition Metal-based Electrocatalysts in MECs

The emergence of TM-based electrocatalysts in MEC was the result of their unique performance, which combines the features of noble metal catalysts, but at lower cost and abundantly available, while addressing the obstacles of metal-free catalysts by providing acceptable catalytic activity and durability.

### Transition Metal Oxides

**Fig. 8:**
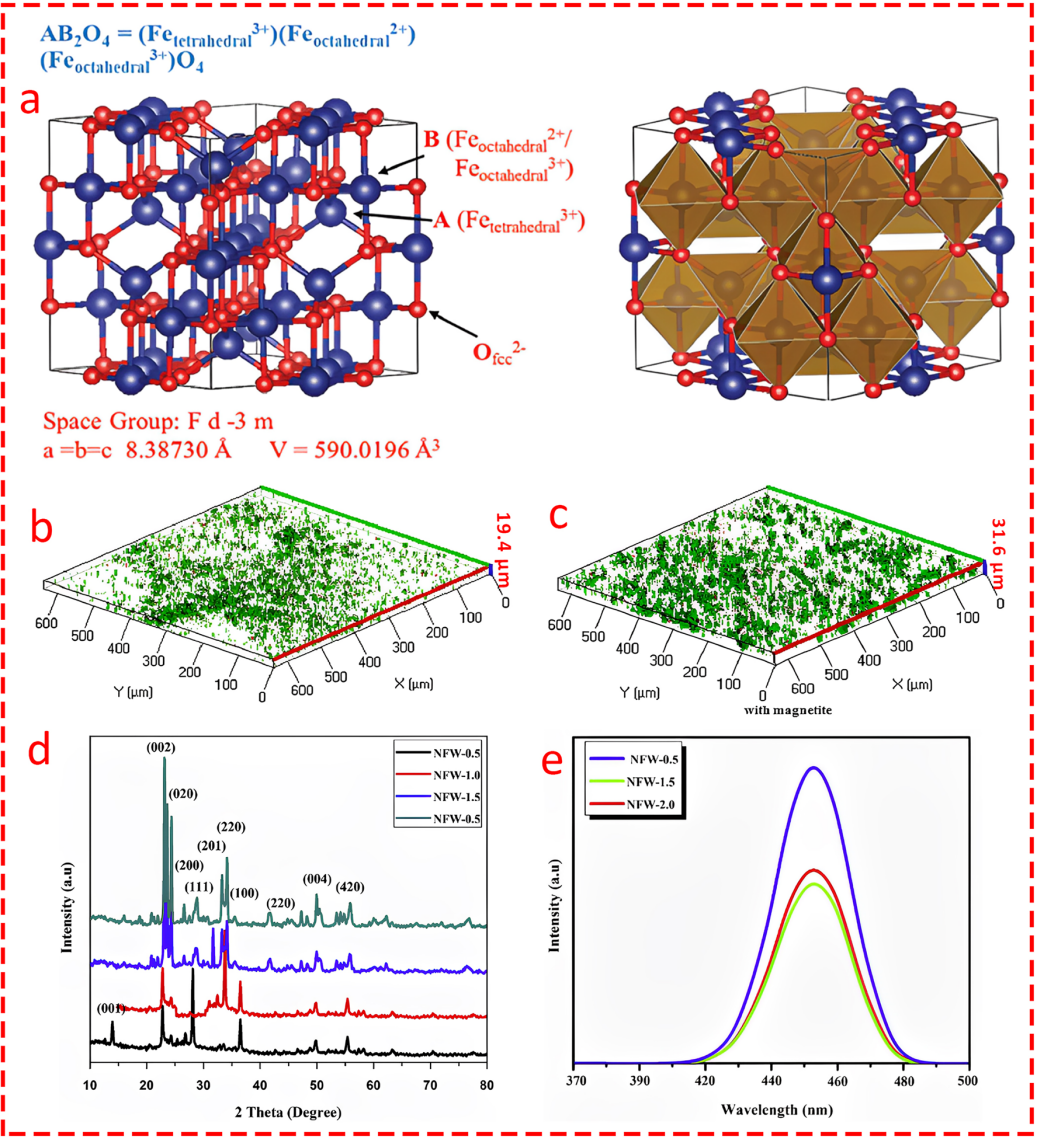
**a** Schematic view of the inverse spinel crystal structure of Fe_3_O_4_ with ball and stick model of the cubic unit cell and polyhedral model of the cubic unit cell. Reprinted from [282] open access source. Accumulation thickness of biofilm on the surface of electrode **b** without Fe_3_O_4_ and **c** with Fe_3_O_4_. Reprinted from [92] with permission from Elsevier. **d** XRD analysis of prepared thin film NiFe_2_O_4_-incorporated WO_3_ nanocomposite (nickel ferrite). Reprinted from [95] with permission from Elsevier. **e** Photoluminescence emission spectroscopy of nanocomposite at various concentrations of nickel ferrite Reprinted from [95] with permission from Elsevier

Transition metal oxides are the most commonly used family of transition metals in electrochemical energy systems due to their stability, abundance, cost, versatility, tunability, just to name a few [85–89]. In the context of MEC, many compositions of TMOs are expressed with the formula A_x_B_3-x_O_4_, referred to as spinel structures; however, other structures such as nanoparticles and amorphous forms are also utilized. It is important to note that although many of the TMO catalysts in MEC follow the spinel structure, the spinel structure is not explicitly mentioned in all studies. However, we concisely discuss each structure and shortly describe the spinel features of each type of catalyst because of the high potential of spinel types in the future design of MEC electrocatalysts. Compared to other metal oxide structures such as layered structures and perovskites, spinel structures have several advantages, like high electrical conductivity, stability in harsh environments, structural defects for high catalytic activity, and high surface area. For instance, mixed valence states (e.g., Fe^2+^/ Fe^3+^ in Fe_3_O_4_) enable rapid electron transfer, boosting electrochemical performance and catalytic activity [90]. Indeed, Fe_3_O_4_ with its inverse spinel structure is one of the most attractive TMOs in a wide range of electrochemical applications. The unique inverse spinel magnetite is a face-centered cubic (fcc) structure where Fe^3+^ occupies tetrahedral sites and Fe^2+^ ions occupy octahedral sites [91] making it highly desirable for electron transfer and catalytic activity (Fig. 8a, b). This makes magnetite a versatile structure that can be utilized through strategies in either pristine or composite forms. Hu et al. [92] fabricated a biocathode electrode modified by Fe_3_O_4_ through co-precipitation methods for the treatment of sulfate-rich wastewater in MEC. The findings revealed that the rate of SO_2_ reduction improved by around 122% compared to a biocathode without magnetite modification. As mentioned above, Fe_3_O_4_ facilitates electron transfer; it was shown that the rate of electron recovery substantially improved. More importantly, Fe_3_O_4_ promoted the formation of biofilm, where the thickness of the biofilm increased from 19.4 to 31.6 µm with and without Fe_3_O_4_, respectively (Fig. 8b, c), indicating an improvement of around 62.8%. This feature of Fe_3_O_4_ demonstrates its significance in scenarios where MECs are assisted with biocathodes. It is also worth pointing out that magnetite nanoparticles coated on the anode of MEC exhibited exceptional improvements, by several folds, in electron transfer facilitation and biofilm enhancement [93, 94]. Tahir et al. [95] synthesized a spinel composite by incorporating nickel ferrite (NiFe_2_O_4_) into a WO_3_ composite catalyst using electrodeposition and spin-coating techniques as a photocathode in MEC integrated with an MFC as the power supplier. The XRD analysis confirmed the formation of monoclinic WO_3_ (Fig. 8d), but the characteristic peaks of NiFe_2_O_4_ were not clearly observed due to its low concentration, shielding effects, or restacking; hence, NiFe_2_O_4_ was not mentioned as spinel. The optimal nickel ferrite concentration from 0.5 wt%–2 wt% was found to be 1.5 wt% (NFW-1.5), where the lowest recombination rate of electron–hole pairs was recorded (Fig. 8e). The NFW-1.5 sample achieved the highest hydrogen production rate of 10.67 mL h^−1^ under visible light irradiation. The MFC generated a stable power output of 300 mV over 25 days of operation, while the modified electrode demonstrated durability and photocatalytic performance for HER compared to pure WO_3_.

**Fig. 9:**
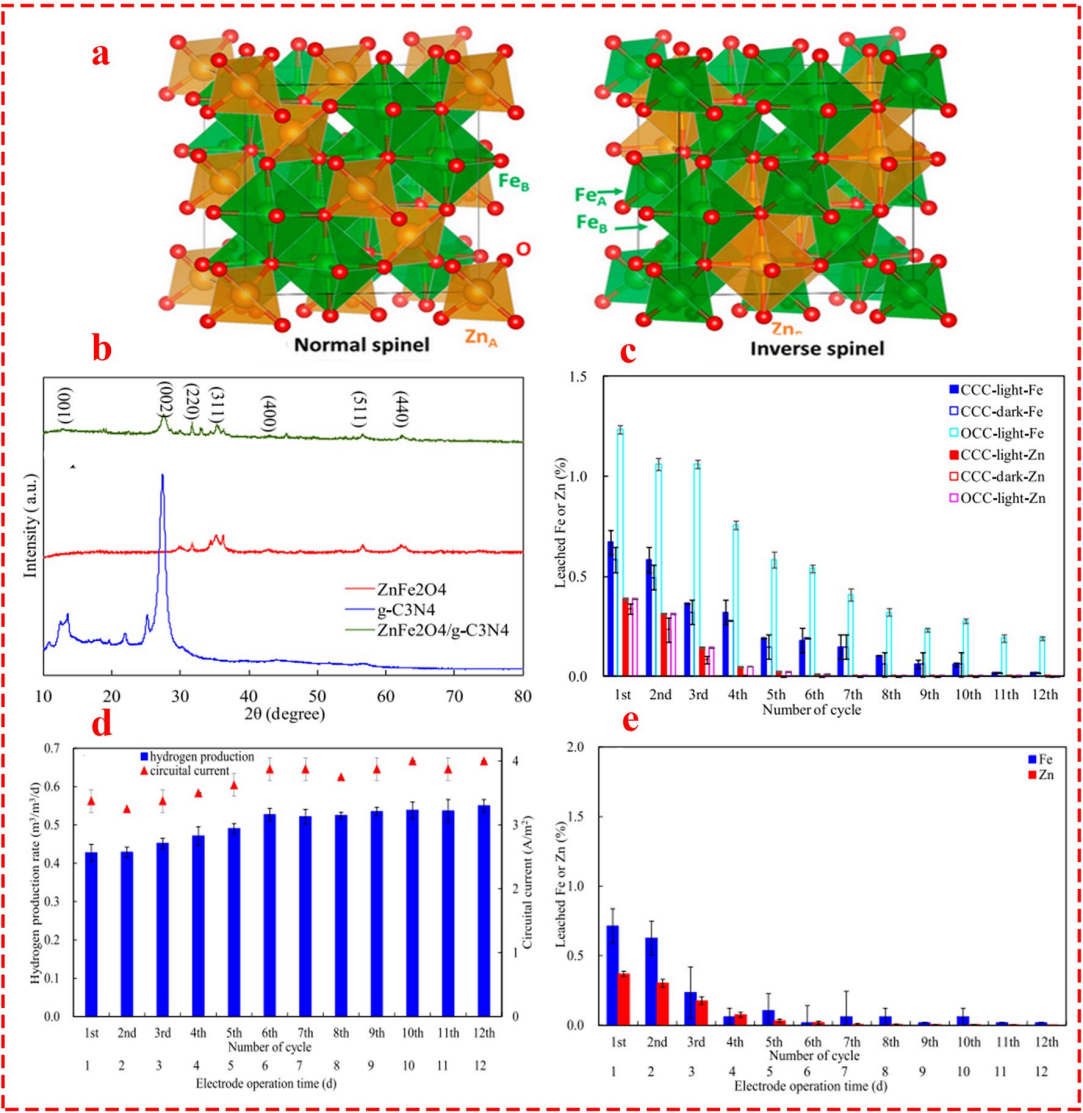
**a** Cubic unit cells of normal and inverse ZnFe_2_O_4_ spinel. Reprinted from [96] open source. **b** XRD patterns of ZnFe_2_O_4_, g-C_3_N_4_, and ZnFe_2_O_4_/g-C_3_N_4_. Reprinted from [98] with permission from Springer. **c** Rate of leaching Fe and Zn in the presence or absence of either light irradiation or circuital current, and as a function of number of batch operation cycles. Reprinted from [98] with permission from Springer. **d** Solar to hydrogen conversion efficiency. Reprinted from [97] open access source. **e** Rate of leaching Fe and Zn in electrolyte during 12 cycles. Reprinted from [97] open access source, Elsevier

Recently, ZnFe_2_O_4_, which has both normal and inverse spinel structures (Fig. 9a), has attracted the attention of researchers as an efficient energy material [96]. Huang et al. [97] fabricated a photo-assisted spinel-type ZnFe_2_O_4_ on the surface of g-C_3_N_4_ for Ni recovery and biohydrogen production in a single-chamber MEC. Electrode characterization was evaluated over 288 h of operation through 12 cycles. Microbial communities on the electrodes and Ni deposition on the cathode after experiments showed improvement in H_2_ production and solar-to-H_2_ efficiency by about 27.9% and 26.4%, respectively, compared to the first cycle (Fig. 9d). From the cost viewpoint, it was realized that even though the structure demonstrates superiority over similar previous studies, the cost (102 $ m^−2^) is more than tenfold lower compared to a well-established composite such as MoS_2_/Cu_2_O (1071 $ m^−2^). Moreover, the nanocomposite exhibited superior durability in terms of metal leaching for both Fe and Zn, in which, after the 5th cycle, the rate of metal leaching was insignificant, particularly for Zn (Fig. 9e). Similarly, Song and co-workers [98] prepared ZnFe_2_O_4_/g-C_3_N_4_ through a facile in situ solid-state calcination as a photocathode in a single-chamber MEC for biohydrogen production. The characteristic peaks in the XRD patterns (Fig. 9b) of ZnFe_2_O_4_ were consistent with the crystal planes of the cubic spinel ZnFe_2_O_4_, exhibiting 35.2° (311), 31.5° (220), 42.3° (400), 57° (511), and 62.4° (440). This study take one step forward than the previous study [97] and examined the rate of electrode corrosion (leaching of metals) during the cycle and elucidated the insignificant decay rate of 0.19% and 0.02% after the 5th cycle for Fe and Zn, respectively (Fig. 9c). However, it is important to note that the type of electrolyte was not mentioned in their study. Interestingly, by comparing these two studies, an important point comes to light. The type of wastewater could have a significant impact on electrode performance. For heavy metal-rich media, metals can act as co-catalysts and increase hydrogen production while inhibiting electrode deposition. Moreover, it appears necessary to examine the electrode under different media (i.e., various types of wastewater) for realistic results.

Regarding the parallel studies conducted by Song et al. [98] and Huang et al. [97], which employed similar spinel structures and performed analogous analyses on metal leaching while reporting similar results on the exceptional reduction rate of metal leaching after initial cycles, it can be concluded that this type of nanocomposite, possibly due to the synthesizing procedure and reaction conditions, the ratio of metal composition, and spinel structure, has high potential for conditions where preventing metal leaching is more important. Moreover, applying further experiments to examine the rate of metal leaching in other well-known structures, such as Ni- and Co-based spinels, could elucidate whether spinels possess the potential to address the problem of metal leaching or not.

**Fig. 10:**
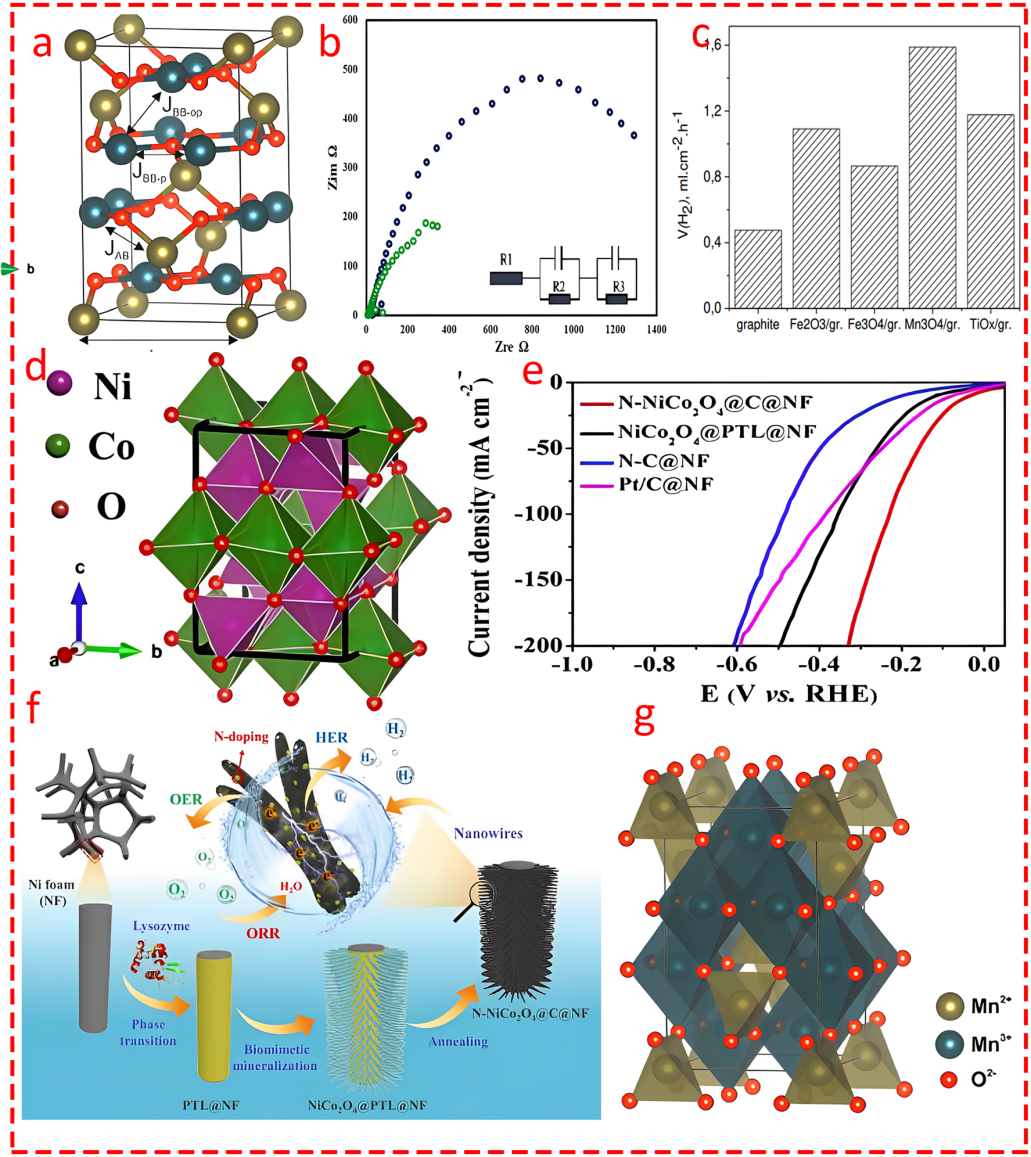
**a** and** g** Unit cell and ball and stick model of Mn_3_O_4_. Blue and green polyhedral represent octahedral [MnO_6_] and tetrahedral [MnO_4_] clusters, respectively. Reprinted from [101] with permission from Elsevier. **b** Nyquist plot of the coated (NM-NF) cathode tested in 50 mM PB solution with standard Ag/AgCl electrode and reference platinum electrode. Reprinted from [112] with permission from Elsevier. **c** Hydrogen evolution rate estimated from the chronoamperograms. Reprinted from [102] open access source. **d** Crystal structure model of NiCo_2_O_4_. Reprinted from [105] with permission from American Chemical Society. **e** Linear sweep voltammetry (LSV) curves of HER. Reprinted from [105] with permission from American Chemical Society. **f** Schematic of the N-NiCo_2_O_4_@C@NF preparation procedure. Reprinted from [105] with permission from American Chemical Society

Mn_3_O_4_ is another normal spinel structure where divalent cations (Mn^2+^) and trivalent cations (Mn^3+^) (Fig. 10a, g) [99–101] are located in the tetrahedral and octahedral coordination, respectively. Chorbadzhiyska and co-workers [102] modified graphite in four different scenarios by coating Fe_2_O_3_, Fe_3_O_4_, Mn_3_O_4_, and TiO_x_ and studied the catalytic activity of electrodes in MEC for biohydrogen production. The chronoamperometry analysis indicated superior reaction kinetics for Mn_3_O_4_ compared to other nanocomposites, particularly Fe_3_O_4_, with the highest and the lowest rate of hydrogen production obtained by Mn_3_O_4_-graphite and pristine graphite, respectively (Fig. 10c). Although the authors did not highlight the reason behind this superiority, one possible mechanism as a reasonable explanation could be that compared to the normal cubic spinel (Fe_3_O_4_), the distorted structure of Mn_3_O_4_ can improve ion mobility and result in higher catalytic activity. Nickel–cobalt oxide (NiCo_2_O_4_) is another spinel structure with broad applications in electrochemical energy storage [103]. Previous research showed the effectiveness of NiCo_2_O_4_ nanoparticles as an additive material for improving the rate of biohydrogen production in the dark fermentation process by promoting the growth of hydrogen-generating microorganisms (Clostridium) and enhancing the expression of pivotal enzymes involved in the hydrogen synthesis pathway [104]. Wu's group [105] fabricated a spinel nanocomposite (Fig. 10d) by growing NiCo_2_O_4_ nanowires on nickel foam, which was further doped with nitrogen atoms through a facile biomimetic mineralization and pyrolysis method (Fig. 10f) as a versatile multifunctional electrocatalyst not only for HER but for ORR/OER. The catalytic activity of the nanocomposite showed superiority in HER compared to other counterparts, particularly Pt/C, with an overpotential of half (42 mV) that of Pt/C (84 mV) catalysts (Fig. 10e). It is worth pointing out that the synergistic effect of the NiCo_2_O_4_ spinel structure, nitrogen doping, and the porous carbon network results in enhanced multifunctional catalytic performance, where the spinel structure provides mixed valence states for efficient redox reactions, nitrogen doping increases electronic conductivity and introduces additional active sites, and the porous carbon structure further boosts mass transport and active site exposure.

**Fig. 11:**
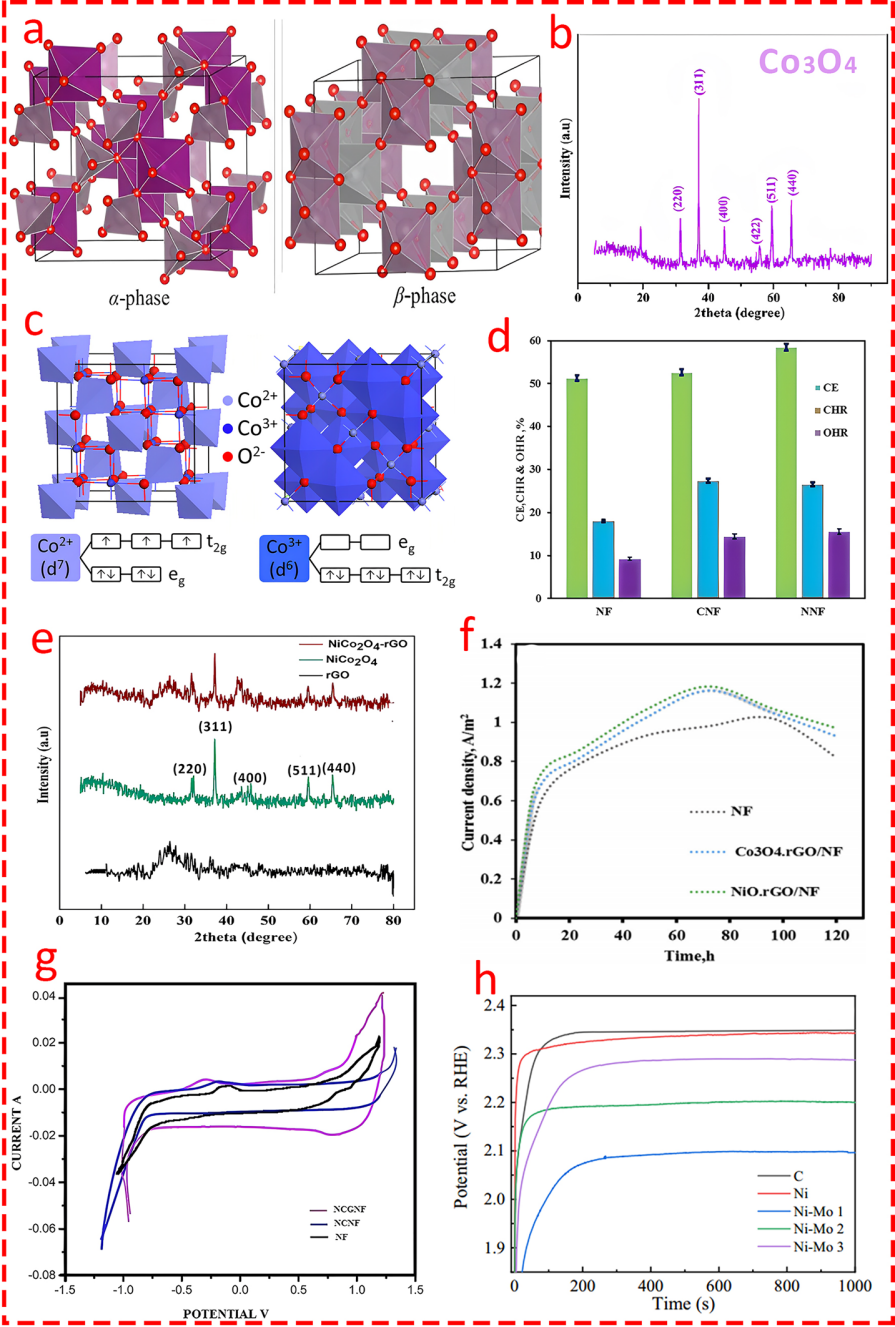
**a** Schematic view of α and β phases of nickel molybdate. Reprinted from [108] with permission from Elsevier. **b** XRD pattern of the Co_3_O_4_ nanocatalyst. Reprinted from [115] with permission from John Wiley and Sons. **c** Structure of Co_3_O_4_ at room temperature which assumes as the normal spinel. Based on the crystal field splitting, the Co^2+^ ions (tetrahedral, light blue) carry magnetic moment, while the Co^3+^ (octahedral, dark blue) ions are nonmagnetic. Reprinted from [113] with permission from American Institute of Physics (AIP). **d** Overall hydrogen recovery (OHR), cathodic hydrogen recovery (CHR), and columbic efficiency (CE) for nickel foam (NF), Co_3_O_4_ coated NF and NiO coated NF. Reprinted from [115] with permission from John Wiley and Sons. **e** XRD pattern of NiCo_2_O_4_.rGO nanocomposite. Reprinted from [106] with permission from Elsevier. **f** Current density of different cathodes in MECs at applied voltage 1.0 V in phosphate buffer solution. Reprinted from [279] with permission from Elsevier. **g** Cyclic voltammogram of NCG-NF, NC-NF and NF electrodes with PB electrolyte. Reprinted from [106] with permission from Elsevier. **h** Chronopotentiometry of different electrodes. Reprinted from [116] with permission from John Wiley and Sons

Jayabalan et al. [106] applied NiCo_2_O_4_ in two scenarios: in its pristine form and decorated on the surface of reduced graphene oxide (rGO) and nickel foam to compare the electrocatalytic performance of the cathode in a double-chamber MEC for enhanced biohydrogen production. The XRD analysis showed similar peaks corresponding to the spinel structure of NiCo_2_O_4_ (Fig. 11e), while electrochemical CV analysis elucidated the modified nanocomposite, with a lower overpotential of 550 mV compared to the composite without rGO and nickel foam with 780 mV (as shown in Fig. 11g). Moreover, in the terms of coulombic efficiency, cathodic hydrogen recovery, and overall hydrogen recovery, the modified cathode showed superior performance over its counterpart. One possible important conclusion is that introducing spinel structures onto the carbon network (whether in the form of porous carbon or rGO) results in superior HER. Indeed, the spinel structure provides abundant active sites and multiple oxidation states for catalytic reactions, while the carbon network improves overall electrical conductivity and supports faster electron transfer.

Nickel molybdate (NiMoO_4_) is another type of spinel structure that is widely employed in electrochemical energy storage schemes, particularly batteries and supercapacitors [107–110] due to its high specific capacitance and good stability. NiMoO_4_ is generally developed in two phases, named α-NiMoO_4_ and β-NiMoO_4_ (Fig. 11a). The main difference between the two phases is that Mo^6+^ ions are present at octahedral sites in the α-phase and at tetrahedral positions in the β-phase [111]. Their stability and electronic features differ, with the β-phase suitable for high-temperature applications and the α-phase favorable for low-temperature applications. Mohamed's group [112] coated NiMoO_4_ nanoparticles on nickel foam as an efficient electrocatalyst for MEC through a facile sonochemical precipitation method. The NM-NF cathode demonstrated superior electrochemical properties, with significantly lower resistance (Fig. 10b), a reduced overpotential of 650 mV compared to 750 mV, and enhanced current density of 8.1 mA cm^−2^ compared to 5.23 mA cm^−2^ for nickel foam. The results indicated that the NiMoO_4_ nanocatalyst increased electron transfer and HER kinetics, achieving a production rate of 0.12 L d^−1^, which was 2.63-fold greater than bare nickel foam, with a 58.2% Coulombic efficiency.

Co_3_O_4_ is a normal spinel based on a cubic close packing [113] (Fig. 11c) array of oxide ions where one-eighth of tetrahedral interstices are occupied by high-spin Co^2+^ (d7) ions and one-half of octahedral interstices are occupied by low-spin Co^3+^ (d6) ions [114]. Jayabalan et al. [115] compared the catalytic activity of two different non-precious metal-based nanocatalyst of NiO and Co_3_O_4_ by coating the catalyst on the surface of nickel foam. The Co_3_O_4_ was prepared through a two-step preparation method using co-precipitation and calcination, and the XRD analysis exhibited peaks at 2θ = 31.2°, 37.3°, 45.8°, 55.2°, 59.3°, and 66.8°, which were ascribed to the (220), (311), (400), (422), (511), and (440) planes (Fig. 11b), respectively, highlighting that the nanocatalyst was spinel. The findings revealed that NiO marginally outperformed Co_3_O_4_ in terms of hydrogen production and coulombic efficiency (Fig. 11d). In a similar study [104], NiO and Co_3_O_4_ were decorated on the surface of rGO and subsequently coated on nickel foam as electrocatalysts for MEC. The electrochemical analysis and system performance were consistent with the above study conducted by Jayabalan et al. [115] where the NiO composite performed slightly better than Co_3_O_4_ in terms of current density (Fig. 11f), coulombic efficiency, cathodic hydrogen recovery, and overall hydrogen recovery.

Although the authors did not highlight the mechanism behind the slightly higher performance of NiO compared to Co_3_O_4_ (in both scenarios, whether decorated on the carbon network or not), one possible explanation could be the simpler redox reaction of Ni^2^⁺ ions compared to Co^2^⁺ and Co^3^⁺ ions, which reduces the overpotential and enhances the HER kinetics for NiO.

In another study, Zhang et al. [116] fabricated NiO/MoO_2_/MoO_3_ via normal pulse voltammetry and used sodium molybdate as a precursor at three concentrations (12.5, 25, and 37.5 g L^−1^, denoted as Ni–Mo1, Ni–Mo2, and Ni–Mo3, correspondingly) as a catalyst for biohydrogen production in MEC. The Ni-Mo-based catalyst exhibited a porous structure with NiO and MoO_x_ as predominant phases. SEM images revealed a fine nanosheet-like structure, particularly in Ni–Mo1, which demonstrated better grain size refinement compared to other samples, with grain sizes calculated as 35.9 nm (Ni–Mo1) compared to 44.2 nm for pure Ni. The XRD patterns confirmed the existence of crystalline NiO and amorphous Mo oxides, with XPS indicating a 2:1 ratio of Mo^4+^ to Mo^6+^. The electrochemical impedance spectroscopy (EIS) data revealed that Ni–Mo1 had the lowest charge transfer resistance (148.72 Ω), correlating with its better electrochemical performance (Fig. 11h) in HER compared to other compositions. The results highlight the effectiveness of Mo addition in improving Ni distribution, surface area, and catalytic efficiency.

**Fig. 12:**
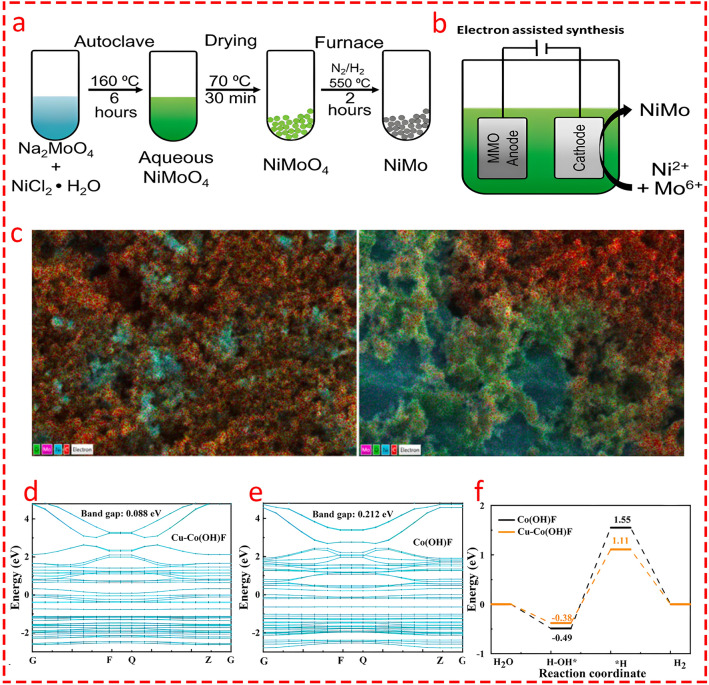
Schematic of the NiMo catalyst synthesis procedures through **a** a hydrothermal synthesis method and **b** an electron-assisted method. Reprinted From [117] with permission from Elsevier. **c** EDS mapping for O, Mo, Ni and C distribution of the NiMo catalyst sprayed on the carbon cloth prepared using the hydrothermal method (Left Side) and the electron assisted method (Right Side). Reprinted From [117] with permission from Elsevier. Energy band gap of **d** Cu-Co(OH)F/NF and **e** Co(OH)F/NF catalysts. Reprinted From [118] with permission from Elsevier. **f** Free energy diagram for hydrogen evolution reaction before and after metal doping. Reprinted From [118] with permission from Elsevier

In an important study, Rossi et al. [117] compared two synthesis methods for NiMoO_4_ catalysts: hydrothermal (NiMo Hth) and electrode-assisted (NiMo Elec) (Fig. 12a, b). Although the authors did not mention the phase of the catalysts, it can be inferred that regarding the hydrothermal method, NiMo Hth was formed in the β-phase and NiMo Elec in the α-phase. NiMo Hth achieved superior results, with a current density of 44.4 ± 0.9 A m^−2^ and a hydrogen production rate of 81 ± 3 L H_2_ L_−1_ d_−1_ at − 0.86 V, while NiMo-Elec showed lower performance with a higher overpotential (120 mV). The superiority of β-phase NiMo Hth could be attributed to its higher density of catalytically active sites, superior electronic conductivity, and better β-phase suitability for hydrogen adsorption energy compared to the α-phase. Interestingly, the NiMo-Hth's better performance was also attributed to its uniform Mo atom distribution as confirmed by EDS and SEM (Fig. 12c), which resulted in enhanced electron transfer and increased catalytic activity. This uniformity was achieved due to the controlled environment of the hydrothermal method, which allowed for better dispersion of Mo atoms compared to the clustering observed in the electrode-assisted method. As a result, the hydrothermal approach led to more active sites and faster hydrogen evolution, contributing to Pt-comparable performance of NiMo-Hth's with a much lower cost, which makes it a viable alternative for large-scale applications.

Liu et al. [118] synthesized a Cu-doped Co(OH)F nanowire catalyst on nickel foam using a one-step hydrothermal method to enhance hydrogen production in MECs. The DFT calculations revealed that Cu doping significantly reduced the band gap, enhancing the catalyst’s electronic conductivity by increasing electron density at the Fermi level. The Gibbs free energy diagram (Fig. 12d, e) showed that Cu incorporation lowered the energy barrier for HER, optimizing reaction kinetics by decreasing the required energy for hydrogen adsorption and desorption (Fig. 12f). The Cu-Co(OH)F/NF catalyst demonstrated a higher hydrogen production rate (214.5 mL H_2_/L/cycle) and improved hydrogen recovery efficiency (26.76%). These findings establish Cu-Co(OH)F/NF as an efficient, low-cost catalyst for hydrogen evolution in MECs.

### Transition Metal Dichalcogenides

**Fig. 13:**
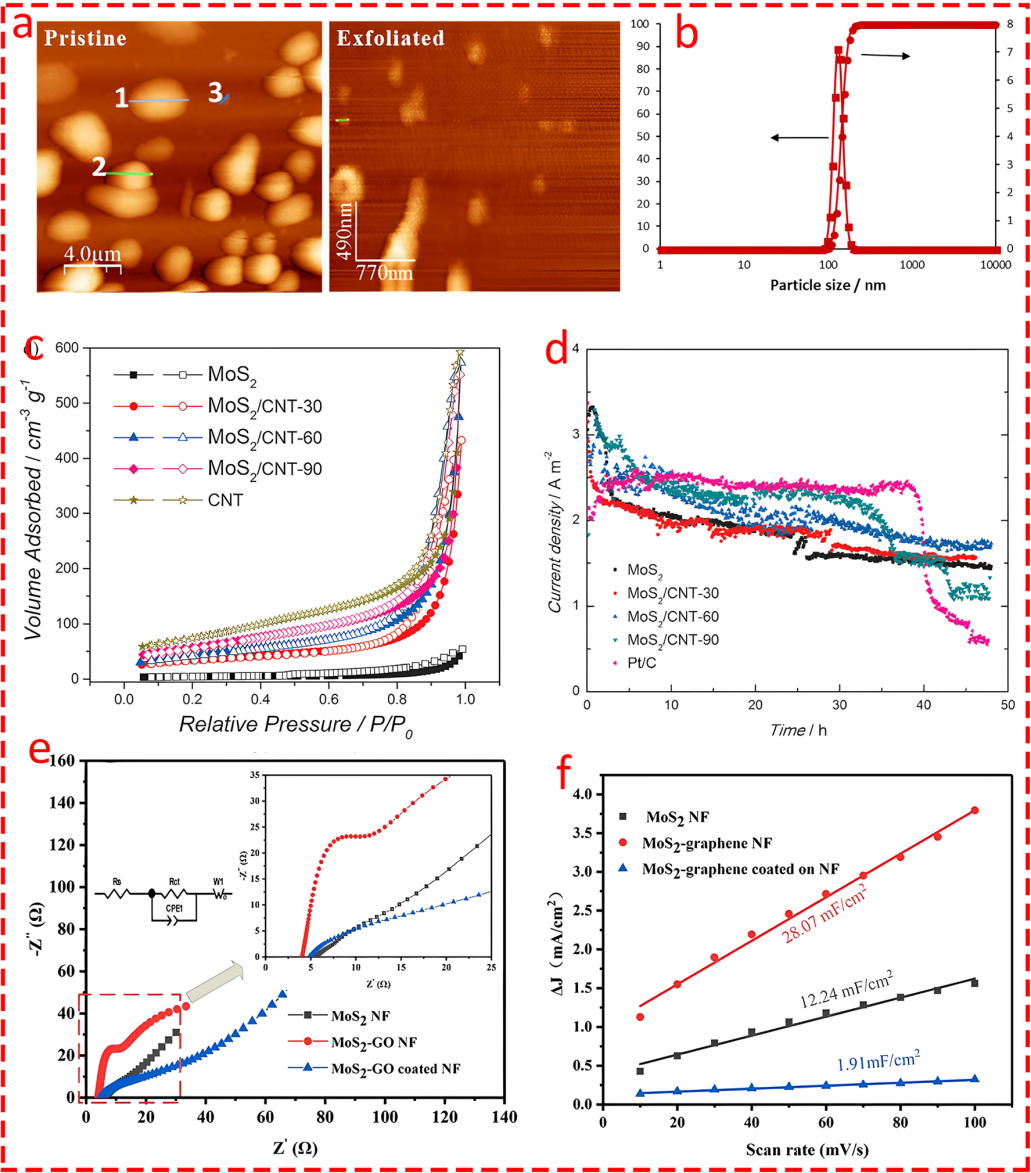
**a** Atomic force microscopy (AFM) topography images for pristine MoS_2_ and Exfoliated MoS_2_. Reprinted from [120] with permission from Elsevier. **b** Dynamic light scattering (DLS) analysis of the synthesized exfoliated MoS_2_ catalyst. Reprinted from [120] with permission from Elsevier. **c** Nitrogen adsorption–desorption isotherms. Reprinted from [122] with permission from John Wiley and Sons. **d** Current density of the MEC with MoS_2_ at different fractions with CNT or Pt at a constant applied voltage. Reprinted from [122] with permission from John Wiley and Sons. **e** and **f** Electrochemical impedance spectroscopy plots of MoS_2_ NF, MoS_2_-GO NF and MoS_2_-GO coated NF elucidating electrode resistance and electrical double-layer capacitance, respectively. Reprinted from [123] with permission from Elsevier

Transition metal dichalcogenides are another important category of transition metals utilized as catalysts in MECs. Interestingly, MoS_2_ stands at the top of TMDs due to its unique features, such as high surface area, low bandgap, high electrical conductivity, catalytic activity, and mechanical strength, all of which play an important role when used as an electrode in MECs. In the early stages of MEC, MoS_2_ was used in general commercial form without any modifications [119]. However, later studies showed that modifying its morphology can substantially improve the catalytic activity of MoS_2_. For instance, Rozenfeld et al. [120] compared the catalytic activity of exfoliated MoS_2_ with its pristine form as catalysts in single-chamber MECs. The dynamic light scattering (DLS) analysis exhibited that the exfoliated MoS_2_ particles were significantly smaller, with an average size of 200 ± 50 nm, compared to pristine MoS_2_ particles (Fig. 13b), leading to an increase in the active surface area. Moreover, AFM topography images highlighted the smaller, more uniform particles of exfoliated MoS_2_, resulting in a lower height profile and more exposed active sites (Fig. 13a). This structural improvement led to enhanced catalytic activity, where the exfoliated MoS_2_ electrode demonstrated improved current density performance, achieving 50.68 mA cm^−2^ at − 1.3 V, compared to 3.09 mA cm^−2^ for pristine MoS_2_, making it the optimal candidate for MEC applications. One of the main advantages of utilizing MoS_2_ over well-known catalysts like Pt is its lower cost; however, finding the optimum value is an important factor.

**Fig. 14:**
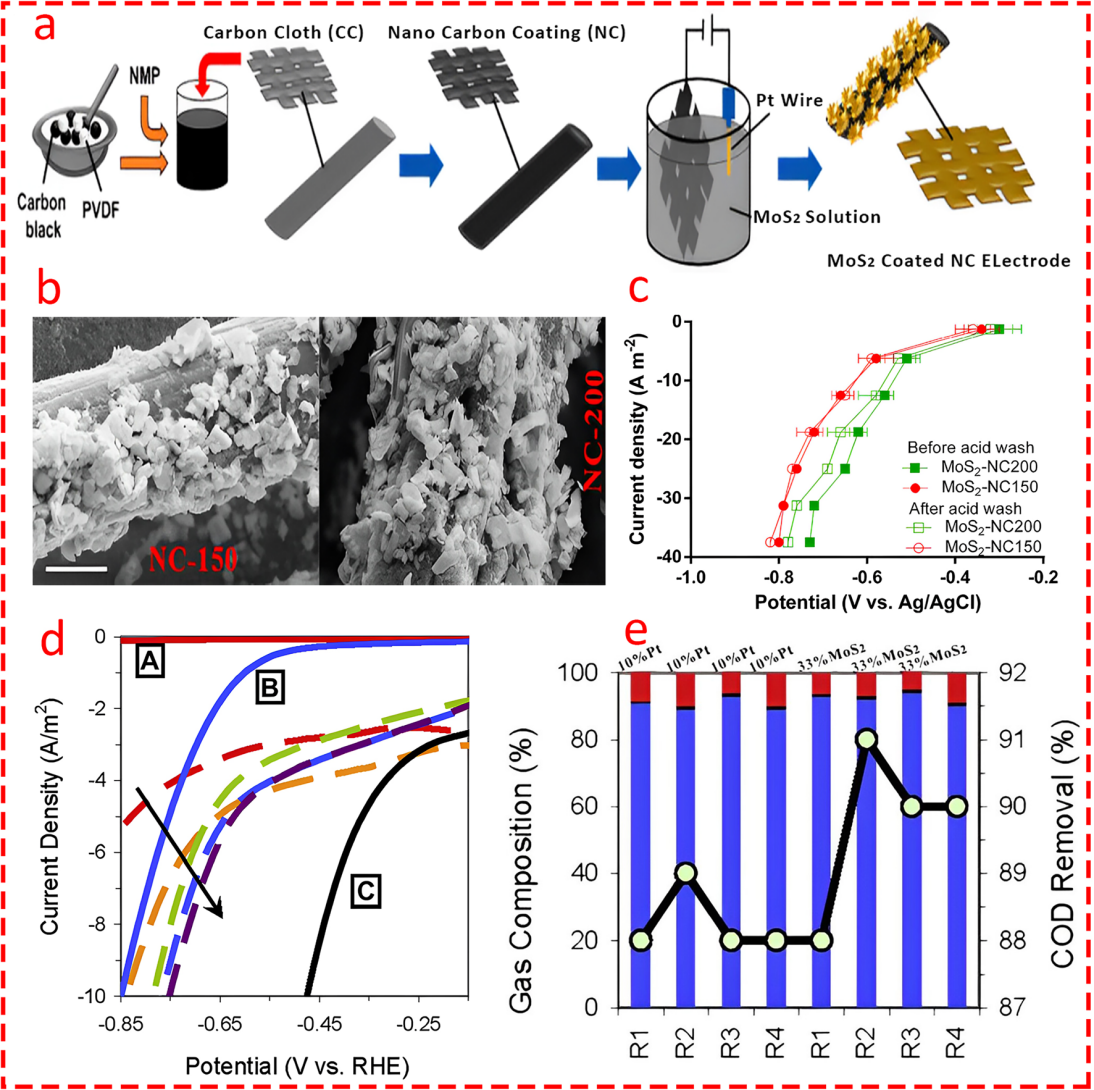
**a** and **b** Schematic of the MoS_2_-NC electrode fabrication procedure and SEM image of MoS_2_ decoarted on the surface of electrode at two currents applied of 150 and 200 μA cm^−2^ through electrodeposition. Reprinted from [126] with permission from Elsevier. **c** Analysis of electrochemical reduction of electrodes before and after acid washing. Error bars indicate mean ± standard deviation (n = 3). Reprinted from [126] with permission from Elsevier. **d** LSV scans of selected cathodes in 0.1 M NaClO_4_ are shown as follows: solid lines represent (A, red) bare stainless steel, (B, blue) MoS_2_-coated stainless steel, and (C, black) 10% platinum on carbon cloth. The dashed lines correspond to different MoS_2_ loading ratios added to carbon black and applied to carbon cloth: 0 wt% MoS_2_ (1, red), 9 wt% MoS_2_ (2, orange), 23 wt% MoS_2_ (3, green), 33 wt% MoS_2_ (4, blue), and 47 wt% MoS_2_ (5, violet). The arrow indicates increasing MoS_2_ loading ratios for the dashed lines. Reprinted from [121] with permission from Elsevier. **e** MEC gas composition (stacked bars, left axis) and percent COD removal (black dots, right axis) are presented as follows: Gas composition includes H_2_ (blue, bottom), CH_4_ (black, middle), and CO_2_ (red, top). Separate results for gas composition and percent COD removal are shown as averages over six batch cycles for MEC reactors using 10 wt% Pt cathodes and over three batch cycles for those using MoS_2_ cathodes. Reprinted from [121] with permission from Elsevier

For instance, Logan's group [121] synthesized MoS_2_ with four loading ratios (9, 23, 33, and 47 wt%) on carbon black to evaluate its catalytic performance in MECs and sought to find the optimum catalyst ratio. The findings revealed that although a loading ratio of 47 wt% yielded better results, the difference was marginal compared to 33 wt% (less than 2%) (Fig. 14d). Hence, the optimum concentration of MoS_2_ was determined to be 33 wt%, as it balanced performance and cost. Moreover, the MoS_2_-33 with a surface density of 25 g m^−2^ offered lower overpotential and enhanced HER efficiency compared to platinum-based catalysts while maintaining high stability in hydrogen production and COD removal (Fig. 14e). In another study [122], MoS_2_/CNT nanocomposites were synthesized using a hydrothermal method as a high-performance catalyst in MECs. Various concentrations of CNT (30, 60, and 90 wt%) were evaluated to enhance conductivity, and it was shown that MoS_2_/CNT-90 provided the highest surface area and pore volume (Fig. 13c), enhancing the number of exposed active sites for hydrogen evolution. Although MoS_2_/CNT-60 and MoS_2_/CNT-30 exhibited moderate performance, MoS_2_/CNT-90 achieved the highest current density and hydrogen production rate (QH_2_ ≈ 0.0101 m^3^ H_2_/m^2^/d), closely approaching that of the Pt-based electrode (Fig. 13d). Thus, the MoS_2_/CNT-90 as the optimal candidate was selected due to its balance of surface area, conductivity, and catalytic efficiency. Hou et al. [123] coated MoS_2_-graphene oxide (MoS_2_-GO) on the surface of NF as a catalyst in a comparative study with MoS_2_-NF. Their findings revealed that the MoS_2_-GO-NF cathode achieved the highest electrical double-layer capacitance (C_dl_ = 28.07 mF cm^−2^), indicating a larger electrochemically active surface area compared to MoS_2_-NF (Fig. 13f). Moreover, the EIS analysis revealed that MoS_2_-GO-NF had the lowest charge transfer resistance (R_ct_ = 4.84 Ω) (Fig. 13e), demonstrating superior conductivity and electron transfer efficiency, resulting in the highest HER performance.

Moreover, combining other semiconductors like Cu_2_O in various scenarios can boost electrode performance in different ways. For example, by coating MoS_2_ on Cu_2_O in a photocathode-assisted MEC, the rate of hydrogen evolution substantially increased as a result of high surface active sites, which led to a high reduction in proton activity [124]. Furthermore, in another scenario, Cu_2_O acted as a bridge between two semiconductors—MoS_2_ and rGO—with a double-purpose approach in order to enable efficient charge transfer between them while maintaining structural stability by uniformly dispersing MoS₂ on the surface of rGO and inhibiting clustering. Dai and co-workers experimentally validated this by combining Cu with MoS_2_ and rGO (MoS_2_-Cu-rGO) as a bridge. The catalyst achieved the lowest Tafel slope compared to MoS_2_-rGO and Pt-C (Fig. 13e) [125]. As a result, it can be concluded that increasing active sites for higher catalytic activity, aided by materials with good conductivity (a well-established example being graphene oxide), can be considered a strategy to enhance the HER [123]. Hence, carbon-based materials in various forms seem to play a vital role in developing high-performance electrodes. Hwang et al. prepared MoS_2_/NC-CC electrocatalyst through a facile two-step process involving nano carbon coating on carbon cloth and subsequent electrodeposition of MoS_2_ nanoparticles on the NC (Fig. 14a) for biological hydrogen production in a double-chamber MEC from human urine. Importantly, the electrodeposition process was applied at two different currents (150 and 200), and it was found that a higher density of nanoparticles was dispersed at the higher applied current (Fig. 14b). The use of nanocarbon offers the advantage of reducing costs compared to previously mentioned carbon derivatives, while providing a higher surface area than most counterparts. More importantly, they examined the stability of the electrode by acid washing (both MoS_2_/NC-CC-150 and MoS_2_/NC-CC-200) after three cycles of MEC. The results showed that only a very small portion of MoS_2_ nanoparticles (2%) were lost due to acid washing. However, the catalytic activity of both electrodes remained stable with a very insignificant reduction in catalytic activity (Fig. 14c), highlighting the durability of the MoS_2_/NC-CC. Moreover, it was found that 1 kg of hydrogen production requires 1600 kg of human urine [126].

**Fig. 15:**
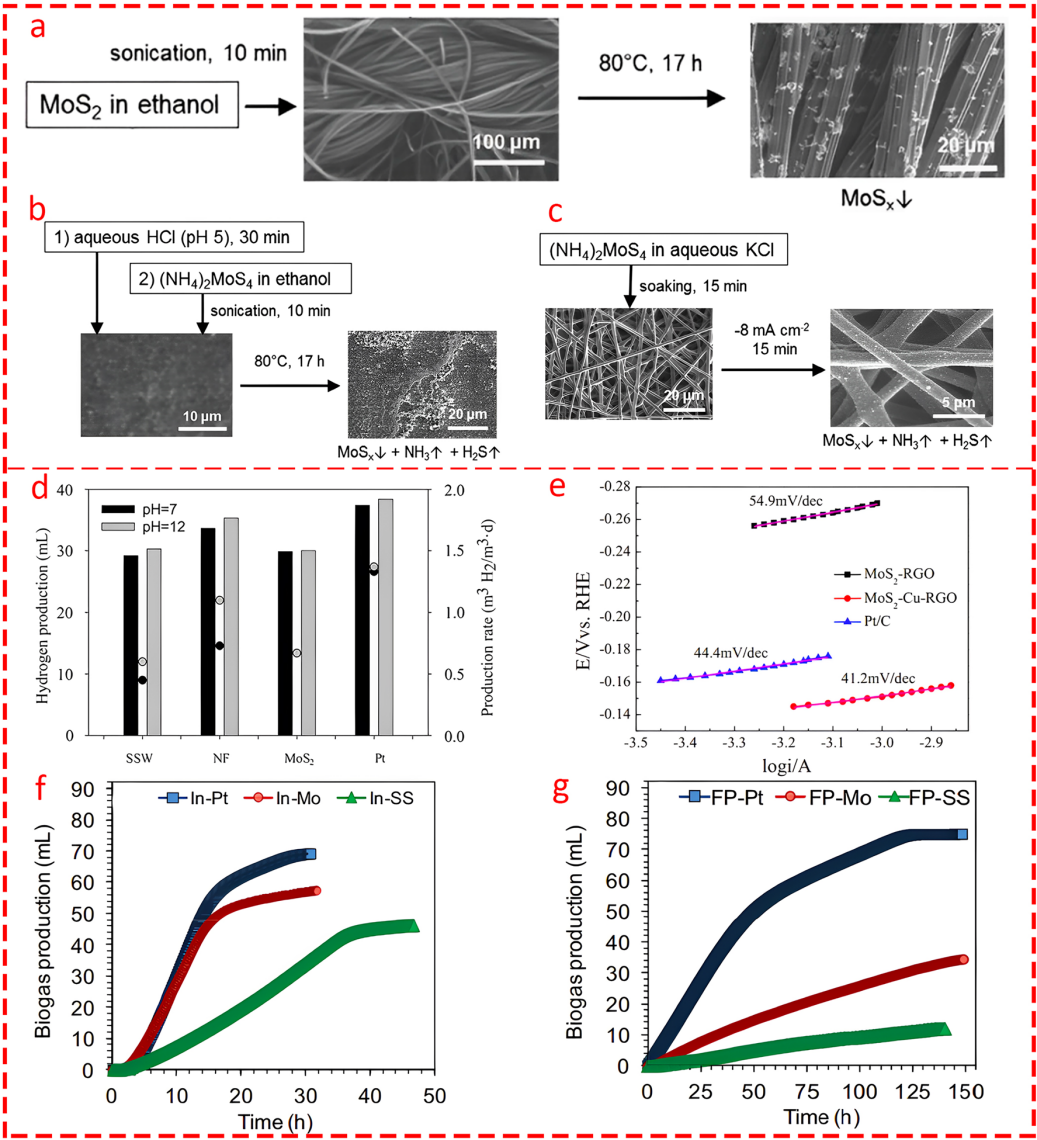
A schematic illustration of MoS_x_ deposition is shown as follows: **a** on activated carbon cloth (ACC) using drop coating (DC), **b** on a buckypaper (BP) electrode using impregnation followed by heat treatment (IHT), and **c** on electrospun (ES) carbon nanofibers using electrodeposition (ED). Reprinted from [127] with permission from Elsevier. **d** Hydrogen production (bars) and the corresponding production rate (circles) are shown for each material at varying initial cathode pH levels. Reprinted from [128] with permission from Elsevier. **e** Tafel plots for MoS_2_-RGO, MoS_2_-Cu-RGO, and Pt/C electrodes. Reprinted from [125] with permission from Elsevier. Biogas production for different electrodes in MECs with platinum (Pt), molybdenum disulfide (Mo) and stainless steel mesh (SS) cathodes fed by **f** industrial (IN) wastewater and **g** for food processing wastewater. Reprinted from [129] with permission from Elsevier

Kokko et al. [127] focused on the role of different synthesizing methods for MoS_x_ fabrication on various carbon supports to enhance HER in MECs. Three synthesizing methods—drop coating, impregnation with heat treatment, and electrodeposition (Fig. 15a–c)—were evaluated. Among the tested cathodes, MoS_x_ electrodeposited on buckypaper (BP-ED) exhibited the best performance, with an overpotential of 100 mV in acidic industrial wastewater which closely rivaling platinum. Electrodeposition proved superior by creating smaller, well-dispersed MoS_x_ particles, enhancing surface area and catalytic activity. Notably, BP-ED achieved higher hydrogen production rates (0.39 m^3^/m^3^/d) than the other MoS_x_ electrodes. The main outcome was that MoS_x_ electrodes, particularly BP-ED, demonstrated improved catalytic efficiency over time in acidic wastewater, even outperforming platinum due to structural changes that enhanced long-term performance.

However, some researchers have shown that MoS_2_'s superiority is context dependent. In comparative studies, the performance of MoS_2_ electrocatalysts with other well-known electrodes, including stainless steel, nickel foam, and Pt/C, showed higher overpotential and lower hydrogen production in neutral and alkaline pH environments (Fig. 15d) [128].

Tenca and co-workers [129] compared the performance of MoS_2_ electrocatalysts and stainless steel as two low-cost alternatives to Pt/C catalysts for two different types of wastewater: industrial and food processed. Their findings indicated that MoS_2_ outperformed SS in catalytic activity but delivered less biogas and current than Pt catalysts. Interestingly, they found that the type of wastewater significantly impacts the operation time of catalysts. For SS, the catalyst exhibited a significantly longer operation time for industrial wastewater, while for food-processed wastewater, MoS_2_ demonstrated a slightly longer operation time (Fig. 15f, g).

### Transition Metal Phosphides

**Fig. 16:**
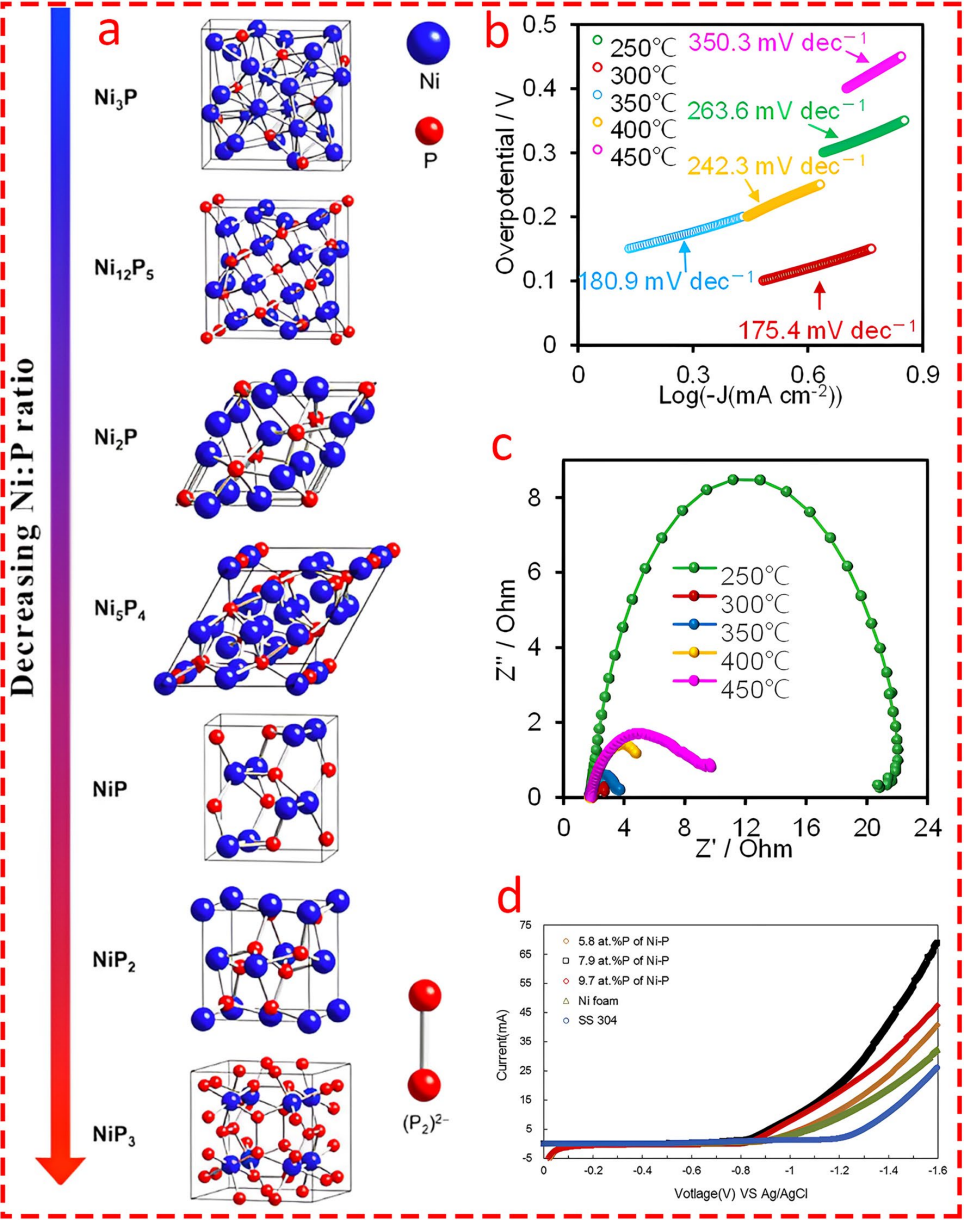
**a** Crystal structures of various nickel phosphides spanning a range of Ni:P ratios. The (P_2_)^2−^ dimer, which appears in the NiP_2_ and NiP_3_ polyphosphides, is also shown. Reprinted from [133] with permission from American Chemical Society. **b** Tafel plots of CoP-NF at different phosphating temperatures in 1 M PBS solution. Reprinted from [135] with permission from Elsevier. **c** EIS spectra of CoP-NF at different phosphating temperatures in 1 M PBS. Reprinted from [135] with permission from Elsevier. **d** LSV curves of cathodes at different P content over a potential range of 0 to −1.6 V at a scan rate of 1 mV s^−1^ in practical MEC reactors filled with pH 11 buffer solution. Reprinted from [138] with permission from Elsevier

Nickel and cobalt phosphides are among the most widely studied transition metal phosphides in various electrochemical energy applications [130–132]. The Ni–P ratio plays a crucial role in determining the catalytic performance of nickel phosphides in HER. As the phosphorus content increases, Ni − Ni interactions decrease while Ni − P coordination increases which significantly alters the material’s structure and electronic properties [133]. Phosphides characterized by a metal-rich composition typically exhibit enhanced electrical conductivity and corrosion resistance due to the abundance of metal bonds present. Conversely, composites with a phosphorus-rich structure have a higher concentration of P-P bonds and frequently demonstrate significant catalytic activity [134]. Indeed, phosphorus-rich phases, such as NiP_2_, exhibit superior catalytic activity compared to metal-rich phases like Ni_2_P, due to a higher positive charge on Ni atoms and a favorable ensemble effect from phosphorus. These structural changes, as illustrated in Fig. 16a, enhance the exposure of active sites, promoting better catalytic efficiency and stability in HER. Thus, tuning the Ni–P ratio is key to optimizing transition metal phosphides for electrocatalytic applications.

**Fig. 17:**
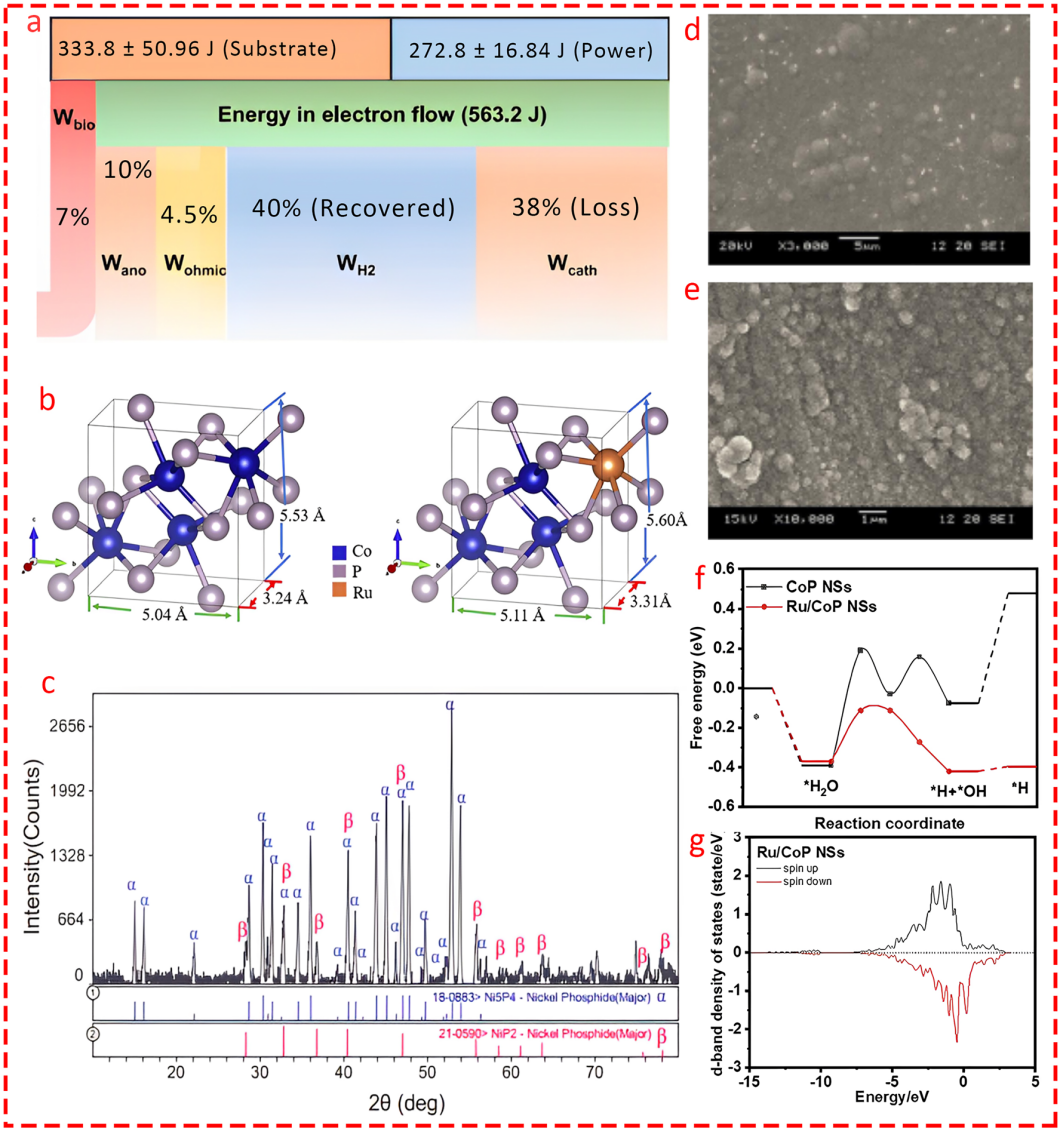
**a** Energy flow analysis in CoP-NF MEC under an applied voltage of 0.7 V. Input energy consists of substrate consumption (W_S_) and electrical input (W_E_). Output energy includes hydrogen recovery (WH_2_), energy loss from non-anode respiring reactions (W_bio_) associated with non-electroactive biofilm metabolism, anode potential loss (W_ano_) related to biofilm metabolism and pH gradient, energy loss due to internal ohmic resistance (W_ohmic_) linked to electrolyte conductivity and membrane resistance, and cathode side reactions and potential loss (W_cath_) associated with cathode catalysts, side reactions, and pH gradient. Reprinted from [135] with permission from Elsevier. **b** Crystal models of CoP NSs and Ru/CoP NSs with lattice constants [137] with permission from Royal Society of Chemistry. **c** XRD pattern of both Ni_5_P_4_ and NiP_2_ showed the well-balance distribution of the combined nanocomposite. Reprinted from [140] with permission from Elsevier. SEM images of **d** Ni-W–P nanocomposite (at 10,000 magnification) [142]. **e** Ni-Ce-P nanocomposite (at 3,000 magnification). Reprinted from [142] with permission from International Water Association. **f** Free energy diagram for the HER on CoP nanosheets and Ru/CoP nanosheets. Reprinted from [137] with permission from Royal Society of Chemistry. **g** State density maps of the adsorption active sites of Ru/CoP nanosheets. Reprinted from [137] with permission from Royal Society of Chemistry

In an important study, Linag et al. [135] fabricated a cobalt phosphide catalyst, which was subsequently coated on the surface of nickel foam in a double-chamber MEC for biohydrogen production through a facile two-step preparation method involving hydrothermal treatment and phosphization at various temperatures. They highlighted the pivotal role of phosphidation temperature and prepared the catalyst in the temperature range of 250–450 °C, with the lowest overpotential observed at 300 °C (Fig. 16b). The CoP-300-NF cathode, by delivering the highest current density across a wide range of applied voltages and exhibiting several-fold lower internal resistance compared to other prepared catalysts (Fig. 16c), outperformed Pt/C commercial catalysts and achieved the highest HER. Moreover, the energy balance flow (Fig. 17a) showed that of the total energy input to the MEC from the substrate and external voltage (563.2 J), 40% was recovered. Importantly, the lowest energy loss (4.5%) was attributed to the electrode’s low resistivity, while the highest loss (38% of the total energy flow) occurred in the cathode chamber, including losses from the pH gradient and side reactions. This highlights the cathode side as a vital component for the practical application of MECs.

Hagos et al. [136] prepared cobalt phosphorus catalysts and coated them on the surface of two stainless steel meshes (grades 80 and 200)-316 through an electrodeposition method in a single-chamber MEC integrated with anaerobic digestion (AD) to promote methane production in AD through endogenous hydrogen generation in MEC. The findings revealed that the SS-200 mesh produced marginally more hydrogen, around 4.5% (152.7 vs. 146.12 mL), than SS-80, leading to nearly 48% higher methane production compared to conventional AD. One possible interpretation for the slight superiority of the SS-CoP-200 cathode over SS-CoP-80 is that the finer surface of the cathode provides higher surface area which facilitating better microbial growth and higher charge transfer.

In an interesting study, Dai et al. [137] doped ruthenium (Ru) into cobalt phosphorus nanosheets (Ru/CoP) (Fig. 17b) to enhance HER in MECs by improving both hydrogen adsorption and desorption processes. The Ru/CoP sheets were synthesized through a two-step process involving co-precipitation and phosphating at 350 °C. The nanosheets exhibited a high surface area and exposed more active sites. To understand the energy barriers and HER mechanism of the novel catalysts, the intermediate stable configurations for H_2_O, H + OH, and H were assessed using DFT calculations. It was shown that Ru doping enhances catalytic performance by lowering the energy barrier for water dissociation and shifting the d-band center of CoP (Fig. 17f, g). This shift reduced hydrogen adsorption energy, facilitating easier hydrogen desorption and improving overall hydrogen evolution efficiency. Moreover, Ru/CoP outperformed Pt/C in several metrics, including a lower overpotential (38 mV at 10 mA cm^−2^ in alkaline conditions) and a higher hydrogen production rate (0.1434 m^3^/m^2^/d).

Li et al. [138] prepared Ni–P through chemical plating and controlled parameters such as bath temperature and plating time to examine the effect of phosphorus content as the cathode of MEC. The P content played a crucial role in the coating’s structure, where a P content below 4% resulted in a crystalline structure, but above 12% led to an amorphous structure, both of which reduced hydrogen evolution performance. They concluded that the optimal P content lies between 4 and 12%, with 7.9% striking a balance between crystalline and amorphous phases, resulting in a significantly lower overpotential of − 0.85 V and the highest current density (Fig. 16d). The MEC with the Ni–P cathode achieved a hydrogen production rate of 2.29 ± 0.11 L H_2_/L/d, 7.5% higher than Ni foam and 110% higher than stainless steel with improved methane inhibition due to better hydrogen recovery. In another study [139], hexagonal Ni_2_P nanoparticles were synthesized using a solution-phase method and dispersed on carbon black to create Ni_2_P/C catalysts in MEC. The modified cathode exhibited a significantly higher mass-normalized current density, approximately 14 times greater than Ni/C, and achieved similar hydrogen production using significantly less catalyst mass (0.5 mg cm^−2^ Ni_2_P vs. 6 mg cm^−2^ Ni in Ni/C). The optimal hydrogen production was attributed to the P heteroatom's ability to reduce hydrogen bonding energy and attract protons, facilitating HER kinetics.

Cai et al. [140] fabricated a Ni_5_P_4_-NiP_2_ mixed matrix nanosheet through a one-step phosphorization method at 500 °C as a cathodic catalyst in MEC for hydrogen recovery. NiP_2_, which exhibits among the highest HER activity compared to most non-noble metal electrocatalysts [141], is more phosphorus-rich than Ni_5_P_4_ and provides a higher number of exposed phosphorus sites, which are critical for enhancing HER. These phosphorus sites are effective in reducing hydrogen binding energy, facilitating both hydrogen adsorption and desorption during the reaction. The well-distributed contents of metal-rich Ni_5_P_4_ and P-rich NiP_2_ exhibited strong intensities (Fig. 17c) and enhanced overall electron transfer kinetics through their balanced combination. The Ni_5_P_4_ features longer Ni–P bonds compared to Ni_2_P, making the initial electron transfer for HER more favorable, while NiP_2_ increasing the number of active sites and expanding the surface area, contributing to improved catalytic performance.

Wang et al. [142] prepared two bimetallic phosphorus-containing catalysts, Ni/W–P and Ni/Ce-P, via a facile electrodeposition method to compare their catalytic activity. The Ni-W–P cathode achieved a 55.7% higher hydrogen production rate (1.09 m^3^/m^3^/day) compared to Ni-Ce-P and performed slightly better than Pt (1.03 m^3^/m^3^/day). The Ni-W–P cathode also delivered coulombic efficiency, cathodic hydrogen recovery, and energy efficiency values of approximately 56%, 74%, and 139%, respectively. The superior performance of Ni/W–P over Ni/Ce-P was attributed to its higher intrinsic activity through the synergies between Ni, W, and P, a larger effective surface area, and smaller particle size (around 1 µm for Ni-W–P compared to 2.5 µm for Ni-Ce-P) (Fig. 17d, e), which provided more active sites for HER. Moreover, previous studies have highlighted that the tungsten composition with nickel improves the electronic structure and charge transfer of Ni, delivering high efficiency for HER [143–145], while cerium, which is less commonly employed for HER compared to tungsten, is more favorable for reactions where oxygen is central, such as ORR and OER [146, 147]. Mondal's research group investigated the utilization of Ni, Ni-Co, and Ni-Co-P nanocatalysts prepared via electrodeposition on stainless steel (SS316) and copper (Cu) substrates as cathodes in MECs. The catalysts were electroplated under controlled conditions, creating coatings with high surface areas and improved stability. Among the tested configurations, the Ni-Co-P catalyst on SS316 (SS4) achieved the highest hydrogen production rate of 0.16 m^3^/m^3^/d at 0.6 V and outperformed both the Ni and Ni-Co coatings. Moreover, the SS-based cathodes demonstrated better performance compared to Cu, likely due to their enhanced electron and proton transfer efficiency and strong synergies between catalysts and substrate, with Ni-Co-P on SS316 achieving up to 77% higher hydrogen yield than bare electrodes. It is worth noting that the ensemble effect of Ni and P atoms significantly improves catalytic activity. The balance between Ni_5_P_4_, with its stable metallic phase, and the P-rich NiP_2_ results in enhanced electron conductivity and catalytic activity compared to either phase alone [148].

### Transition Metal Carbides

**Fig. 18:**
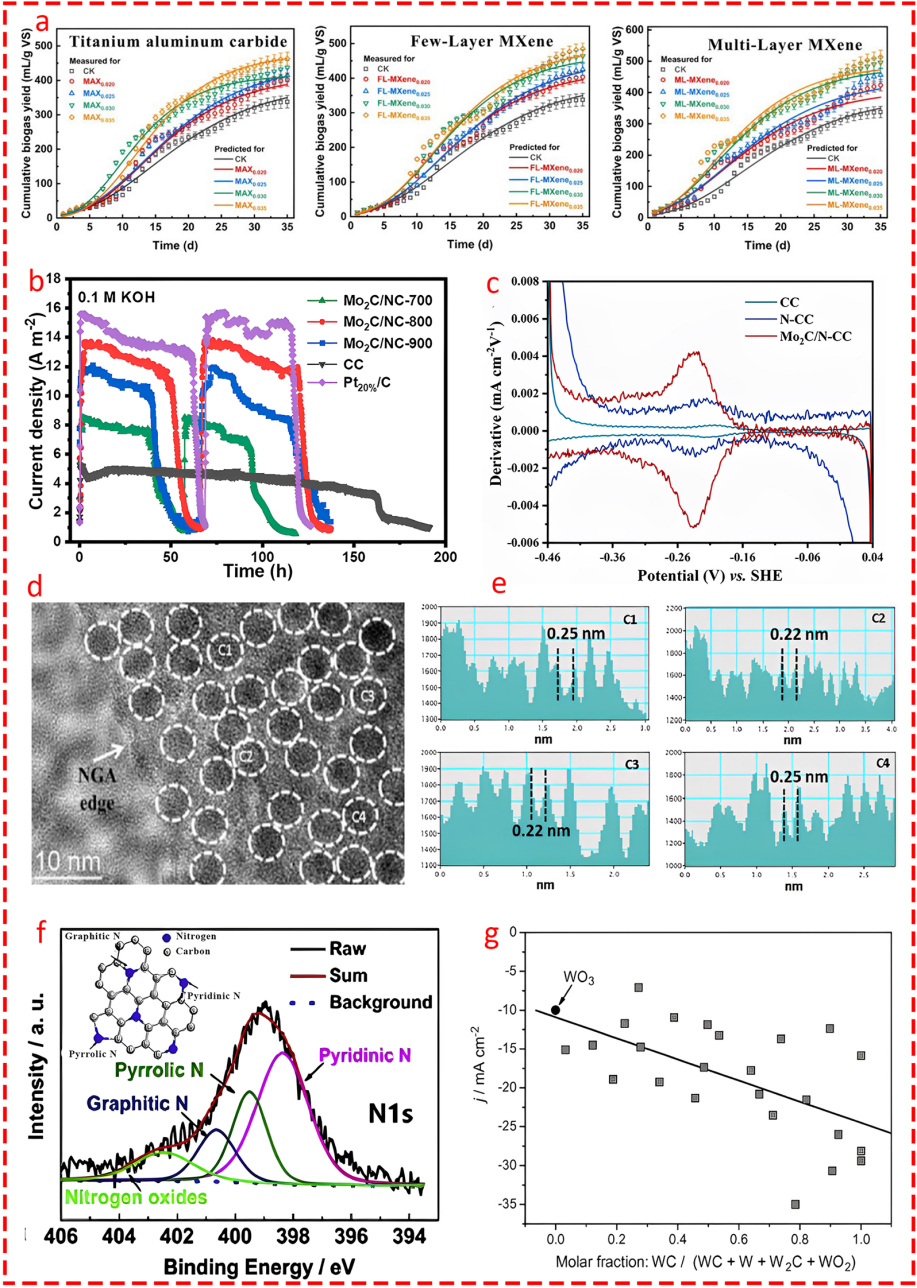
**a** Measured and fitted accumulated biogas yields of the system after adding different dosages of (from right to left) titanium aluminum carbide, few-layered MXene and multilayered MXene. Reprinted from [161] with permission from Elsevier. **b** Current density diagram of Mo_2_C/NC prepared at three different from 700–900 °C denoted as Mo_2_C/NC-700, Mo_2_C/NC-800, Mo_2_C/NC-900, CC, and Pt 20%/C cathode in MECs with 0.1 M KOH solution. Reprinted from [154] with permission from Elsevier. **c** Enhancement of electric-driven microbial fumarate reduction by Mo_2_C-functionalized electrode interface corresponding first-order derivatives. Reprinted from [157] with permission from Elsevier. **d** High-resolution TEM image and the corresponding profile of calibration of the Mo_2_C particles at four sites of at C1, C2, C3, and C4. Reprinted from [163] with permission from Elsevier. **e** Interplanar spacing of Mo_2_C nanoparticles (C1–C4) with an average of 0.24 nm related to the (101) of Mo_2_C. Reprinted from [163] with permission from Elsevier. **f** High-resolution N 1*s* XPS spectrum of Mo_2_C/N-rGO shown fitted into four peaks, which are assigned to nitrogen oxides [164] with permission from Elsevier. **g** Catalytic behavior of prepared catalyst samples as a function of their composition (molar fractions of their constituent). Reprinted from [162] with permission from Elsevier

Transition metal carbides are another interesting type of material with versatile applications in energy storage, small-scale renewable energy systems, or bioelectrochemical schemes [149–151]. Among them, Mo_2_C, MXenes have shown potential for use in MECs; however, they are often combined with another element to improve their catalytic activity and conductivity. Nitrogen doping is an attractive strategy in this context, as it increases active sites, electrical conductivity, and stability of the cathode in electrochemical systems [152, 153]. Wen's research group synthesized Mo_2_C/N-doped carbon catalysts (Mo_2_C/NC) at three calcination temperatures of 700, 800, and 900 °C to optimize the catalyst's performance as a cathode in hybrid neutral-alkaline MECs for hydrogen production. The findings revealed that Mo_2_C/NC-800 (prepared at 800 °C) demonstrated superior activity compared to variants synthesized at 700 and 900 °C. At an applied voltage of 0.8 V, Mo_2_C/NC-800 achieved a current density of 13.8 A m^−2^, closely matching the 15.4 A m^−2^ attained by Pt-based catalysts, as shown in Fig. 18 [154]. This performance surpassed that of other Mo_2_C-based electrodes, including Mo_2_C/NC-700 and Mo_2_C/NC-900 which highlighted Mo_2_C/NC-800’s structural and electrochemical advantages. The catalyst further facilitated an enhanced hydrogen production rate of 170.5 L/m^2^/d at 1.0 V.

Rao et al. showed that an N-doped Mo_2_C (Mo_2_C/N-900) electrode in an asymmetric neutral-alkaline double-chamber MEC exhibited a lower electrode overpotential compared to a commercial Pt electrode and demonstrated competitive performance with Pt-based catalysts with an overpotential of 149 mV and a Tafel slope of 55.7 mV dec^−1^ [155]. Although Mo_2_C acts as a catalyst and can increase bioH_2_ and/or acetate production, it is highly desirable for modifying biofilm growth and positively impacting the microbial community [156]. In this regard, its role extends beyond catalytic activity, facilitating processes inside the chamber. Zhu and co-workers [157] focused on modifying microbial cathodes at the interface of Mo_2_C as the cathode at potentials lower than those typically required for HER initiation (i.e., − 0.36 V). They fabricated a modified Mo_2_C nitrogen-doped carbon cloth catalyst and reported superior electrocatalytic activity (Fig. 18c), with a higher current density, approximately 4.1- and 2.7-fold greater than that of carbon cloth and N-doped carbon cloth, respectively. The findings showed that by facilitating the rapid reaction of an EET mediator (riboflavin), the process could occur at potentials lower than those typically associated with HER. Although the results highlighted an insignificant contribution of Mo_2_C in HER due to the insufficient applied potential (− 0.36 V), it was concluded that functionalized Mo_2_C enhanced the electrochemical reaction kinetics of riboflavin.

Ti_3_C_2_T_x_ is another newly discovered 2D material from the TMC family, which is widely used in various energy schemes and recently utilized in limited studies as a cathode material in MECs. Intrinsically, MXenes, due to their excellent conductivity and high surface area, are strong candidates for cathode materials. It has been observed that when MXene is coated on cathodic electrodes, it can significantly increase microorganism enrichment, current density, and mass transport which attributed to these features [158–160]. Moreover, MXene acts as an efficient electron carrier between co-nutrient microbes which diminish the initial time and energy required to generate associated c-type cytochromes and conductive pili that establish an extensive extracellular bioelectric connection. Liu and co-workers [161] examined the effect of three different layouts of MXenes—MAX phase titanium aluminum carbide (Fig. 18a), multilayer (ML-Ti_3_C_2_T_x_), and few-layer (FL-Ti_3_C_2_T_x_) in MECs integrated with anaerobic digestion. They found that ML MXene, due to its higher rate of electron capacitance exchange and its promotion of methanogenic dominance (mainly *Methanosarcina* and *Methanobacterium*), achieved the highest CH_4_ production. Harnisch et al. [162] evaluated the performance of tungsten carbide (WC) as an electrocatalyst for HER in both pH-neutral and acidic electrolytes in MECs and reported HER performance improvements of 57% and 31%, respectively. The findings revealed that HER performance directly correlates with WC content (Fig. 18g), with catalysts containing over 90% WC achieving significantly higher current densities compared to catalysts with lower WC fractions or pure WO_3_ where catalysts with the highest WC content (96%) exhibited the best HER activity. It is important to note that in both environmental conditions, W_2_C and WO_2_ contributed minimally to HER activity. The study suggests that hydrogen evolution and oxidation occur on the same active surface sites of WC, with proton desorption acting as the rate-determining step (RDS). Moreover, cathodic polarization was found to reduce corrosion and enhance WC’s long-term stability. Interestingly, the HER process in bioelectrochemical systems can not only occur directly via MEC, but it is also possible to generate hydrogen in an integrated system combining MFC with an ammonium electrolysis cell (MFC/AEC).

Zhou et al. [163] fabricated a 3D electrocatalyst consisting of molybdenum carbide decorated on the surface of reduced graphene oxide (rGO) doped with nitrogen atoms through a facile two-step preparation procedure: a hydrothermal method followed by annealing at 800 °C for HER in AEC powered by MFC in an integrated design. The characterization analysis of the catalyst revealed that the Mo_2_C nanoparticles, with an average size of 4.32 nm and an interplanar space of 0.24 nm (Fig. 18e) (corresponding to the -101- plane of Mo_2_C), were uniformly dispersed on the surface of rGO, leading to a hydrogen production rate of 198 mL g_Mo2C/NGA_^−1^ in simulated ammonia wastewater and 79.2 mL g_Mo2C/NGA_^−1^ with a 74.8% ammonia removal efficiency using landfill leachate. In a similar study, Zhang et al. [164] fabricated Mo_2_C/N-doped graphene (Mo_2_C/N-rGO) through the aforementioned two-step method as a catalyst for an ammonia electrolysis cell (AEC), which was coupled with MFC as a power source for HER from ammonia-rich wastewater. The catalyst achieved a hydrogen production rate of 59 µL/g_Mo2C/N-rGO_/h with 86% efficiency under a glucose-ammonium feed and 42 µL/g_Mo2C/N-rGO_/h with 71% ammonia removal efficiency when landfill leachate was used. The XPS analysis elucidated the presence of various nitrogen species (pyrrolic, pyridinic, and graphitic) (Fig. 18f), with pyridinic highlighted as the main contributor to improving catalytic activity.

### Transition Metal Nitrides Electrocatalysts

Lu et al. [165] investigated the use of Mo_2_N nanobelt cathodes as a cost-effective and efficient alternative to Pt catalysts in MECs. Their findings revealed that the Mo_2_N nanobelt, through its high surface area and positive influence on the composition of the biofilm microbiome, promoted the growth of hydrogen-producing microorganisms and achieved catalytic activity comparable to Pt catalysts. Additionally, it attained hydrogen production rates and coulombic efficiency of approximately 0.39 m^3^ H_2_/m^3^/d and 90%, respectively.

### Hybrid Structures

#### TM-Carbon Structures

Combining transition metals like Ni and Fe with activated carbon (AC) as cathode materials in MECs has been suggested as a low-cost and facile approach for high-performance catalysts [166, 167]. Importantly, the higher performance of TM-carbon-based catalysts in MECs is not the only reason they have been brought into the spotlight, but the cost matters, which in some cases can be more than 400 times lower than currently available Pt catalysts, even more significant than the technical aspects [168].

Kim's group focused on the effect of chemical activation of the cathode electrode on the electrochemical activity of Ni-AC by activating the AC with nitric acid. The activation process increased oxygen and nitrogen species by about 16.9% and 124%, respectively, leading to an 84% improvement in hydrogen production rates (0.35 L.H_2_ L_−1_ d_−1_) and a 33% improvement in charge transfer due to enhanced proton adsorption, which facilitated the Volmer step in HER. Moreover, the effect of PVDF binder loading on Ni-AC catalysts showed an enhancement in H_2_ production with increasing concentration; however, the electrochemical activity of the acid-treated catalyst outperformed other catalysts [169]. Previous studies have also highlighted the positive role of binders like PVDF and PTFE during the fabrication of Ni-AC catalysts in enhancing hydrogen production in MECs [170].

**Fig. 19:**
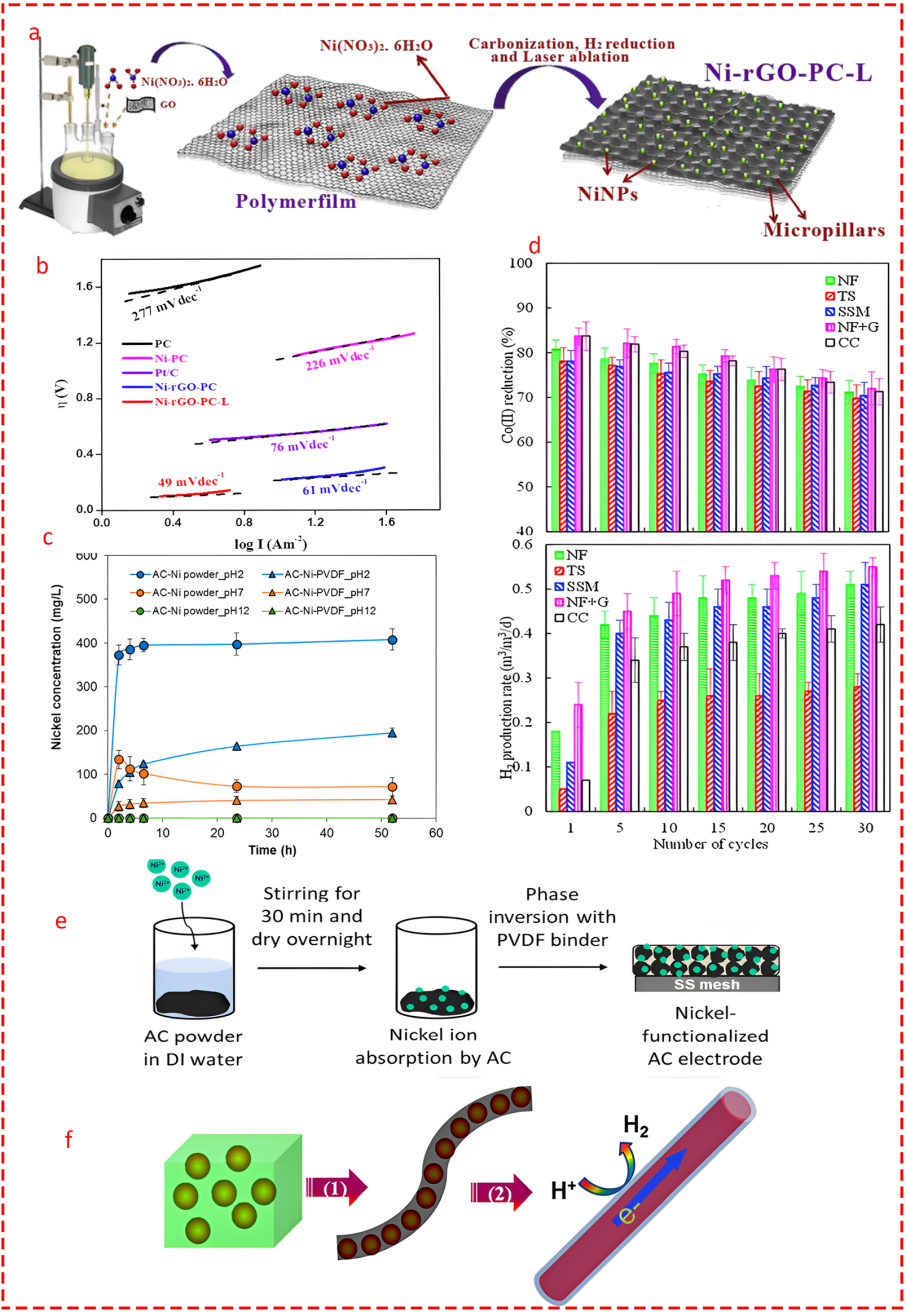
**a** Schematic representation of the synthesis of Ni-rGO-PC-L. Reprinted from [173] with permission from Elsevier. **b** Tafel plots for different electrodes. Reprinted from [173] with permission from Elsevier. **c** Concentration of Ni released from Ni-adsorbed AC powder (AC-Ni powder) and AC-Ni electrode (AC-NiPVDF) into PBS at varying pH levels (2, 7, and 12). Dissolution tests have conducted in duplicate, with error bars showing the range of duplicate results. Reprinted from [171] with permission from American Chemical Society. **d** Co(II) reduction and (top) and hydrogen production (bottom) versus number of cycles which exhibited an inverse relation [172]. **e** Synthesis procedure of Ni-Functionlized activated carbon electrode. Reprinted from [171] with permission from American Chemical Society. **f** Synthesis procedure of the Fe-carbon core–shell catalysts. Reprinted from [176] with permission from Elsevier

In an interesting study, Logan's group [171] applied a novel synthesizing method through an adsorption and phase inversion process (Fig. 19e) to decorate Ni on PVDF-coated AC as a catalyst for MECs. They reported a 50% enhancement in hydrogen production compared to pristine AC, with no increase in Ni load in an alkaline environment and 200 mg L^−1^ in a highly acidic environment (Fig. 19c). Importantly, the novel synthesizing method offered two key advantages over previous methods. First, it required only a very low amount of Ni to fabricate the cathode, making it highly resource-efficient and cost-effective. Second, the catalytic activity of the electrode could be easily reinstated by re-adsorbing nickel salts, addressing the challenge of decreasing catalyst load for long-term use by regenerating the metal without replacing the entire electrode. Wang et al. [172] evaluated the effect of various electrodes, including nickel foam (NF), stainless steel, titanium sheets, carbon cloths, and graphene oxide-coated NF, as cathodes in a double-chamber MEC focused on recovering cobalt from aqueous media. The findings revealed an inverse relationship between Co ion recovery and HER performance, where after 30 cycles, Co recovery decreased while hydrogen production increased, with GO-NF outperforming all other electrodes (Fig. 19d).

Yadav and Verma [173] decorated Ni nanoparticles on the surface of rGO using a laser ablation process as the cathode (Fig. 19a). To analyze the performance of the new catalyst, the cathode was compared with other electrodes, such as Pt, Ni-rGO without laser ablation, and pristine polyamide films. Their findings illustrated that employing laser ablation formed 3D micropillar-shaped nanoparticles on the surface of the catalyst, leading to higher catalytic activity and lower overpotential compared to other electrodes (Fig. 19b).

It is interesting to point out that several studies on transition metal-based carbon catalysts in microbial electrosynthesis systems have shown that CO₂ conversion to value-added chemicals is facilitated by in situ hydrogen generation [174, 175]. Xiao and co-workers [176] synthesized an N–Fe/Fe_3_C@C core–shell nanostructure by first drying a mixed solution of FeCl and cyanamide, followed by heat treatment under inert conditions to form a C_3_N_4_ polymer-loaded Fe-based nanoparticle, which further decomposed into graphite-wrapped Fe/ Fe_3_C nanorods upon additional annealing (Fig. 19f) as a catalyst for MECs. The N–Fe/ Fe_3_C@C catalyst demonstrated significant electrocatalytic activity, with a peak current density of 2.61 A m^−2^ which was higher than carbon cloth (CC) and carbon nanotubes (CNTs), but slightly lower than the 3.50 A m^−2^ of Pt/C. In terms of hydrogen production, N–Fe/ Fe_3_C@C achieved a rate of 0.0181 m^3^ H_2_/m^2^/day which was nearly double that of CC and CNTs, though slightly less than the 0.0230 m^3^ H_2_/m^2^/day of Pt/C. Despite the slightly reduced performance compared to Pt, the N–Fe/Fe_3_C@C catalyst offers a more cost-effective alternative, showing promise for large-scale applications in microbial electrolysis cells.

#### Bimetalic and Alloys Structures

Manuel et al. analyzed the impact of nickel alloy composition on hydrogen production in MECs by studying various nickel alloys (consisting of Cr, Mn, Fe, and Mo) across a wide range of Ni content, chemically deposited on gas diffusion cathodes. A wide range of alloy compositions tested across five scenarios, and it was reported the highest HER for the electrode with 60% Ni concentration. Moreover, it was concluded that using gas diffusion cathodes instead of solid metal sheets allowed hydrogen generated at the cathode to escape quickly that reduces the amount of hydrogen consumed by methanogens [177]. In similar studies, a higher Ni content consistently exhibited a higher rate of hydrogen production. Qin et al. [178] used carbon cloth as substrate and deposited Ni(OH) nanoparticle films using nickel (II) cyclam (1, 4, 8, 11- tetraazacyclotetradecane) as precursor at three different concentrations of 6, 15, and 23 mM and reported linear relation between the enhancing the precursor's concentration and higher hydrogen production.

**Fig. 20:**
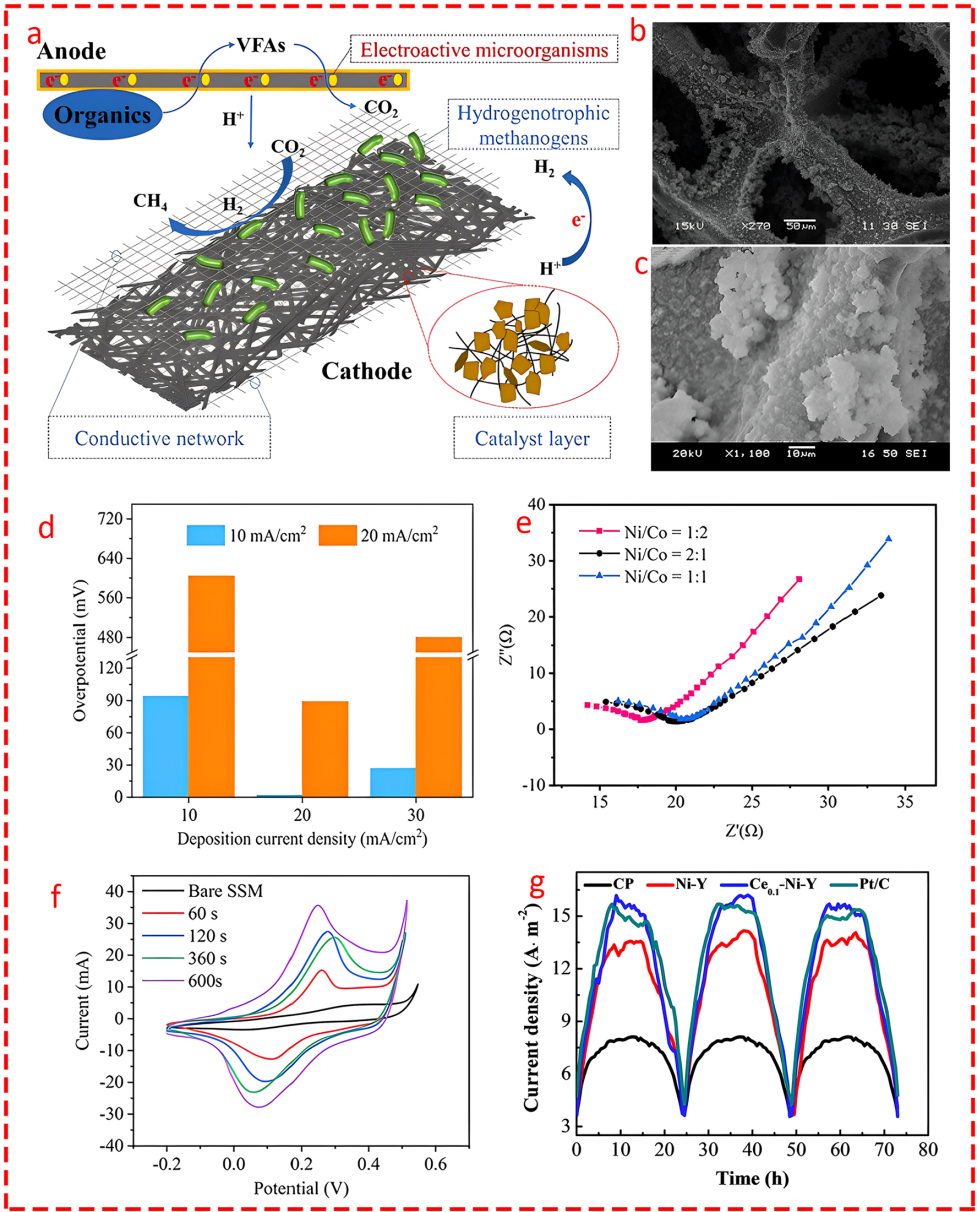
**a** Possible pathways through which the FeNi_2_-PAN2 cathode promotes methane production assisted by consuming H_2_. Reprinted from [188] with permission from Elsevier. **b** and **c** SEM image of layered double hydroxide catalysts based Ni and Fe decorated on Ni foam. Reprinted from [182] with permission from Elsevier. Overpotential, EIS curves, and CV curves of Ni-Co@SSM at **d** different electrodeposition current density, **e** different electrodeposition times, and **f** different Ni/Co molar ratio. Reprinted from [180] with permission from Springer Nature. **g** Current changes for different cathode electrodes during a batch time in three cycles. Reprinted from [179] with permission from John Wiley and Sons

Yang's group [179] doped cerium and nickel into Y-type zeolite—a material known for its superior surface area and multi-dimensional pores—to facilitate the pore structure and utilize Ce and Ni as active sites through a simple one-step hydrothermal method. They found that adding Ce to Ni improved the catalytic activity of the electrode and even delivered a competitive current density and hydrogen production compared to Pt/C over 70 h of operation (Fig. 20g). However, these results contrast with a previous study that used Ce in electrodes [142] which reported significantly lower performance than Pt/C catalysts. One possible explanation could be the structural differences and composition of the materials. In Yang et al.’s study, doping Ni and Ce into Y-type zeolite created synergies that enhanced the electrode's performance, whereas depositing Ni and Ce onto a copper sheet in the previous study negatively impacted catalytic activity compared to Pt/C. This highlights the pivotal role of selecting appropriate material compositions and their mutual interactions to optimize catalytic activity.

Li et al. [180] co-doped Ni and Co on the SS-304 sheet through electrodeposition method as biocatalyst in membrane-less MEC for degrading sulfamethazine. In order to evaluate the electrocatalytic activity of electrode, they focused on identifying various optimal parameters electrodeposition procedure including time (from 60 to 600 s), current (from 10 to 30), and the ratio of Ni/Co (1:2, 1:1, and 2:1). Their finding exhibited that deposition time of 600 s, current deposition of 20 mA cm^−2^ and Ni/Co ratio of 1:2 as the optimal conditions which delivered the highest current, lowest over potential and resistance, respectively (Fig. 20d-f), also leading to highest rate of sulfamethazine degradation.

In a similar study [181], the synergy between co-doping Ni and Co on NF through an electrodeposition procedure was demonstrated. Further, the catalyst surface was modified by coating sulfur atoms onto it, leading to improved electrical conductivity and more active sites, which outperformed other electrodes in hydrogen production rates. However, the synthesizing procedure remained constant for electrode fabrication.

Lu et al. [182] co-doped Ni and Co onto an SS-304 sheet through an electrodeposition method as a biocatalyst in a membraneless MEC for degrading sulfamethazine. To evaluate the electrode's electrocatalytic activity, the study focused on optimizing various parameters in the electrodeposition procedure, including deposition time (60–600 s), current density (10–30 mA cm^−2^), and the Ni/Co ratio (1:2, 1:1, and 2:1). Their findings showed that a deposition time of 600 s, a current density of 20 mA cm^−2^, and a Ni/Co ratio of 1:2 were optimal conditions, delivering the highest current, lowest overpotential, and resistance (Fig. 20d–f), which also resulted in the highest rate of sulfamethazine degradation.

Zhao et al. [183] investigated the catalytic performance of Ni/NiO nanocomposites decorated on carbon paper in MECs, focusing on elucidating the HER kinetic mechanism and transition steps. Initially, Ni provided excellent catalytic activity due to efficient electron transfer; however, during long-term operation (60 days), corrosion led to the formation of NiO on the surface and Ni dissolution. The atomic ratio of NiO increased from 27.88% to 38.46%. The Tafel curves, using the Butler–Volmer equation, and the Tafel slope for Ni-1 to Ni-5 (over 60 days of operation), indicated a decline in catalytic efficiency, with the Tafel slope increasing from 35 to 86 mV dec^−1^, highlighting reduced electron transfer efficiency and HER activity. The kinetic analysis revealed that the HER mechanism followed a Volmer–Heyrovsky pathway, with the RDS transitioning from Heyrovsky to Volmer over time. Additionally, the formation of NiO and sediment accumulation on the electrode further diminished performance which emphasized the need for better catalyst stability in long-term MEC operations. Sattar et al. [184] decorated Ni onto two different substrates, titanium and graphite felt, through an electrodeposition method toward fabrication of high-efficient catalyst. The MEC was operated over 12 months, and the results showed that although the Ni–Ti catalyst had a smaller projected area (3.5 × 3.5 cm^2^) compared to Ni-GF (5 × 5 cm^2^), it generated a higher hydrogen biogas. Although the mechanism behind this was not examined by the authors, two possible explanations could be the synergies between Ni and Ti as well as the higher stability of the Ni–Ti composition compared to Ni-GF.

Mitov et al. [185] coated W and Mo (two prominent low-cost catalysts) on the surface of NF to fabricate two cathode electrodes and examined their performance in a single-chamber MEC for biohydrogen production. The results illustrated that Ni-W achieved slightly higher hydrogen production (0.01 m^3^/m^3^/d) but exhibited a significant difference in intrinsic electrocatalytic activity—nearly six fold higher—compared to Ni-Mo. The authors concluded that Mo possibly covered the entire surface of NF with a thick layer which meant that the only catalytic activity came from Mo and not NF, resulting in very low intrinsic electrocatalytic activity. Similarly, other researchers coated Ni-W and Ni-Mo on carbon cloth and reported 33% higher H_2_ production for Ni-W compared to Ni-Mo catalysts [186].

#### MOF Structures

Although MOFs are not considered a newly discovered class of materials compared to recently discovered high-performance materials, their applications in bioelectrochemical systems remain in their infancy stage. Interestingly, MOFs were not been adopted as electrode catalysts in MECs until 2022, when two research groups independently fabricated Fe/Ni-MOF and Ni/Co-NC MOF as cathode electrodes in an integrated MEC-AD system for methane production [187, 188]. Zheng et al. [189] used ZIF-67 in the cathode of an MEC-AD integrated system; however, they did not examine the exact effect of ZIF in the system and instead focused on optimizing the applied voltage.

Nevertheless, it was demonstrated that MOFs can be effectively introduced into biocathodes to improve heavy metal removal rates while simultaneously increasing biohydrogen production rates [190]. Li et al. [191] fabricated a solar-based microbial electrochemical system using BiVO_4_-RuO_2_-IrO_2_ and a highly stable ZIF-67/g-C_3_N_4_ MOF as photoanode and photocathode, respectively, for CO_2_ conversion into acetate and hydrogen production. The structure's performance was evaluated under various scenarios, and the results showed high hydrogen yields when the photoanode and photocathode were simultaneously utilized. Although the structure was fabricated using low-cost and abundantly available materials, scalability of the proposed structure remained as a questionable challenge.

While the main objective of MEC research has traditionally focused on enhancing biohydrogen production through HER, some researchers have shifted their focus toward enhancing methane production in MECs or integrating MECs with anaerobic digestion systems to boost methane yield using TM-MOF electrodes. The mechanism involves the in situ production of hydrogen, which is consumed within the system to increase methane production. For instance, Wang et al. [188] fabricated a cathode by combining FeNi_2_-PAN_2_ with a stainless steel mesh to enhance methane production in MECs. The cathode facilitated the electrochemical HER, converting protons (H^+^) into H_2_ using electrons transferred from the anode. The hydrogenotrophic methanogens, enriched on the cathode surface, utilized the generated H₂ as an electron donor to reduce carbon dioxide (CO_2_) into methane (Fig. 20a). The high surface area and catalytic properties of the FeNi_2_-PAN_2_ cathode promoted greater H₂ availability that enabled these methanogens to efficiently convert CO_2_ into CH_4_ leading to significant increase in methane yields. This process illustrates the critical role of hydrogenotrophic methanogens in converting H_2_ into methane that driven by the enhanced catalytic activity of the FeNi_2_-PAN_2_ cathode.

## Large-Scale Implication Strategies

### Life Cycle Assessment of TM-based Electrocatalysts

Generally, life cycle assessment is applied in bioelectrochemical energy systems to compare various schemes such as MEC, MFC, and MDC, either as standalone systems or in integrated design [192, 193]. Interestingly, in terms of LCA, MEC schemes outperform other BESs, particularly when wastewater treatment is the benchmark, due to their ability to produce high-value products (mostly H_2_ and, in some cases, H_2_O_2_) per unit of energy consumption and their lower environmental impacts [194, 195]. For instance, Pant et al. [196] conducted a comparative LCA analysis on MFCs, MECs, and MDCs to assess their potential for converting wastewater into energy and valuable products from environmental and economic perspectives. Key findings suggested that while MFCs are competitive in converting organic waste into electricity, MECs deliver superior performance due to their ability to produce hydrogen at a large scale, whereas MDCs face challenges with power density. Collectively, MEC systems tend to outperform MFCs and MDCs when environmental impacts and energy outputs are compared. Through a LCA analysis, Oksuz et al. [197] evaluated the Human Health Non-Cancer Potential (HHNCP) impact of MECs, considering different MEC components. The findings demonstrated that electrode production was identified as the primary contributor to HHNCP, primarily due to toluene equivalent emissions during manufacturing which accounted for 237.3 kg of toluene for low estimates and 347.2 kg for high estimates.

**Fig. 21:**
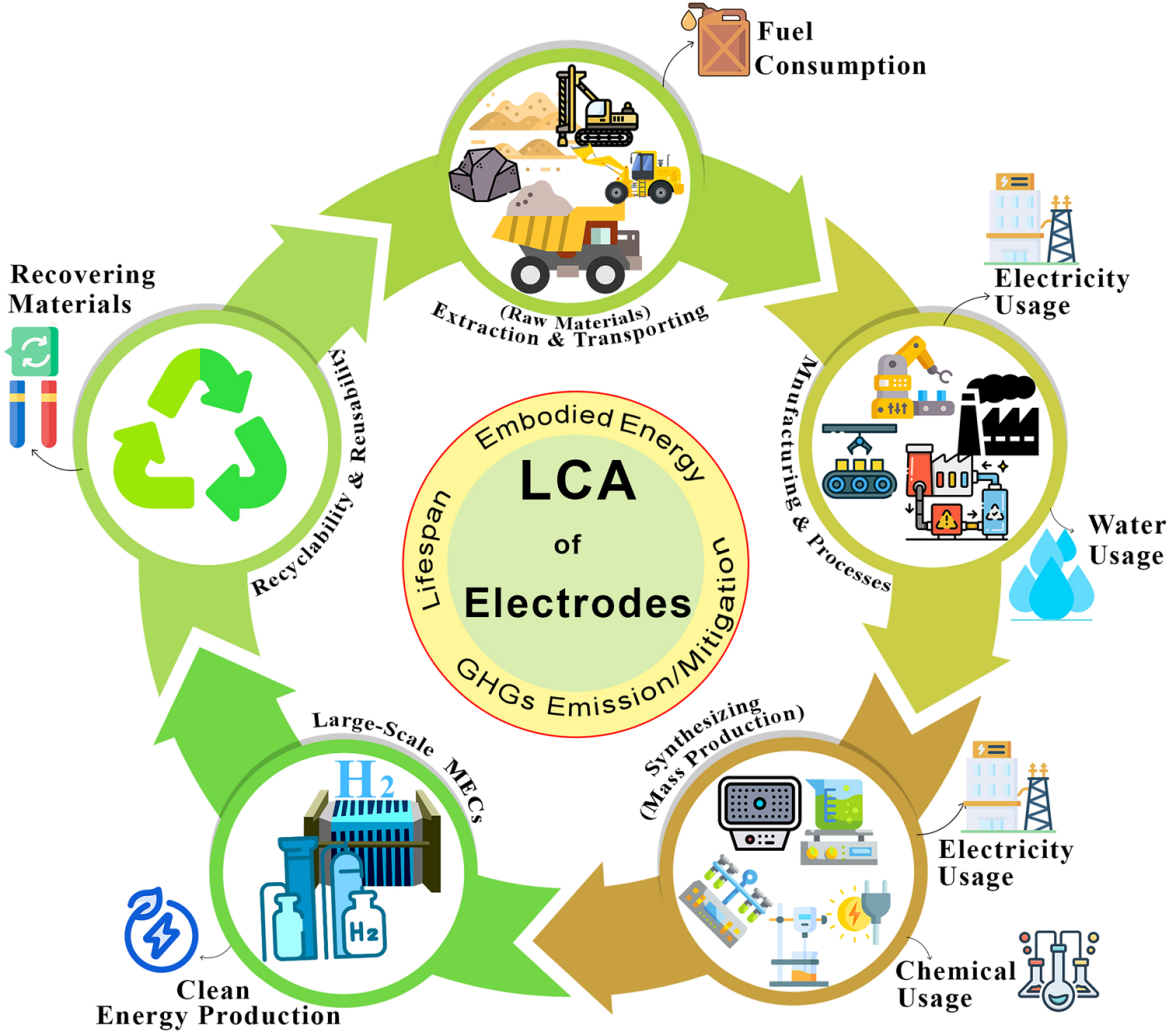
General concept on life cycle assessments (LCA) of electrodes

On a broader scale, the ultimate goal of employing TM-based electrocatalysts is to fabricate high-efficiency electrodes that contribute to environmental protection by enabling CO_2_ reduction, green chemical/fuel production (e.g., hydrogen, acetate), and wastewater treatment. However, the processes that lead to these benefits must not have hidden environmental hazards, especially when scaled for large-scale applications. During manufacturing, materials undergo various processes that consume electricity, water, and fuels, leading to greenhouse gas emissions (Fig. 21). In a nutshell, the concept of embodied energy involves calculating the total energy consumed throughout the process—from raw material extraction to the production of the final product. In this regard, the LCA of utilized materials should be carefully evaluated.

In an interesting study, Anasori’s group analyzed the LCA of MXene production in batch mode for two scenarios of gram-scale and kilogram-scale production. They found that the gram-scale scenario (19.2 g per batch) required nearly nine times the cumulative energy demand of the kilogram-scale scenario (0.8 kg per batch). Additionally, for identical applications, the CO_2_ emissions for MXene were calculated at 428 kg- CO_2_/kg-MXene, compared to 23 kg- CO_2_/kg for aluminum foils and 8.75 kg- CO_2_/kg for copper foils [198]. Moreover, it was shown that energy source (electricity consumption) is the determining parameter in the LCA, rather than chemical usage. On the other hand, Hachach et al. [199] performed an LCA on the large-scale production of MoS_2_ through a solvothermal process and showed that by substituting LiOH with NaOH in the process the overall negative impacts reduced by 56%. Similarly, Ntouros and co-workers [200] conducted an LCA analysis on the fabrication of ZIF-8 through five different synthetic routes and found that chemical usage contributed to more than 85% of the environmental impacts. Grande et al. [201] also found that during the fabrication process of MOF-74, substantial environmental impacts arose from the solvents utilized during synthesis. Wei et al. examined the energy consumption and greenhouse gas (GHG) emissions associated with different nickel products. Nickel oxide (76% Ni) showed the highest values which requiring 370 GJ t^−1^ and emitting 30 tCO_2_-eq/t, followed by nickel metal (100% Ni) at 174 GJ t^−1^ and 14 tCO_2_-eq/t, ferronickel (35% Ni) at 110 GJ t^−1^ and 6 tCO_2_-eq/t, and nickel pig iron (10% Ni) at 60 GJ t^−1^ and 7 tCO_2_-eq/t. The elevated values for nickel oxide were primarily attributed to its heavy reliance on fossil fuels and the energy-intensive processing of laterite ores. This underscores that different types of TM-based electrocatalysts that produced via various synthesizing procedures can impact the environment in different pathways, which should be thoroughly understood as a primary step toward large-scale practical applications. However, one important consideration is that in early stage and recent LCA studies on MEC systems, the applied voltage is often assumed to be approximately 0.5 V. This assumption is unrealistic for real-world applications, as the minimum voltage in most laboratory-scale studies is 0.6 V [202–205] or as high as 0.9 V under actual conditions [206].

### Possible Strategies toward Rational Design of High-Efficient Catalysts in MEC

The rational design of heterogeneous catalysts is considered as the cornerstone of applying TM-based electrocatalysts to modifying high-performance MECs for biohydrogen production. However, the main obstacles in designing catalysts for MECs can be viewed from two perspectives. The first problem is the frequent use of traditional, well-established catalysts based on previous experiments [117], and the second is employing TM-based electrocatalysts without any particular design strategy. Indeed, designing high-efficient catalysts for real-world applications should adopt through new strategies. Considering the highly complex environment of MECs, catalyst design should integrate multiple techniques. In this regard, inverse design of optimal catalysts which tailored based on the nature of the wastewater, the dominant type microorganisms, and structural design of the MEC (e.g., single- or double-chamber systems, various types of binders), through techniques such as gradient-based optimization and alchemical transformations, is a highly desirable approach for developing innovative catalysts. Freeze et al. reviewed the potential of inverse design in the pursuit of new catalysts and concluded that one of the main gaps is bridging the space between experimental and computational approaches [207]. This represents an important but often overlooked aspect of catalyst design in MECs. Bridging computational methods with experiments could pave the way for new avenues toward diverse TM-based electrocatalysts. Recently, a web-based catalytic design tool based on topographic steric maps was introduced to visualize and interpret the steric environment of transition metal complexes in a 3D descriptor in order to help for understanding the steric effects on reactivity [208]. This tool highlights various vital factors contributing to catalytic activity and lead to the design of novel transition metal complexes with enhanced catalytic performance. Additionally, advanced modeling approaches such as microkinetic modeling have shown promising results in designing heterogeneous catalysts. Data from microkinetic analysis empower researchers to identify material critical properties for enhanced catalytic performance [209]. Though exploring new catalysts by megalibraries is an intriguing approach, however, it may not feasible in this context due to the lack of extensive data [210] (Tables 1 and 2).

**Table 1:** Summary of TM-based catalysts from different family materials applied in MECs

Class of material	Type of system	Catalyst	Synthesizing method	Applied voltage (V)	Period of operation	H_2_ rate production	Refs
TMO	Double chamber	NiFe_2_O_4_-WO_3_	Hydrothermal method	> 300 mV	–	1.46 ± 0.25 mL/h	[95]
TMO	Single chamber	Cu-Co(OH)F/NF	Hydrothermal method	0.8	30 h	214.5 mL/cycle	[118]
TMO	Single chamber	ZnFe_2_O_4_/g-C_3_N_4_	Sol–gel method	0.4, 0.6, 0.8	144 h	1.70 ± 0.04 m^3^/m^3^/day	[98]
TMO	Single chamber	ZnFe_2_O_4_/g-C_3_N_4_	Sol–gel method	0.6	288 h	0.55 m^3^/m^3^/day	[97]
TMO	Single chamber	Ni/ MoO_2_/ MoO_3_	Normal pulse voltammetry	1.5	288 h	–	[116]
TMO	Double chamber	NiO/rGO	Co-precipitation, reduction	1	5 d	4.38 mmol/L/D	[279]
TMO	Double chamber	Co_3_O_4_ /rGO	Co-precipitation, mixing & reduction	1	5 d	3.66 mmol/L/D	[279]
TMO	Double chamber	NiO/NF	Co-precipitation, calcination	1	120 h	3.39 ± 0.03 mmol/L/D	[115]
TMO	Double chamber	Co_3_O_4_/NF	Co-precipitation, calcination	1	120 h	3.08 mmol/L/D	[115]
TMO	Double chamber	NiMoO_4_	Sonochemical precipitation	1	5 d	4.28 mmol/L/D	[112]
TMO	Double chamber	NiMoO_4_	Hydrothermal method	0.8	19 d	81 ± 3 L/L/d	[117]
TMO	Double chamber	NiMoO_4_	Electrochemical synthesis	0.8	19 d	48 ± 4 L/L/d	[117]
TMO	Double chamber	NiCo_2_O_4_-rGO	Co-precipitation, reduction	1	5 d	6.08 ± 0.13 mmol/L/D	[106]
TMO	NA	Mn_3_O_4_/ Graphite Fe_3_O_4_/Graphite Fe_2_O_3_/Graphite TiO_x_/Graphite	Pyrolysis	1	–	1.6 mL/cm^2^/h 1.2 mL/cm^2^/h 0.8 mL/cm^2^/h 1 mL/cm^2^/h	[102]
TMD	Dual chamber	Exfoliated MoS_2_	Carbon cloth Geobacter sulfurreducens	0.6	NA	0.113 m^3^/m^3^/day	[120]
TMD	Single chamber	MoS_2_-Cu-rGO	Carbon felt	0.7	2 h	0.449 ± 0.027 m^3^/m^3^/day	[125]
TMD	Dual chamber	MoS_2_/CNT	Carbon brush	0.8	48 h	0.0101 ± 0.0007 m^3^/m^2^/day	[122]
TMD	Single chamber	MoS_2_-graphene	Graphite brush	NA	10 h (5 cycles)	0.183 m^3^/m^3^/day	[123]
TMD	Single chamber	MoS_2_/ Cu_2_O	Bioanode from an MFC	−0.8	50 d	2.72 m^3^/m^3^/day	[124]
TMD	Dual chamber	MoS_2_ + Nano carbon	Carbon fiber brush	0.9 (V)	60 h (3 cycles)	0.152 ± 0.002 m^3^/m^2^/day	[126]
TMD	Single chamber	MoS_2_ on carbon cloth	Nafion binder mixing with MoS_2_ powders	0.7	46 h	1.2 m^3^/m^3^/day	[129]
TMD	Single chamber	MoS_2_ on carbon paper	Electrodeposition	Varied	17 d	0.39 ± 0.05 m^3^/m^3^/day	[127]
TMD	Single chamber	MoS_2_ on carbon paper	Impregnation, heat treatment	Varied	17 d	0.26 ± 0.03 m^3^/m^3^/day	[127]
TMD	Single chamber	MoS_2_ on carbon paper	Drop coating	Varied	17 d	–	[127]
TMD		MoS_2_ on stainless steel	Using a catalyst paste	0.9	Almost 2 d	0.67 m^3^/m^3^/d	[128]
TMC	Dual chamber	Mo_2_C/N-rGO	Solid carburization	0.78	240 h	59 mL/g_catalyst_	[164]
TMC	Dual chamber	Mo_2_C-/N-doped graphene	Hydrothermal annealing method	0.9	25 h	198 mL/g_catalyst_	[163]
TMC	Dual chamber	Mo_2_C- Nitrogen doped	Carbon fiber brush	0.8	24 h	103.0 L/m^2^/d	[155]
TMC	Dual chamber	Mo_2_C- Nitrogen doped loaded on carbon cloth	Carbon brush	1	~ 200 h	170.5 L H_2_/m^2^/d	[154]
TMP	Dual chamber	CoP-NF	Hydrothermal method, phosphating	0.7	30 d	222 ± 20.3 mL/L/d	[135]
TMP	Dual chamber	Ni_5_P_4_-NiP_2_	Phosphorization	0.8	60 d	9.78 ± 0.38 mL/d/cm^2^	[140]
TMP	Dual-chamber membrane-less	Ni-Co-P/SS	Electrodeposition	0.6	7 d	0.16 ± 0.002 m^3^/m^3^/d	[280]
TMP	Dual-chamber membrane-less	Ni-Co-P/Cu	Electrodeposition	0.6	7 d	0.14 ± 0.002 m^3^/m^3^/d	[280]
TMP	Dual chamber	Ni_2_P/C	Solution-phase method	0.9	11 d	0.29 ± 0.04 L/L	[139]
TMP	single chamber	Ni–P coating on Ni foam	Chemical plating	0.5–0.9	30 d	2.29 ± 0.11 L/L/d	[138]
TMP	single chamber	Ni-W–P	Two-step electrodeposition method	0.9	20 h	1.09 m^3^/m^3^/d	[142]
TMP	Dual chamber	Ru/CoP nanosheets	Chemical reduction + phosphating	0.8	72 h	0.1434 ± 0.0082 m^3^/m^2^/d	[137]
TMN	single chamber	Mo_2_N	Hydrothermal method + annealing	0.77	110 cycles	0.39 ± 0.14 m^3^/m^3^/d	[165]

**Table 2:** Summary of hybrid TM-based catalysts applied in MECs

Class of material	Type of system	Cathode	Synthesizing method	Applied voltage	Period of operation	H_2_ rate production	Refs
Hybrid	Single chamber	AC-Fe/SS	Adsorption and phase inversion method	0.6	258 h	0.003 ± 0.0004 L-H_2_/L-reactor/day	[166]
Hybrid	Double chamber	Acid treated AC/Ni	Phase inversion method	0.9	10 d	0.35 ± 0.02 L- H_2_/L-day	[169]
Hybrid	Double chamber	AC/Ni	Phase inversion method	0.9	10 d	0.19 ± 0.01 L- H_2_/L-day	[169]
Hybrid	Double chamber	AC/Ni	Phase inversion method	0.9	30 d	1.1 ± 0.1 L- H_2_/L-reactor/day	[171]
Hybrid	Double chamber	AC-pNi4.8	Phase inversion method	0.9	22 d	0.38 ± 0.04 L- H_2_/L-reactor/day	[167]
Hybrid	Double chamber	Ni powder without AC	Phase inversion method	0.9	22 d	0.28 ± 0.02 L- H_2_/L-reactor/day	[167]
Hybrid	Single chamber	Ni/AC/PTFE	Adsorption and phase inversion method	0.9	5 d	1.88 L- H_2_/L-reactor/day	[170]
Hybrid	Single chamber	Ni/AC/PVDF	Adsorption and phase inversion method	0.9	5 d	1.56 L–H₂/L-reactor/day	[170]
Hybrid	Single chamber	Ni on Carbon paper	Electrodeposition	0.7	140 h	6.7 mL of H₂ per cycle	[168]
Hybrid	Single chamber	Ni-rGO	Carbonization, and laser ablation	0.8	9 d	4.22 ± 0.21 m^3^- H_2_/m^3^	[173]
Hybrid	Double chamber	NF + G	Annealing	0.2 to 0.7	180 h	0.55 m^3^- H_2_/m^3^-reactor/day	[172]
Hybrid	Double chamber	Ni(OH)_**2**_	Electrodeposition	0.8	30 d	0.014 ± 0.002 m^3^- H_2_/m^2^-cathode/day	[178]
Hybrid	Single chamber	60% Ni in gas diffusion cathodes	Chemical deposition	1	7–15 d	4.14 L- H_2_/L-reactor/day	[177]
Hybrid	Dual chamber	Ni and cerium-doped Y zeolite	Hydrothermal synthesis	0.7	3 months	0.31 ± 0.01 m^3^- H_2_/m^3^-reactor/day	[179]
Hybrid	Single chamber	Ni-doped Y zeolite composite	Hydrothermal synthesis	0.7	3 months	0.19 ± 0.01 m^3^- H_2_/m^3^-reactor/day	[179]
Hybrid	Single chamber	Nickel–cobalt–sulfur on Ni foam	Electrodeposition	0.6, 0.8, and 1.0	4 months	0.68 m^3^- H_2_/m^3^-reactor/day	[181]
Hybrid	Single chamber	Ni–Fe-layered double hydroxide on Ni foam	Hydrothermal synthesis	0.8	200 h	2.01 ± 0.01 to 2.11 m^3^- H_2_/m^3^-reactor/day (based on the substrate)	[182]
Hybrid	Single chamber	Carbon-based Ni/NiO nanocomposite	Electrodeposition	0.7	60 d	0.68 ± 0.05 m^3^- H_2_/m^3^-reactor/day	[183]
Hybrid	Dual chamber	Ni-coated titanium	Electrodeposition	0.5–1.0	36 d	0.39 ± 0.01 m^3^- H_2_/m^3^-reactor/day	[184]
Hybrid	Dual-chamber	Ni-coated graphite felt	Electrodeposition	0.5–1.0	36 d	0.33 ± 0.03 m^3^- H_2_/m^3^-reactor/day	[184]
Hybrid	Single-chamber	Ni-tungsten on Ni foam	Electrodeposition	0.6	12 d	0.14 ± 0.01 m^3^- H_2_/m^3^-reactor/day	[185]
Hybrid	Single-chamber	Ni-molybdenum on Ni foam	Electrodeposition	0.6	12 d	0.13 ± 0.01 m^3^- H_2_/m^3^-reactor/day	[185]
Hybrid	Single-chamber	Ni-molybdenum on carbon cloth	Electrodeposition	0.6	12 week	2.0 m^3^- H_2_/m^3^-reactor/day	[186]
Hybrid	Single chamber	Ni-tungsten on carbon cloth	Electrodeposition	0.6	12 week	1.5 m^3^- H_2_/m^3^-reactor/day	[186]

### Techno-Economic and Cost Analysis of TM-Based Catalysts

In most TM-based electrocatalysts, economic and cost analyses have not been extensively examined, with technical aspects of electrodes remaining the primary focus for researchers. Kumar et al. [211] in a critical review stated that although the size of global market for PEM fuel cells expected to soar by 47.60 billion USD until 2026, the cost of electrode is still the main drawback for mass production and commercialization of this technology. Similarly, Ruiz-Lopez et al. [212] emphasized on the crucial role of techno-economic analysis of catalyst design on the development of electrochemical CO_2_ conversion to valuable chemicals. Importantly, the economic analysis of MECs is crucial, particularly for the cathode electrode, as it has been identified as the major cost contributor which accounts for nearly 50% of MEC construction costs (Fig. 20d) [206]. Table 3 presents the cost analysis of several studies on TM-based electrocatalysts. As observed, the benchmark for most studies for evaluating the cost-effectiveness of catalysts is Pt which is considered as the prominent candidate, but other materials, such as MoS_2_-Cu_2_O and Ni foam, are also taken into consideration. Currently, there is no comprehensive economic model for MEC cost analysis comparable to the established economic models used in renewable energy systems [213–217]. One plausible economic model could involve utilizing the cost–benefit ratio based on selling the primary product or environmental benefits, such as GHG reductions (e.g., CO_2_) and their potential sale in the global market [218–220].

**Table 3:** Cost comparison of several TM-based catalysts with commercial catalysts

Class of material	Catalyst	Price	Benchmark	Cost ratio	Refs
TMO	ZnFe_2_O_4_	102 $	MoS_2_-Cu_2_O	0.095 Pt catalysts	[97]
	ZnFe_2_O_4_	102 $	MoS_2_-Cu_2_O	0.095 Pt catalysts	[98]
	NiMoO_4_	~ 40–41 $	Pt/C	700 times lower cost	[112]
TMP	Ni/W@P	0.62 $	Pt/C	0.79 Pt/C catalyst	[142]
	Co-P	284 $/m^2^	Pt/C	0.21 Pt/C catalyst	[135]
	Ni_5_P_4_/NiP	80.66 €/m^2^	Pt/C	0.18 of Pt	[140]
TMD	MoS_2_	11 $/m^2^	Pt/C	194 times lower price	[121]
	Exfoliated MoS_2_	127 $/mg/cm^2^	Pt/C	Not mentioned	[120]
	MoS_2_	2.4 $/m^2^	Pt/C	0.017 Pt catalysts (5 mg Pt)	[127]
Hybrid	Ni-Activated carbon	18 $/m^2^	Ni Foam	0.9 Pt	[171]
	Ni-AC-PTFE	0.21	Pt/C	0.22 Pt	[170]
	Ni foam + graphene	530 $/m^2^	Carbon cloth	0.22 carbon cloth	[172]

Recently, Jiang et al. [221] proposed a simple economic evaluation method to assess MEC performance based on energy utilization and the market price of selling hydrogen gas as follows:

$$\begin{aligned}   {\text{Profitability}}~ =  & \frac{{{\text{Revenue}}~{\text{of}}~{\text{selling}}~{\text{Hydrogen}}~\left( \$  \right)}}{{{\text{Cost}}~{\text{of}}~{\text{electricity}}~\left( \$  \right)}}~ \\     =  & {\text{Hydrogen}}~{\text{production}}~{\text{per}}~{\text{kWh}}~{\text{electricity}}~\left( {{\text{kgH}}_{2} /{\text{kWh}}} \right)~ \\    \quad  &  \times ~\frac{{{\text{Hydrogen}}~~{\text{selling}}~{\text{price}}~\left( {\$ /{\text{kgH}}_{2} } \right)~}}{{{\text{Cost}}~{\text{of}}~{\text{electricity}}~\left( {\$ /{\text{kWh}}} \right)}} \\  \end{aligned}$$

We can define the latter term (the ratio of H_2_ selling price to electricity price) as the energy price ratio. The former term can then be derived as:

$${H}_{2} \text{production per kWh electricity }({\text{kgH}}_{2}/\text{kWh}) = \frac{1}{26.8}V \times \frac{{r}_{\text{cat}}}{\text{Voltage} V} \times \frac{kg{H}_{2}}{kWh}$$

where ${r}_{\text{cat}}$ is the cathodic H_2_ recovery.

As the equation suggests, H_2_ production per kilowatt of electricity used is a function of voltage required and the cathodic H_2_ recovery.

To make a MEC profitable, a profitability > 1 is desired, which implies:

$${H}_{2} \text{production per kWh electricity} \times \text{Energy Price Ratio}>1$$

or

$$\frac{1}{{H}_{2} \text{production per kWh electricity}}<\text{Energy Price Ratio}$$

The above economic model could integrate with above-mentioned approaches to develop a multi-criteria economic model not only based on the cost–benefit ratio of system but also includes the environmental parameters [218].

### Pilot Studies and Scale-Up Consideration

Pilot-scale studies are a critical step in translating a novel technology into real-world applications, as many technical challenges and actual costs are revealed when the system is scaled up and monitored under long-term operation. Previous scale-up studies using Pt electrodes up to 70 cm in size demonstrated [206] that performance can drastically drop to half of the laboratory-scale performance under identical conditions. Considering the high price of Pt-based catalysts, most pilot-scale studies have employed stainless steel as the cathode electrode [202, 222–226]. However, SS cathodes significantly decrease HER kinetics. On the other hand, due to the limited number of pilot-scale studies, few researchers have utilized TM-based cathodes. Gil-Carrera and co-workers [227] tested a 10 L MEC by electrodepositing Ni particles (0.25–0.30 mg cm^−2^) onto the surface of carbon paper as the cathode for treating municipal wastewater. During 45 days of operation, the MEC achieved COD removal efficiencies of 60%–76% at an energy consumption of 0.9 Wh/g-COD. Escapa et al. [228] in a semi-pilot study utilized gas diffusion Ni-based electrodes (Ni load of 0.4 mg cm^−2^) to examine cathode efficiency in single- and double-chamber MEC configurations and reported higher hydrogen production rates for the double-chamber setup but lower COD removal compared to the single chamber. Recently, Baeza’s research group [229] in a pilot-scale study using a 100 L MEC system achieved to the highest reported hydrogen production of around 19.07 ± 0.46 L H_2_/m^2^/day using nickel foam cathodes with the dimension of 15 cm × 15 cm × 1.5 mm) which highlighted their superior performance compared to traditional stainless steel wool cathodes. The porous structure of the Ni-foam cathode enhanced HER and gas recovery efficiencies (81 ± 1%), but it was economically challenging due to high costs (540 € kg^−1^ compared to 10 € kg^−1^ for stainless steel). While nickel foam cathodes offer higher efficiency, the researchers emphasized the need to balance performance gains with economic feasibility for larger-scale applications.

It should be noted that in pilot-scale studies, system performance from all aspects—technical and economic—must be evaluated through long-term operation. Factors such as electrode durability, average production rates, system maintenance costs, and replacement costs must be considered in feasibility studies. Moreover, it is highly recommended to explore other TM-based catalysts (e.g., Fe and the low-cost TMDs and TMOs mentioned above) that satisfy the criteria for MECs in real-world applications, including acceptable catalytic activity, low cost, and durability. Such studies could help to elucidate the pros and cons of each family of TM-based electrocatalysts.

## Role of AI for the Development of TM-based Catalysts in MECs

In the last decade, most scientific disciplines have witnessed the powerful role of AI in advancing their fields. Intuitively, AI utilizes big data from previous experiments, combined with computational methods and machine learning (ML) algorithms, to predict new compounds among hundreds of thousands of possible structures, including precise fractions of each element. For example, in the field of pharmaceutical science, a new type of antibiotic targeting antimicrobial-resistant pathogens—considered a potential agent for initiating the next pandemic [230] —has been discovered using AI [231] while in traditional methods, finding a new antibiotic takes years of experiments with huge costs. This highlights the crucial role of AI, as the development of new antibiotics typically takes more than ten years.

### Current Data-Driven and Artificial Intelligence Applications in MEC

Classic data-driven methods have been applied in MECs through a wide range of approaches, including well-established methods such as response surface methodology (RSM), artificial neural networks (ANN), and genetic algorithms, either in combination or as standalone algorithms. These methods have been utilized for various applications, ranging from process modeling and simulation [227–229] to parameter optimization [232–234]. It is worth noting that advanced AI methods, such as autoencoder deep learning, have demonstrated high potential in examining factors that affect biofilm formation [235] which is crucial in MECs systems. Agha et al. [236] developed an AI-driven method to identify influential parameters by predicting energy recovery and biohydrogen production using an integrated ANN and ANFIS method. They reported that voltage and electrical conductivity were the most important factors affecting system performance. However, a major flaw in their study was the limited dataset for the AI model, which was derived from only a single research paper which make the model's accuracy questionable. Recently, Yoon et al. [237] developed an AI model based on random forests to predict current generation and hydrogen production in single- and double-chamber MECs integrated with AD. The model was trained on datasets of various substrates from 29 published MEC studies and achieved an acceptable error with R^2^ > 92%, highlighting its accuracy. As observed, not only are the AI-based studies in MECs limited, but the primary focus is generally on performance prediction and process simulation. One possible strategy could be to employ successful practices of AI-based methods in catalyst design from similar electrochemical energy schemes and apply these practices to the fabrication of high-performance electrocatalysts for MECs. These possible approaches are discussed in the next section.

### AI’s Potential in Development of High-performance Catalysts

Historically, the fabrication and design of catalysts have heavily relied on the Edisonian trial-and-error approach. While the role of AI in catalyst design has been explored by some researchers, Takahashi’s group was among the first to propose the term "catalyst informatics," which stands on three key concepts for catalyst design: (i) high-throughput experimentation/computing methods, (ii) the application of machine learning algorithms, and (iii) a specific platform for catalyst design [238]. Their group later developed a web-based platform called CADS (Catalyst Acquisition by Data Science), which remains in its infancy stage [239]. However, it is important to point out that a major drawback of traditional ML methods is the selection of appropriate descriptors. Unlike deep learning methods, which autonomously select the best descriptors due to their ability to process large datasets (typically around 1000–10,000 data points), traditional ML methods require manual descriptor selection. Hao and co-workers [240] determined how the combination of Click chemistry and fast scan voltammetry with a deep learning method that constructed by a multilayer deep convolutional neural network (CNN) model could result in detection of single-atom copper ions. In another study, Taniike et al. [241] presented a novel technique called automotive feature engineering (AFE) as a versatile approach to facilitate effective ML for small datasets of solid catalysts characterized by different compositions. Their findings revealed the superiority of AFE in designing highly expressive features tailored to a specific catalyst system without requiring prior knowledge of the system. However, it should be noted that the AFE was relied heavily on high-throughput experimentation, which may be a limiting factor in its widespread development. Lai et al. [242] were the first to employ an AI-based large-language model (LLM), specifically ChatGPT, to extract data from 2410 articles using the Elsevier Text Mining API for designing novel catalysts. The LLM refined the dataset to 603 articles by training it to identify important parameters, which were then applied in Bayesian optimization for catalyst synthesis for ammonia production. This approach identified four crucial parameters for ammonia synthesis: activation pressure, activation duration, activation temperature, and heating rate. Despite this progress, the study lacked validation, although the AI workflow predicted that combining optimal parameters could achieve high-efficiency ammonia synthesis of approximately ~ 730 ppm. By integrating a crystal graph CNN with DFT calculations, Boonpalit and co-workers [243] identified two high-efficient dual-atom catalysts (DACs) among 435 DACs in nitrogen-impregnated graphene for HER. Similarly, several human-based, AI-assisted approaches have been employed to explore exceptional single-atom catalysts [244]. Moreover, understanding the surface structure of heterogeneous catalysts, which can be achieved through data-driven ML methods, is crucial for bridging the material gap between theory and experiments [245]. Han et al., by identifying key descriptors based on the surface characteristics and guest atoms of single-atom catalysts, predicted four pivotal catalyst parameters. This approach led to the proposal of over 200 new single-atom catalyst candidates [246].

**Fig. 22:**
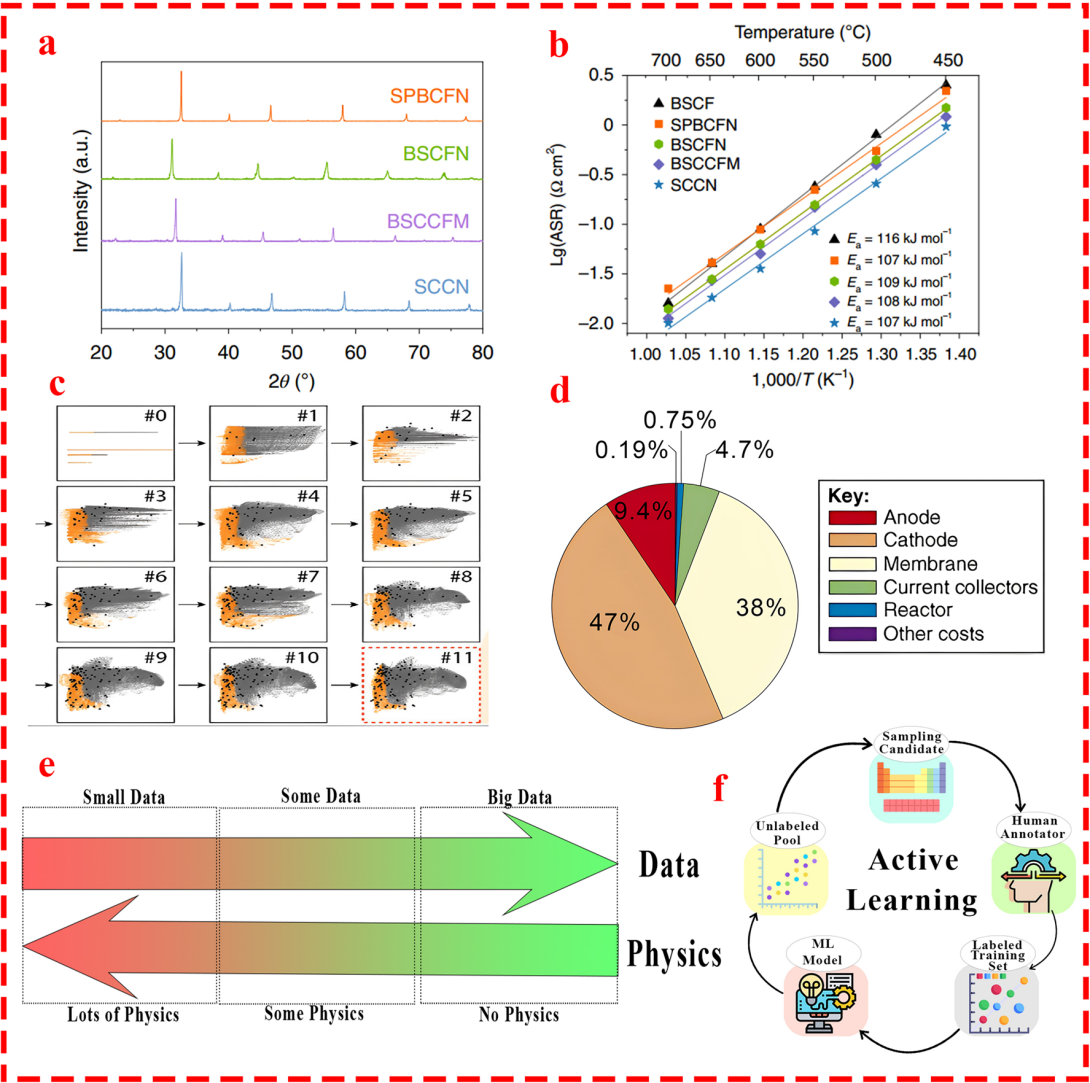
**a** XRD patterns of designed catalysts and **b** Arrhenius-type plots of ASR values of SCCN, BSCCFM, BSCFN and SPBCFN. Reprinted from [251] with permission from Nature Publishing Group. (The solid lines in b represent the least-squares fitting for the ASR values.) **c** Result of the Pareto active learning model for each step and the corresponding data points in 11 iterations. Reprinted from [263] with permission from John Wiley and Sons. **d** Cost distribution of various MEC components in laboratory scale. Reprinted from [206] with permission from Elsevier. **e** A general concept on physics and data relation for the concept of physics-informed machine learning. Reproduced from [255] with permission from Nature Publishing Group. **f** General approach of active learning method

However, the current form of data-driven ML mainly operates based on input and output relationships, which leave the black box nature of ML as a significant challenge for researchers. Indeed, a fundamental understanding of catalyst reactions, interactions of parameters, and the contribution of each factor can only be achieved by unraveling this "black box." As a solution, interpretable ML (ML models that allow researchers to understand how final decisions are made) has been proposed to shed light on the black box [247–249]. In fact, interpretable ML methods could play a vital role in the future discovery of catalysts with complex structures [250]. Interestingly, Zhai et al. [251] developed an interpretable ML method using Lewis acid as a descriptor to design high-performance cathode electrodes for solid oxide fuel cells based on perovskite oxides. They presented 6871 different perovskite compositions and went a step further than previous studies by experimentally validating their ML model. They fabricated four top candidates among all compositions with the formulas Sr_0.9_Cs_0.1_Co_0.6_Fe_0.3_Mo_0.1_O_3_ (SCNN), Ba_0.4_Sr_0.4_Cs_0.2_Co_0.6_Fe_0.3_Mo_0.1_O_3_ (BSCCFM), Ba_0.8_Sr_0.2_Co_0.6_Fe_0.2_Nb_0.2_O_3_ (BSCFN), Sr_0.6_Ba_0.2_Pr_0.2_Co_0.6_Fe_0.3_Nb_0.1_O_3_ (SBPCFN). The XRD analysis elucidated that all compositions comprised a pure cubic phase (Fig. 22a) that indicated the practical viability of ML-predicted catalysts. Among the predicted materials, the SCCN catalyst outperformed the others, making it the best candidate among the 6871 compositions tested (Fig. 22b). It is important to note that designing high-performance electrocatalysts is not only limited to the aforementioned system, but it successfully applied in other schemes such as lithium–sulfur batteries [252].

### Leveraging Physical Knowledge by Physics-Informed and Physical Model-Based Data Approaches

To enhance the accuracy and reliability of AI-driven models for proposing novel catalysts, incorporating recently developed AI approach such as physics-informed ML [253] offers a promising approach to bridging gaps in heterogeneous catalysts [254]. A physics-informed approach has the advantage of ensuring that entered data adheres to established physical models. This approach can be defined across three domains: from cases where there is abundant data but little understanding of physics, to those where there are limited data but comprehensive knowledge of physics (Fig. 22e) [255]. Most applications, including catalysts and chemical reactions, fall in the middle region, where some data and some physics are known. Examining macro-reaction kinetics, parameter identification, discovering reaction networks, and quantifying uncertainty in chemical reactions through multiphysics coupling can be effectively supported by physics-informed ML [256]. While the number of modeling studies in MECs compared to experimental research is very limited, particularly in multiphysics modeling, which is fewer than three studies, it is crucial that mathematical systems in MECs are expressed in dimensional space rather than time-dependent or simplified models for effective physics-informed ML applications. Physical model-based data augmentation is another powerful method for understanding physical knowledge before experimentation. Hong and co-workers developed an interpretable mathematical formula-based physical model integrated with ML to predict the adsorption energy and charge transfer of copper-based MOF catalysts. Their results demonstrated that the formula-based physical model outperformed neural network (NN) models by achieving high predictive accuracy with a low number of datasets. Interestingly, the study also revealed this approach’s extraordinary precision in detecting incorrect data. In their evaluation, 30 misleading and incorrect data points were added to the dataset. The findings showed that the formula-based physical model maintained nearly 100% accuracy, while the NN model's accuracy dropped drastically to 10% under the same scenario [257].

### Problems Associated with Small Datasets

As discussed earlier, data are the cornerstone of developing a robust and powerful AI model for fabricating catalysts. The dataset must be large enough to build a well-trained algorithm to be capable for accurately predicting and proposing high-performance structures. However, the challenge of acquiring large datasets remains a persistent issue for researchers using data-driven methods for catalyst performance prediction and fabrication. Pioneering methods such as U-Net, Graph Neural Networks, Active Learning, Generative Adversarial Networks, and Graph-Based Semi-Supervised Learning, among others, have emerged in response to this longstanding barrier [258]. Importantly, active learning is considered one of the main avenues for addressing small datasets in chemistry. Both active learning approaches (i.e., stream-based and pool-based) consist of five stages, as illustrated in Fig. 22f. Active learning has shown extraordinary potential in the fields of electrocatalysts for CO_2_ reduction [259, 260] and hydrogen production [261]. Moon et al. developed an active learning workflow for small datasets and discovered a novel, high-performance four-metal perovskite oxide electrocatalyst, Ca_0.8_Pr_0.2_Co_0.8_Fe_0.2_O_3-δ_ for OER. Unlike most ML-driven proposed catalysts, the exceptional performance of Ca_0.8_Pr_0.2_Co_0.8_Fe_0.2_O_3-δ_ could be qualitatively explained. However, contributions of limiting factors such as defect control, facet, and morphology require further advanced ML approaches in future studies [262]. Jung’s group [263] introduced a dual-purpose electrocatalyst composite for HER and OER using the Pareto active learning method. The model proposed a four-metal alloy, Pt_0.15_Pd_0.30_Ru_0.30_Cu_0.25_ which was validated experimentally. Their results determined that after 11 iterations, the Pareto active learning method significantly reduced discarded points for HER and OER overpotentials, with substantial changes from the first iteration decreasing by nearly 15% in the final iteration (Fig. 22c). Moreover, it has been demonstrated that mathematical, physics-based ML approaches offer significant advantages over traditional ML models for small datasets [257]. Surprisingly, Kim and co-workers developed an active learning ML model using only precursor composition as input data to predict a multi-metal alloy electrocatalyst for HER. Their model identified a ternary composition, Pt_0.65_Ru_0.3_Ni_0.05_, with a HER overpotential of 54.2 mV which outperformed the pure Pt catalyst [264]. Last but not least, the significance of active learning was not limited to the catalyst design since in other studies it was reported how implementing the active representation learning method could predict the reaction yield with limited datasets [265].

## Future Perspectives and Research Directions

**Fig. 23:**
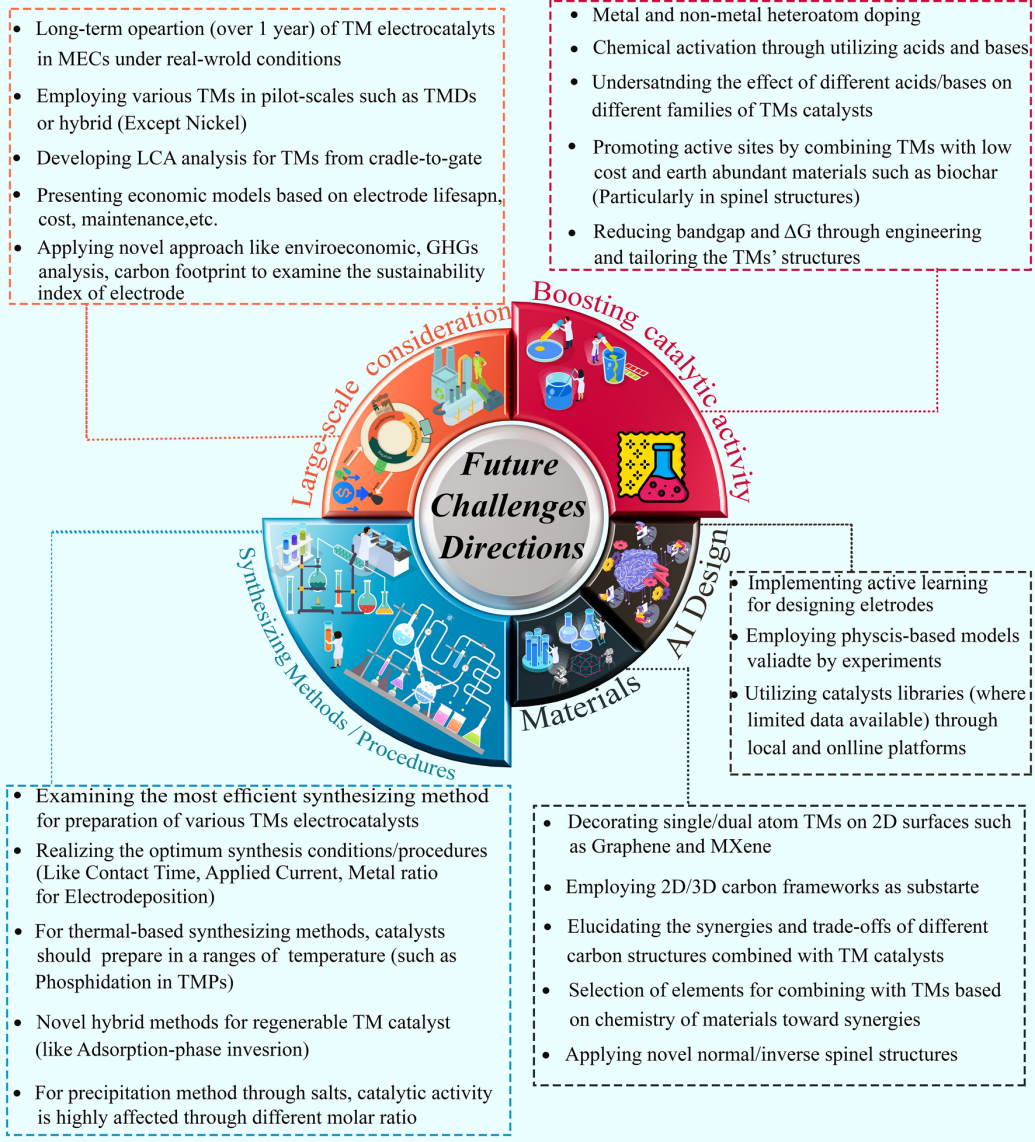
A conceptual framework for future directions and remaining challenges

Based on the critical discussion in this paper, a conceptual framework addressing the remaining challenges and future directions has been illustrated (Fig. 23). Furthermore, the following points can be highlighted as future research directions in the context of TM-based catalysts from different perspectives.

### Synthesizing Methods and Procedures

Selecting the most efficient synthesizing method can be considered the cornerstone of designing high-performance electrocatalysts, as different synthesizing routes lead to significantly different results [117, 127]. Among various synthesizing methods, hydrothermal and electrodeposition have shown superiority over others due to their ability to control synthesizing parameters. For instance, Logan's group [117] demonstrated that the hydrothermal method allows uniform distribution of Mo on the surface of Ni which results in abundant active sites compared to the electrode-assisted method. Moreover, spinel-type NiMoO_4_ catalysts prepared through the hydrothermal method form in the β-phase, whereas the electrode-assisted method results in the α-phase. Furthermore, in most studies that employed electrodeposition as the synthesizing method, constant conditions are applied for electrode fabrication. However, one critical aspect that should be reminded is that important parameters such as the applied current, synthesis time, and the ratio of transition metals significantly impact the electrocatalytic activity of MEC catalysts [180]. In the same manner, Hwang et al. [126] emphasized the significance of the applied current during the electrodeposition of MoS_2_ on catalytic activity. While electrodeposition is an efficient synthesizing method, future research is highly recommended to explore a range of conditions to determine optimal values for these parameters. Additionally, the temperature during thermal-based methods plays a pivotal role in promoting active sites and lowering the internal resistance of catalysts. For instance, during the phosphidation process in the fabrication of TM-based phosphide electrocatalysts, it has been observed that the temperature of the phosphidation process greatly influences the electrochemical features of the electrode. Thus, controlling the temperature and identifying its optimal value, particularly for TM-based phosphide catalysts, are crucial. Metal leaching is another challenge associated with metal-based catalysts. However, specific synthesizing procedures, such as those employed by Kim et al. [171], demonstrated that a sequence of adsorption and phase inversion synthesis can produce regenerable electrocatalysts with minimal metal loss under various pH conditions. Although this strategy is highly efficient for addressing metal leaching and maintaining catalytic activity during long-term operation, it has primarily been applied to hybrid TM-based electrocatalysts. This leaves an important research direction to evaluate its effectiveness for other TM-based electrocatalyst families (TMO, TMP, TMC, TMD, and TMN). Lastly, optimizing metal loading content is another pivotal factor that has proven to significantly influence the performance of electrocatalysts in MECs. It is strongly recommended to assess the optimal transition metal content in catalysts. Of particular interest, this holds true for transition metal catalysts synthesized via precipitation methods using salts, where different molar ratios during preparation result in distinct electrochemical behaviors.

### Implementing Novel Materials and Structures

From material perspective, a colossal number of candidates have yet to be examined, as they exhibited exceptional performance as electrodes, such as single-wall CNTs. Moreover, single-atom catalysts (SACs) and dual-atom catalysts with precise decoration on 2D surfaces have demonstrated superior catalytic activity compared to traditional catalysts—not only those utilized in MECs, but also in other applications such as ORR, CO_2_ reduction [266, 267], and water–gas shifting reactions. Hence, feasibility studies and experimental validation of these novel TM catalysts (such as Ni, Mo, Fe, Co, etc.) remain an unexplored direction requiring further research. For certain families of materials that are widely utilized as electrodes, managing trade-offs and synergies is essential. For instance, various TM-based electrocatalysts combined with carbon-based materials in different dimensions (0–3D) are commonly used in cathode electrodes. However, due to the wide range of carbon allotropes (in terms of cost, mechanical/chemical stability, conductivity, surface area, etc.), the suitability of each allotrope should be assessed based on the specific type of transition metal catalyst.

Importantly, spinel electrocatalysts in TM oxides have shown significant potential due to their unique crystalline structures, which offer high stability, excellent electronic properties, and enhanced charge transfer. Although several spinel electrocatalysts, such as NiMoO_4_, NiCoO_4_, ZnFe_2_O_4_, Co_3_O_4_, and Fe_3_O_4_, have been utilized in MECs, the number of studies on these efficient structures is limited. One of the most promising research directions is further exploration of other prominent spinel structures, such as Fe_3_O_4,_ which is earth-abundant, easy to prepare, and exhibits excellent stability. Another exciting research direction is investigating the impact of the degree of inversion in spinel structures (both normal and inverse spinels) on catalytic activity. Previous studies have demonstrated that the degree of inversion in spinels such as NiCo_2_O_4_ and ZnFe_2_O_4_ (which have shown promising results as electrocatalysts in MECs) [268, 269] influences electrical conductivity and charge transfer. This can provide an in-depth understanding of structural regulation and rational design for spinel catalysts in MECs. Additionally, introducing spinels into porous carbon frameworks (both 2D and 3D) has been shown to synergistically enhance the electrocatalytic activity of MEC electrodes [105, 106]. Hence, it is suggested not only to explore other spinel-type catalysts but also to evaluate their behavior on various carbon networks (rGO, graphene, graphite, etc.) to maximize catalytic activity.

For some TM-based catalysts, such as MOF-derived composites, challenges related to stability, long-term operation, and synthesizing methods remain as significant drawbacks that require further development. Unlike well-established materials like Ni-based alloys, many MOF-derived composites are still impractical for MECs. Regarding Tables 1 and 2, the operational periods for most studies are less than 15 days, with some extending up to 30 days (in limited cases several weeks). However, this duration is insufficient to assess the long-term performance of TM-based catalysts, particularly those MOF-derived structures.

Thus, it is highly desirable to conduct long-term experiments using TM-based electrocatalysts electrodes, which would provide two main benefits. The first is a thorough examination to elucidate the strengths and weaknesses of the electrodes from technical point of view. The second, and perhaps more important aspect, is the collection of extensive data during long-term operation, which could help researchers to apply data-driven, AI-assisted methods for fabricating future catalysts.

### Directions on Theoretical Studies and Structural Characterization

One of the most pivotal theoretical studies involves applying DFT to future TM-based catalysts, which remains very limited in the current state of the art. The DFT calculates the Gibbs free energy of hydrogen adsorption (ΔG_H*_) on electrocatalyst surfaces and serving as a descriptor for HER activity. It is worthy to mention that Gibbs free energy is a powerful approach and indicator in design of other renewable-based systems such as solar and geothermal energy [270, 271]. By analyzing electronic density states and surface reactivity, DFT provides insights into adsorption sites and catalytic performance, while also helping to determine optimal doping configurations, strain effects, and vacancy formations that influence the interaction of active sites with intermediate species. This approach guides the synthesis of TM-based catalysts with minimized overpotential and enhanced electron transfer. Aside from theoretical studies, catalyst characterization is another critical research direction, particularly for specific structures like spinels due to their unique features. Although we discussed on TM oxides with spinel and spinel-like structures (as these catalysts), only a limited number of studies have examined the characterization of catalysts to confirm whether the structure is spinel (since it could also take other forms, such as amorphous, even with the same formula) or not. Therefore, utilizing spinel-type electrocatalysts, given their specific features, presents numerous research opportunities for designing different spinel-type catalysts for MECs.

### Promoting Active Sites and Catalytic Activity

Strategies to promote catalytic activity and active sites could be considered as an integral step during the synthesizing process. However, as we focused on the pros and cons of each synthesizing method above, this section is explicitly assigned to possible strategies for the modification of electrocatalysts. Chemical activation with acids and bases is among the most facile and efficient blueprints to improve the active sites and catalytic activity by increasing the surface area and introducing functional groups. Previous studies have exhibited the exceptional potential of catalyst activation through chemical modifications. However, as highlighted, this strategy is rarely applied in designing TM-based electrocatalysts for MECs and has been explored in very limited studies [126, 169, 171]. Therefore, for future research, appropriate chemical activation through pretreatment and/or post-treatment (whether by acids or bases) is highly recommended. Particularly, this strategy is extraordinarily efficient when the TM-based catalyst is assisted by a carbon-based substrate since improving the electrocatalytic activity of carbon networks modified with a wide range of acids or bases has been demonstrated.

Another interesting strategy is non-metal (and in limited cases metals) heteroatom doping in TM-based catalysts, which effectively enhances electrochemical activity by improving the electronic structure (i.e., electrical conductivity, band structure, local charge distribution), physical properties (i.e., vacancy concentration, phase transformation), stability, and adsorption configuration, just to name a few [272, 273]. For instance, in the context of our study, introducing heteroatoms (particularly N, S, P) on the surface of 2D nanosheets facilitates the doping process due to their superior exposed surfaces and intrinsic defects. This is the reason why, in TM-based catalysts, N atoms are mainly doped on Mo_2_C [154, 163] and rGO [164]. This strategy remains limited in this context, thus, it is highly recommended to utilize non-metal heteroatoms in other TM-based catalysts, such as 2D MXene, as well as other TM-based electrocatalysts on carbon networks. Although doping and defect engineering are commonly applied with non-metal elements, doping highly conductive metal atoms, like Cu, is also suggested [118]. It is worth noting that selecting the type of dopant should depend on the desired feature (such as increasing active sites, improving stability, among others). However, when the substrate of the TM catalyst is carbon, nitrogen doping is the most suitable candidate for increasing active sites and improving electronic structures [31]. Eventually, co-doping and multi-doping of non-metal heteroatoms (e.g., N-S, N-P, or N-S-P), by providing synergies, represent another unexplored, low-cost research direction in this context.

Last but not least is utilizing biochar in TM-based electrocatalysts. Although, in limited studies, activated carbon (AC) has been applied in TM catalysts and exhibited superior performance, biochar is more attractive alternatives since they can deliver the same performance as AC but at an extraordinarily lower cost. Moreover, biochar has very simple preparation methods and a controllable nature of synthesis which allows their structure to be tailored based on the type of TM-based catalyst.

### Direction on Large-Scale Implication and Techno-Economic-Based Models

The long-term operation of TM-based electrocatalysts is one of the important problems in the existing literature. In fact, there are not enough studies on the long-term operation of TM catalysts beyond 12 months, making this an attractive research direction. Moreover, in the limited large-scale studies, the only transition metal used so far has been Ni. However, the problem with nickel is its high cost compared to traditional options like stainless steel. In several studies, other transition metals, such as TMDs and hybrid structures, have shown highly promising results at competitive costs, making the use of other transition metal catalysts in large-scale applications another attractive direction for translating novel high-performance catalysts into real-world applications. Furthermore, from LCA and cost analysis perspectives, the lack of novel approaches linking the process of manufacturing electrodes at large scales with their environmental impacts from energetic and environmental viewpoints highlights another critical research gap. Novel analytical approaches, such as enviroeconomic assessments, energy matrices, and GHG emissions (carbon footprint) [218, 219, 274], which connect LCA with cost and GHG emission/mitigation of energy systems at large scales while illustrating their sustainability could represent as another interesting direction for future work. Such analyses are particularly important for governments and stakeholders, offering insights into the broader impacts of deploying TM-based electrocatalysts on a larger scale.

### AI-Driven Fabrication for Design of TM-Based Electrocatalysts

The lack of sufficient data on TM-based electrocatalysts of MEC, due to limited studies and short-term operation, makes it challenging to implement ML methods for designing future electrodes. This necessitates the application of high-efficient ML methods for electrode design rather than traditional approaches. Furthermore, to overcome obstacles faced by previous ML-driven catalyst designs, it is essential to focus on developing interpretable ML approaches, which would represent a significant step forward in the rational design of TM-based electrodes. Another important direction is applying multiphysics electrode modeling approaches and integrating the results with physics-informed neural networks. This would not only enable the design of high-performance cathodes but provide a clearer view of the contributing parameters during biohydrogen production. Moreover, integrating advanced computational methods, such as DFT, with ML approaches could build a framework for designing cathodic materials. However, experimental validation is crucial for these approaches to be effective. One of the primary issues in previous catalyst design studies is that most ML-derived structures were not experimentally validated. Given the limited data on TM-based electrodes in MEC, implementing mathematical formula-based physical models in ML approaches is highly recommended, as they excel in training with smaller datasets. Importantly, active learning is one of the most promising approaches and is predicted to play a pivotal role in designing future catalysts through AI [259]. Indeed, active learning has outperformed many of the advanced ML methods for designing catalysts either for large or small datasets. Since the “Achilles' heel” of applying ML models in chemistry is the scarcity and reliable datasets—this issue is even more worsen in MEC compared to other BESs like MFC—implementing active learning for MEC's catalysts design appears to be imperative and inevitable. As the field of AI rapidly advances, exploring new frontiers such as fully autonomous laboratories—employing robots and ML methods to accelerate the discovery of new materials [275]—electrochemical science is poised to undergo a revolution in the near future through advanced interpretable ML models [276]. Thus, it is worth emphasizing that applying ML to MEC development should not be limited to electrode design but can also extend to other components of MECs, such as optimizing and regulating the electrolytes characterization (a key factor in complex chained reactions) before and during operations, engineering biofilms, or modifying high-efficient anodes. For instance, several ML-driven studies have successfully predicted and interpreted complex reactions in aqueous media [277, 278]. Although this topic lies outside the scope of this review, the importance of ML-based studies on complex electrolytes and their interactions with microorganisms and electrodes deserves further investigations. Integrating these AI-based models for electrode fabrication and electrolyte optimization could create synergistic effects which could pave the way for a brighter future for MECs in real-world applications.

## Conclusion

The role of advanced TM-based electrocatalysts in the development of MECs, as one of the future alternative systems for biofuel production and wastewater treatment, is of great importance. Significant attention has been focused on cathode materials—the main site of hydrogen production—as the development of high-performance electrodes remains one of the primary obstacles hindering the real-world application of MECs. On the other hand, the utilization of novel structures and advanced TM-based electrocatalysts in MECs has been largely overlooked in recent years. This issue is even more disturbing when considering that the application of AI-driven methods for designing and fabricating novel electrodes has also been completely neglected. In this context, our main approach in this review was to open new avenues for future studies while providing a roadmap that leverages the advantages of previous efforts in design and experiments of electrocatalysts in MEC toward managing synergies and managing trade-offs.
